# Macrocycles in Drug Discovery—Learning from the Past for the Future

**DOI:** 10.1021/acs.jmedchem.3c00134

**Published:** 2023-04-05

**Authors:** Diego Garcia Jimenez, Vasanthanathan Poongavanam, Jan Kihlberg

**Affiliations:** † Department of Chemistry-BMC, Uppsala University, Box 576, SE-751 23 Uppsala, Sweden; ‡ Department of Molecular Biotechnology and Health Sciences, University of Torino, Quarello 15, 10135 Torino, Italy

## Abstract

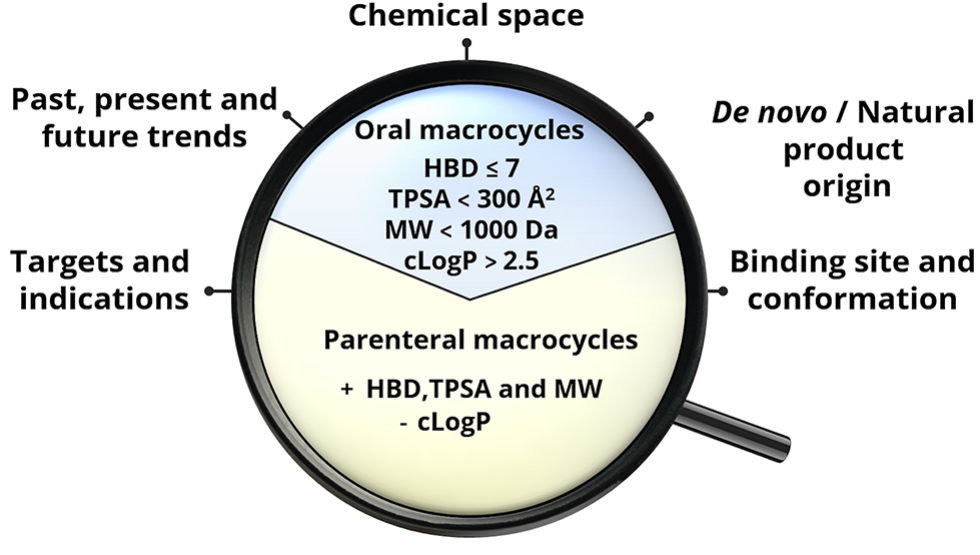

We have analyzed FDA-approved macrocyclic drugs, clinical candidates, and the recent literature to understand how macrocycles are used in drug discovery. Current drugs are mainly used in infectious disease and oncology, while oncology is the major indication for the clinical candidates and in the literature Most macrocyclic drugs bind to targets that have difficult to drug binding sites. Natural products have provided 80–90% of the drugs and clinical candidates, whereas macrocycles in ChEMBL have less complex structures. Macrocycles usually reside in the beyond the Rule of 5 chemical space, but 30–40% of the drugs and clinical candidates are orally bioavailable. Simple bi-descriptor models, i.e., HBD ≤ 7 in combination with either MW < 1000 Da or cLogP > 2.5, distinguished orals from parenterals and can be used as filters in design. We propose that recent breakthroughs in conformational analysis and inspiration from natural products will further improve the de novo design of macrocycles.

## Introduction

The search for innovative ways to modulate novel and challenging drug targets has led to a soaring interest in new therapeutic modalities among biopharmaceutical companies and academics involved in drug discovery.^1,2^ Macrocycles and cyclic peptides, proteolysis-targeting chimeras (PROTACs), and oligonucleotides are prominent examples of new modalities, all of which reside in chemical space beyond the Rule of 5 (bRo5).^3,4^ Even though all types of new therapeutic modalities have not yet reached the market, the efforts to modulate novel targets have already resulted in oral drugs having increased in size and complexity since the beginning of this century.^5,6^

**Figure 1:**
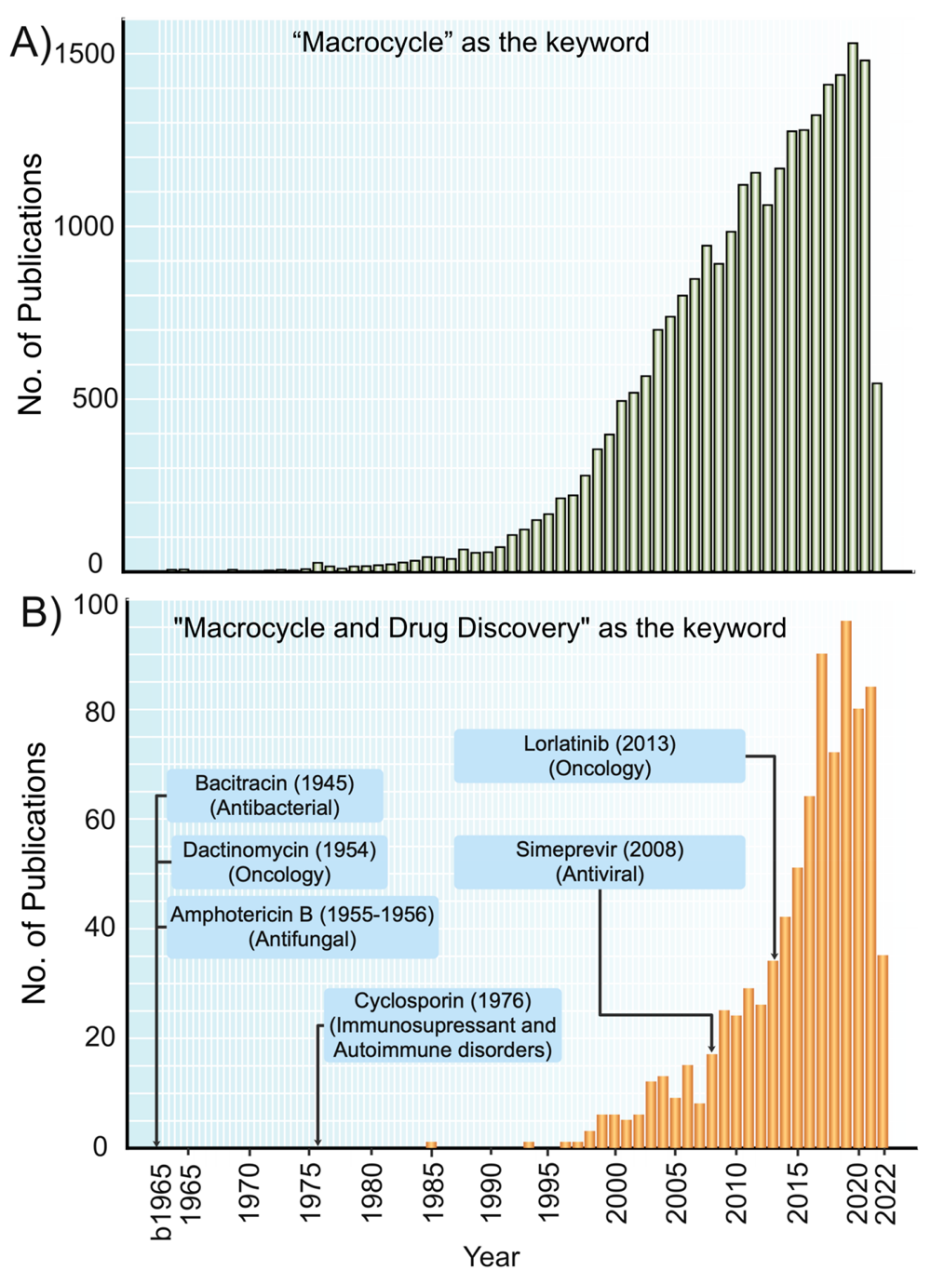
Number of articles retrieved from PubMed^16^ each year using (A) “Macrocycle” as the keyword and (B) “Macrocycle and Drug Discovery” as keywords (last downloads May 2022). Some examples of macrocyclic drugs of natural product origin (bacitracin, dactinomycin, amphotericin B, and cyclosporin) and also some obtained by de novo design (simeprevir and lorlatinib), their therapeutic indication, and the year of their first report in the literature have been included.

Macrocycles are generally defined as organic molecules which contain a ring of at least 12 heavy atoms. The general interest in macrocycles across different fields of science started to grow some years before 1980 and then increased dramatically from 1990 and onward (Figure 1A). In drug discovery, the rapid growth phase began at the turn of the millennium (Figure 1B). In fact, the number of publications per year has increased 10–20 times since then. The benefits of macrocycles originate from the fact that they can provide functional diversity and stereochemical complexity in a semirigid, preorganized structure. As compared to ring-opened analogues, this can allow macrocycles to bind with higher affinity and selectivity to targets that are difficult to drug with more traditional small-molecule drugs.^7−9^ Matched pairs of macrocycles and linear controls reveal that up to 100-fold potency increases can be obtained,^10,11^ but it should be noted that negligible or small differences in potency have been found for other matched pairs.^12,13^ In spite of their size, macrocycles may also have sufficient cell permeability and bioavailability to reach intracellular targets after oral administration.^8,14,15^ Historically, macrocyclic drugs have been provided by nature,^7,14^ but de novo designed macrocycles have now began to become approved as drugs (Figure 1B).

The rational design of potent, cell-permeable, and orally available macrocyclic drugs has many unknowns to be resolved, and moreover, their synthesis is far from being a trivial task.^17,18,9^ Conformational restriction by macrocyclization can provide potent ligands for difficult to drug targets that have flat or shallow binding sites^3,19,20^ but results in ring strain, steric interactions, and noncovalent transannular interactions that make the prediction of conformations and subsequently of molecular properties extremely challenging.^21−23^ In addition, some macrocycles have the capacity to adapt their conformations to the environment, thereby behaving as molecular chameleons.^24−26^ Chameleonicity is assumed to be a particularly useful characteristic of compounds in the bRo5 space, which improves their possibility to balance aqueous solubility and passive cell permeability. However, even though some promising progress in general methodology has been made recently^27,28^ and specific examples have been reported,^29−32^ the design of macrocyclic molecular chameleons is still poorly understood. Just as close to a decade ago,^14^ the design of macrocyclic drugs therefore still benefits from simple guidelines based on readily calculated descriptors in order to improve the chances of successfully engineering their pharmacokinetic properties.

In this Perspective, we describe the state of the art in macrocycle drug discovery. We first give an overview of the macrocyclic drugs approved by the FDA focusing on their therapeutic indications and the nature of the targets they modulate. Calculation of a set of 2D molecular descriptors allowed the dissection of chemical space of oral and parenteral macrocyclic drugs and the formulation of biproperty guidelines to help medicinal chemists design the next generation of oral macrocycles. In addition, mining of the literature and analysis of the macrocycles in clinical studies provide some hints on future trends in macrocycle drug discovery. Last but not least, we discuss how recent developments may improve the design of macrocycles with tailored properties and that a resurging interest in natural products may boost macrocyclic drug discovery.

## Results and Discussion

### FDA-Approved Macrocyclic Drugs

#### Overview

**Figure 2:**
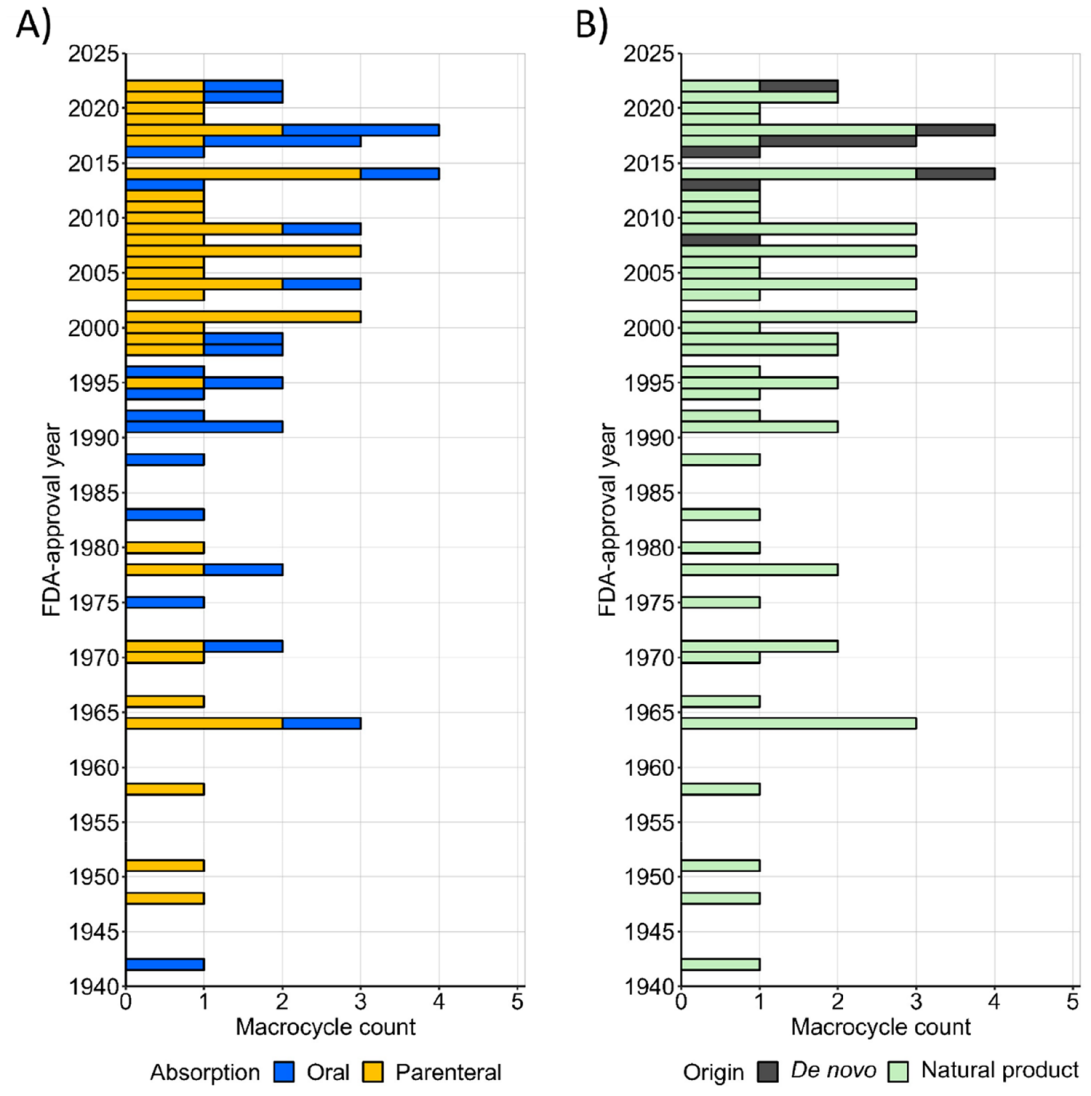
Number of macrocyclic drugs plotted by their year of approval by the FDA (*n* = 67, data retrieved on September 1, 2022). (A) Orally absorbed drugs are indicated in blue (*n* = 26; 39%), while those administered parenterally are in gold (*n* = 41; 61%). (B) Natural products and derivatives thereof are presented in light green (*n* = 59, 88%); de novo designed macrocyclic drugs are in dark gray (*n* = 8, 12%). Contrast agents, macrocycle-conjugated antibodies, PEG-linked macrocycles, and cyclodextrins have been excluded.

In total, 67 macrocycles have been approved as drugs by the U.S. Food and Drug Administration (Figure 2, Table S1).^33^ Approval of macrocyclic drugs fluctuated significantly over time until 1990, but since then, at least one macrocycle has been approved each year with only a few exceptions.^34^ Twenty six of the macrocyclic drugs (39%) are dosed orally for systemic distribution to their site of action, while 41 are administered parenterally. Although oral dosing is the preferred route of administration, the proportion between orals and parenterals has remained constant over time. The vast majority of the macrocyclic drugs are natural products or derivatives thereof (*n* = 59, 88%). In fact, the first de novo-designed macrocyclic drug, plerixafor, was approved in oncology as late as in 2008. Subdivision of the natural products into the original natural products (*n* = 25) and natural product derivatives (*n* = 34) allowed analyses of the improvements achieved by producing the derivatives (Table S2). Twelve natural product derivatives originated from the optimization of pharmacokinetics. Thus, oral bioavailability and/or half-life was improved for seven antibacterial erythronolide and rifamycin derivatives as well as for everolimus, which is used in oncology and as an immunosuppressant. Chemical stability, resistance to proteases, and solubility were improved for three other derivatives. Pharmacodynamics, i.e., improved potency, a wider spectrum of activity, and/or reduced side effects, constituted the main reasons for generation of seven derivatives. Both pharmacodynamics and pharmacokinetics were optimized for various reasons for five derivatives, while we were unable to find a clear explanation for what was achieved with the remaining 10 derivatives. As will be discussed further below, macrocycles provide unique opportunities to modulate targets with difficult to drug binding sites,^6,35^ but in spite of that, only 4% of the drugs approved by the FDA (*n* = 1796, biologics and drugs for veterinary use have been excluded) are macrocycles. Altogether, this indicates that it is desirable to improve design strategies to enable delivery of increased numbers of orally absorbed macrocyclic drugs.

#### Therapeutic Indications and Targets

**Figure 3:**
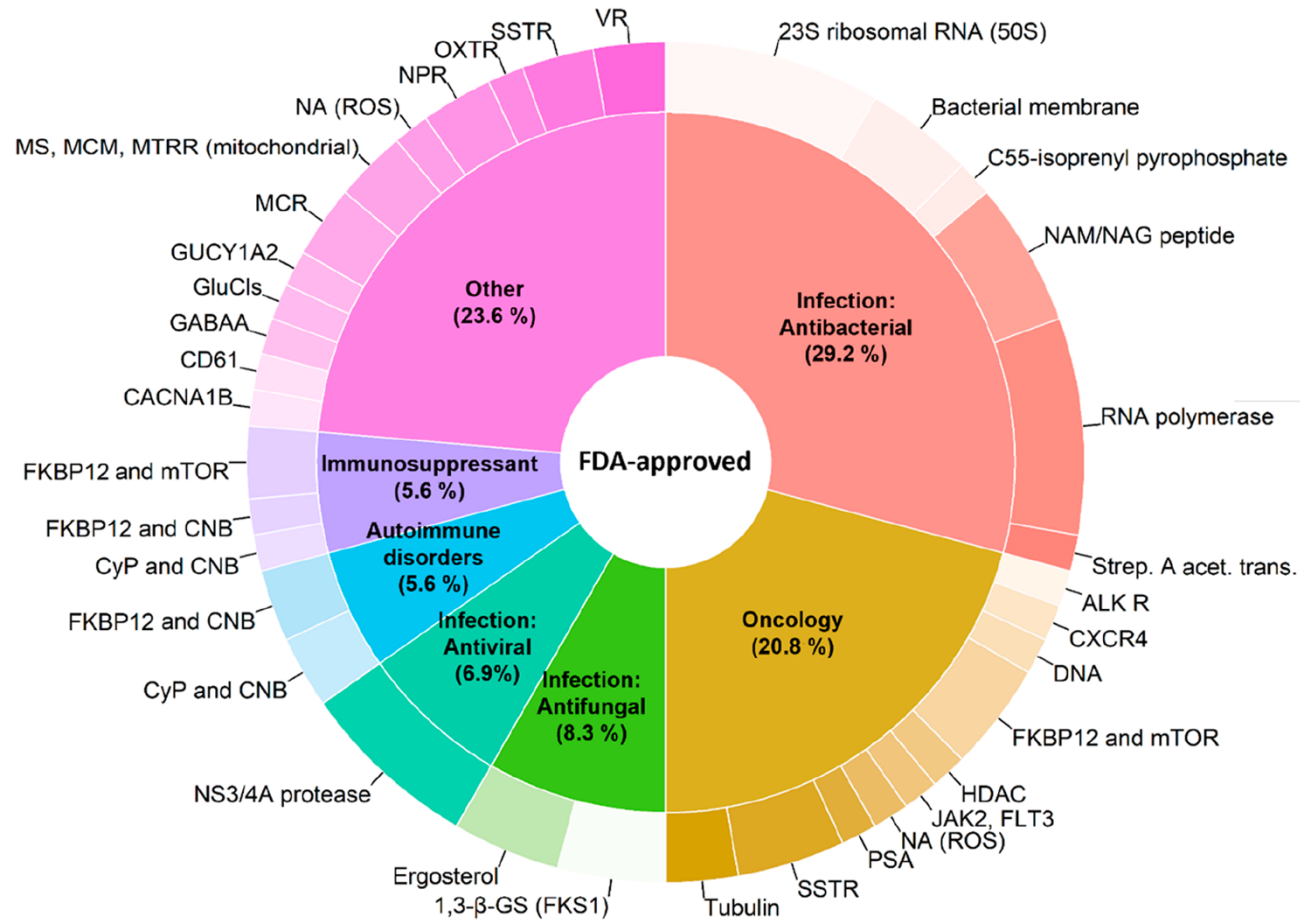
Therapeutic indications (inner circle) and targets (outer circle) of the macrocyclic drugs approved by the FDA (*n* = 72). Five macrocycles are duplicated because each one is used for two therapeutic indications. Therapeutic indications treated by <4% of the total number of macrocyclic drugs have been grouped under “Other”. Targets separated by “and” indicate that the corresponding drug is a molecular glue, while targets separated by a comma indicate that the corresponding drug displays polypharmacology. NA: Target not available. A complete list of therapeutic indications and targets for the macrocyclic drugs is provided in Table S1.

Infectious disease is the major therapeutic indication treated by macrocyclic drugs (44.4% of all macrocyclic drugs, Figure 3, Table S1). Within this class, most are used as antibacterial agents, but antivirals (6.9%) and antifungals (8.3%) are also important. Oncology (20.8%), autoimmune disorders (5.6%), and immunosuppressants (5.6%) are the three other major therapeutic indications. Macrocyclic drugs are also used in 13 “Other” minor indications, representing 23.6% of the drugs (Figure 3, Table S1). These indications include antidiuretics, chronic pain, genetic obesity, heart failure, etc. The approval of macrocyclic drugs over time reveals several trends (Figure S1). First, antibacterials have been approved with a fairly regular frequency since 1948. Second, the five macrocycles used to treat hepatitis C virus infections were all approved between 2013 and 2017. Finally, there has been an upsurge in the use of macrocycles in oncology in recent years, i.e., 4 were approved prior to 2007, while 11 have been approved since then.

The number of targets drugged by macrocycles displays a heterogeneous pattern between the major therapeutic indications. Antibacterial macrocycles are mainly directed toward a few “traditional” targets, such as the ribosome and RNA polymerase (RNAP) (Figure 3). In contrast, in oncology macrocycles are directed toward a larger number of targets, which include kinases, deacetylases, hormone receptors, and tubulin. Pacritinib, which was approved in 2022 as the first dual inhibitor of Janus kinase 2 (JAK2) and FMS-like receptor tyrosine kinase-3 (FLT3),^36^ exemplifies how macrocycles may be used in a target-rich indication. It is noticeable that the NS3/4A protease of the hepatitis C virus is the only viral target modulated by macrocyclic drugs. We also emphasize that several macrocyclic drugs act as molecular glues^37^ that form ternary complexes with pairs of protein targets (Figure 3 and Table S1). Complexation of cyclophilin A (CyPA) and calcineurin B (CNB) by cyclosporin and voclosporin is used for treatment of autoimmune diseases and for immunosuppression to prevent rejection of transplanted organs. Ternary complex formation of the FK506-binding protein FKBP12 with either CNB or mammalian target of rapamycin (mTOR), induced by the macrocycles of the ascomycin and rapamycin families, is used in autoimmune diseases, for immunosuppression, and in oncology.

### Drug–Target Structures

#### Binding Site Shape

**Figure 4:**
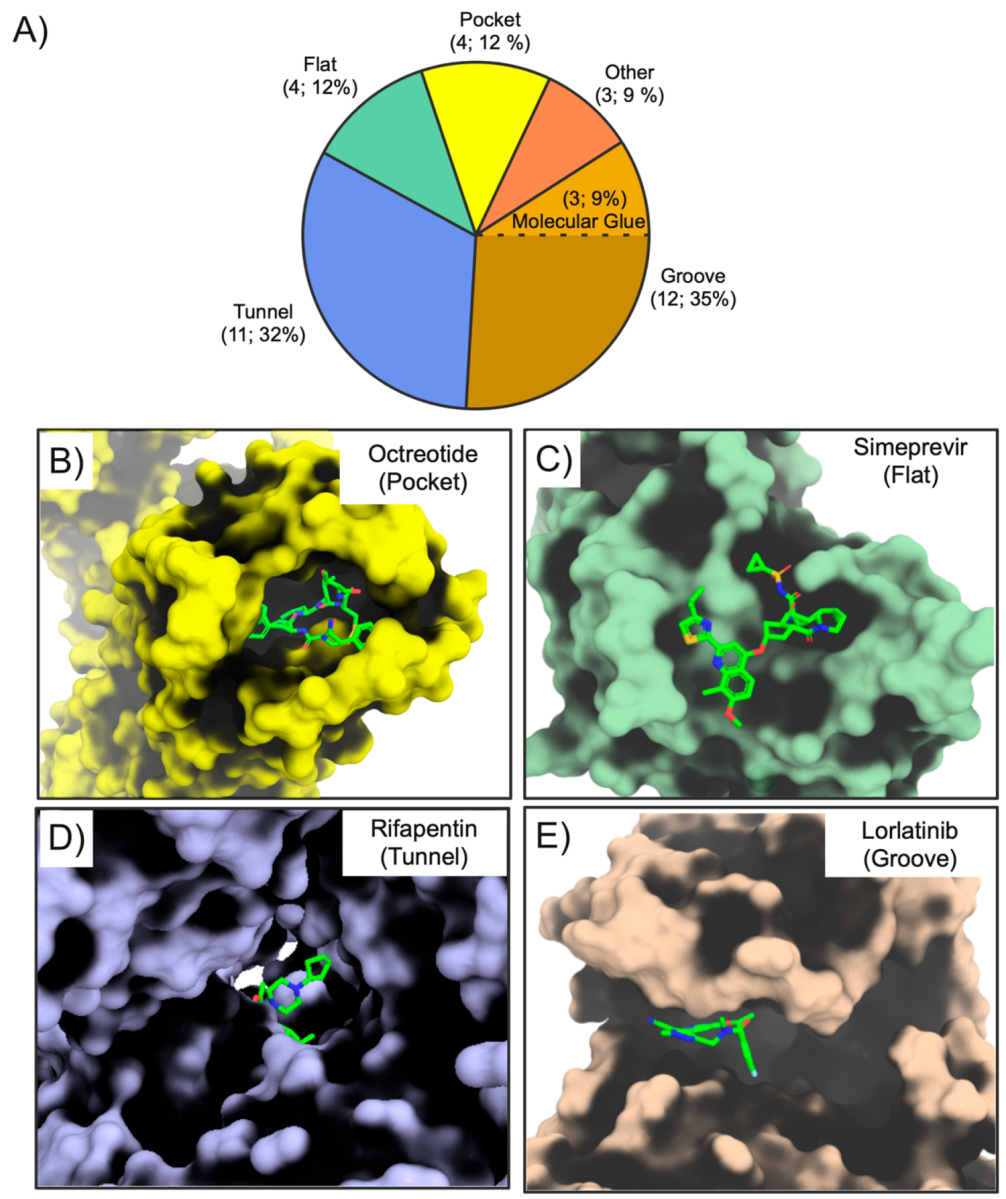
(A) Classification of the shape of the binding sites in the crystalline complexes of macrocyclic drugs with their targets (*n* = 34). The number of drugs bound to each binding site class and their percentages of the total are given in parentheses. Examples of drug–target complexes in which the macrocycle binds to a pocket (B), flat (C), tunnel-shaped (D), or groove-shaped (E) binding site. The macrocyclic ligands are displayed as green sticks with nitrogen atoms in blue, oxygen in red, and sulfur in yellow. The selected examples are octreotide bound to the somatostatin receptor 2 (SSTR2, PDB ID: 7T11), simeprevir bound to the HCV NS3/4A protease (PDB ID: 3KEE), rifapentin bound to RNAP (PDB ID: 2A69), and lorlatinib bound in the groove of the ALK (PDB ID: 4CLI).

Macrocyclization restricts the flexibility of a ligand and has been emphasized as a tactic to discover ligands for targets that are “difficult to drug”, in particular, targets that have large flat, groove-shaped, or tunnel-shaped binding sites.^7,35,6,38^ To investigate to what extent the macrocyclic drugs in our data set bind to targets with “difficult” binding sites, we searched the PDB^39^ for target-bound complexes of each drug. Then, the shape of the binding site of each target was manually classified as described earlier (cf. Methods).^6^ Macrocycle–target complexes were available for 34 out of the 67 macrocyclic drugs. Interestingly, the majority of these complexes (27 out of 34) had the macrocycle bound in a binding site belonging to a difficult to drug category, i.e., a flat, groove-shaped, or tunnel-shaped site (Figure 4A, Table S3). Four of the macrocycles also bind in pockets, and three exert their pharmacodynamic effects in other ways than by interacting with a well-defined binding site.

Most of the macrocyclic drugs reside in bRo5 space, and their size provides one explanation for why they have sufficient affinity for targets having “difficult” binding sites.^6^ Ro5-compliant compounds can usually bind with sufficient affinity in pockets to serve as drugs, but the complex of octreotide bound to the somatostatin receptor 2 illustrates how macrocycles can fill large pockets (Figure 4B). Simeprevir and rifapentin exemplify how macrocycles in bRo5 space can engage targets that have flat or tunnel-shaped binding sites, respectively (Figure 4C and 4D). It should be noted that only the four HCV NS3/4A protease inhibitors bind to a flat binding site in the set of FDA-approved drugs and that most of the macrocycles that bind in a tunnel are antibacterials. Although the majority of the macrocycles that bind in grooves reside in bRo5 space, it is interesting to note that lorlatinib and ixabepilone also bind in grooves. These two macrocycles comply with the Ro5 and Veber’s rules (MW = 406 Da for lorlatinib, MW = 507 Da for ixabepilone) but still provide sufficient affinity to function as effective drugs (Figure 4E). It is also worth noting that three of the groove-shaped binding sites involve ternary complexes, i.e., the macrocycle acts as a molecular glue^37^ for two proteins that form the groove. Specifically, the three molecular glues are cyclosporin in complex with cyclophilin A (CyPA) and calcineurin B (CNB), tacrolimus with the FK506-binding protein FKBP12 and CNB, and sirolimus with FKBP12 and mammalian target of rapamycin (mTOR).

#### Ligand Shape

**Figure 5:**
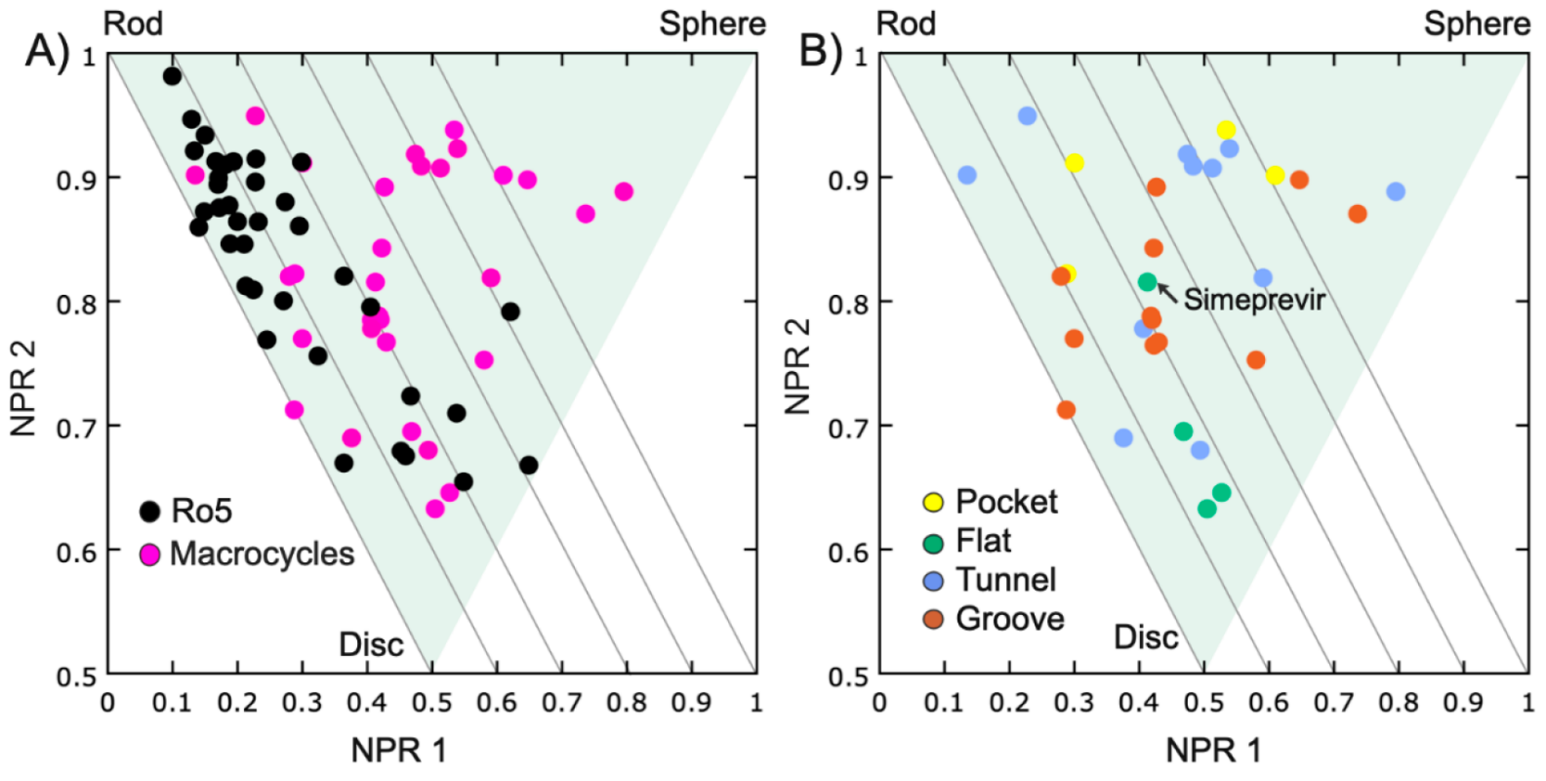
Normalized principal moments of inertia (PMI) plot illustrating (A) the shapes of the target-bound conformations of macrocyclic drugs bound to targets that have flat, groove, tunnel, or pocket-shaped binding sites (*n* = 31) compared to the shapes of a fully Ro5 compliant reference set of drugs (*n* = 37)^6^ and (B) the shape of the target-bound macrocyclic drugs, colored by the shape of their binding site. All four macrocycles that bind to a flat binding site are inhibitors of the NS3/4A protease of the hepatitis C virus.

We analyzed the shape of the macrocyclic ligands in their target-bound conformations using a normalized principal moment of inertia (PMI) plot, which classifies the ligands based on their similarity to a rod, disc, or sphere (Figure 5). Comparison to a reference set of Ro5-compliant drugs confirmed earlier reports^6^ that macrocycles populate spherical conformations to a larger extent than Ro5-compliant drugs, which are mainly rod–disc shaped (Figure 5A). The macrocycles that adopt spherical conformations were mainly bound in grooves and tunnels (Figure 5B). Except for simeprevir, the hepatitis C virus inhibitors adopt a disc shape, which matches the rather flat binding site of the NS3/4A protease (Figure 5B). Simeprevir differs from the other three inhibitors since it has both the aromatic and the acylsulfonamide side chain perpendicular to the plane of the macrocyclic core (Figure S2).

### Chemical Space of Macrocyclic Drugs

#### By 2D Descriptors

Although 2D molecular property descriptors have been suggested to be less informative than 3D descriptors for the characterization of drugs in the bRo5 chemical space,^21,40^ they are straightforward to calculate and can provide valuable information including comparisons to Ro5-compliant drugs.^14,3^ Consequently, we selected a set of 10 descriptors that were calculated for the macrocyclic drugs data set and then used throughout the chemical space analysis, i.e., molecular weight (MW) and number of carbon atoms (nC) for size, calculated partition coefficient between octanol and water (cLogP) and number of aromatic rings (NAR) for lipophilicity, topological polar surface area (TPSA), hydrogen-bond donors (HBD) and hydrogen-bond acceptors (HBA) for polarity, and number of rotatable bonds (NRotB) and Kier’s flexibility index (Φ) for flexibility. Calculated solubility (cLogS) was also included. Chemical structures were adapted to the major charge state at pH 7.0 in MarvinSketch to provide an appropriate description of the macrocycles in a physiological, aqueous environment. Then, descriptors were calculated in Dragon, which identifies HBAs somewhat differently than the Lipinski’s Rule of 5 (cf. Methods; chemical space analysis).^41^ For these two reasons, HBD, HBA, and TPSA have different values than if calculated for neutral species as performed by Lipinski and Veber.^41,42^ For example, a secondary aliphatic amine contributes one HBD according to the Rule of 5 but two if it is predicted to be positively charged at pH 7.0. Macrocycles that contain metal complexes were removed as they introduce errors in the calculated descriptors, and only the major component of drugs consisting of mixtures was included.

**Figure 6:**
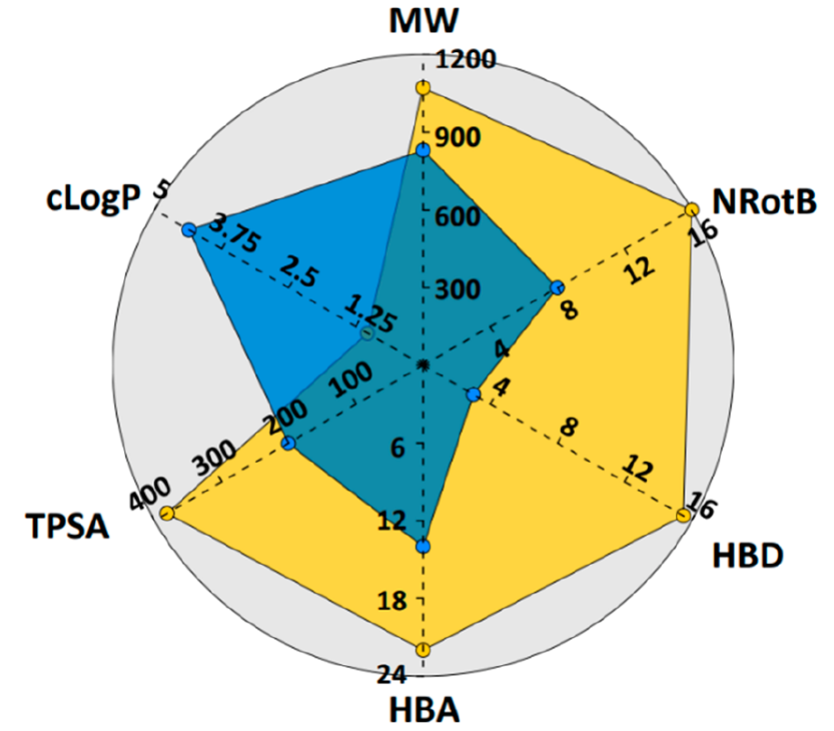
Radar plot comparing the median values for the descriptors employed in Lipinski’s Ro5 and Veber’s rule for the oral (blue, *n* = 24) and parenteral (gold, *n* = 38) subsets of FDA-approved macrocyclic drugs. Note that HBD, HBA, and TPSA were calculated differently than in the original rules (cf. Methods).

As expected, there was a clear separation between the property space of the orally and parenterally administered subsets of macrocycles (Figure 6, Table S4, and Figure S3). The oral drugs were significantly smaller (MW), more lipophilic (cLogP), less polar (TPSA, HBA, HBD), and less flexible (NRotB). These findings are in general agreement with the need for oral drugs to display both a satisfactory cell permeability and aqueous solubility by balancing lipophilicity and polarity. However, the majority of the oral macrocycles reside far into the bRo5 chemical space for several descriptors, i.e., MW, TPSA, and HBA. It is often assumed that compounds in the bRo5 space may achieve satisfactory cell permeability and solubility by behaving as molecular chameleons that adapt their conformations to the surrounding environment.^25,21^ Experimental support for chameleonic behavior has been reported for several macrocyclic drugs in the bRo5 space, with cyclosporin being studied extensively and others such as roxithromycin, telithromycin, spiramycin, and simeprevir being investigated recently.^26,24,43^

#### By Principal Component Analysis

**Figure 7:**
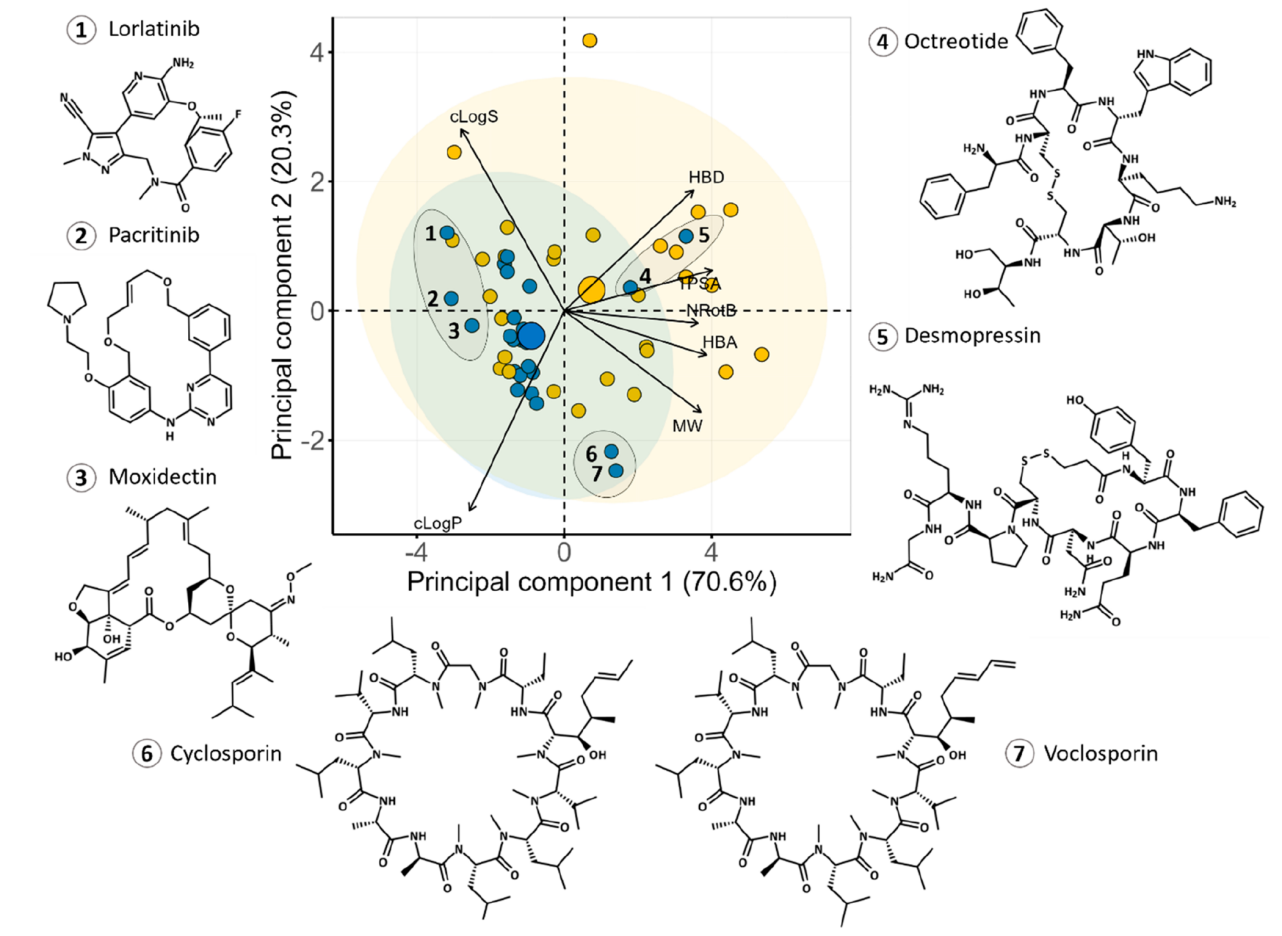
Principal component analysis of the chemical space of the macrocyclic drugs data set (*n* = 53). The PCA was based on the descriptors of Lipinski’s^41^ and Veber’s^42^ rules, as well as cLogS, calculated at pH 7.0. Ellipses in blue and yellow shading show the 95% confidence intervals for orally and parenterally administered macrocycles, respectively. The centroid of each class is indicated with a large circle in the color of the respective class. The contributions of individual descriptors to the PCAs are indicated by the length of the arrows. The structures of three Ro5 compliant macrocycles (**1**–**3**), two analogues of cyclic peptide hormones (**4** and **5**), as well as cyclosporin (**6**) and voclosporin (**7**) are provided. Nine parenterals with MW > 1500 Da were excluded in the PCA to provide a better dissection of the chemical space of the orally bioavailable macrocycles (cf. Figure S4 for the PCA for the complete set of macrocycles (*n* = 62)).

We used principal component analysis, an unsupervised machine learning method used to reduce the dimensionality of data, as an alternative way to probe the chemical space of the orally and parenterally administered macrocycles than by inspection of the descriptors of Lipinski’s Ro5 and Veber’s rule (Figure 7).^41,42^ Since no orally absorbed drugs are found at MW > 1500 Da, we excluded nine parenterals with MWs above this cutoff to provide a better dissection of the chemical space of the orally bioavailable macrocycles (the PCA for all macrocyclic drugs is found in Figure S4). In addition, three descriptors that provided redundant information (nC, NAR, and Φ) were not used. The first two components of the PCA explained up to 91% of the variability and allowed us to identify different but superimposable regions for the oral and parenteral subsets (cf. blue and golden ellipses, Figure 7). Just as revealed by the analysis of the different descriptors, parenteral macrocycles were in general located in more polar chemical space than orally administered ones. Accordingly, lipophilicity (cLogP) was found to be the descriptor making the largest contribution to the PCA, while flexibility (NRotB) was the least important.

The PCA revealed that the oral macrocycles (*n* = 26) were located in four regions of chemical space (Figure 7). First, most of the orals were found in a region close to the center of the ellipsoid describing the chemical space of oral macrocycles. Second, lorlatinib, pacritinib, and moxidectin have lower MW, cLogP, HBA, and NRotB than the major oral cluster. These three macrocycles reside in Ro5 space, and lorlatinib even allows treatment of cancer metastasis in the brain.^44^ Cyclosporin and voclosporin are cyclic peptides with high MW, TPSA, HBA, and NRotB that reside in a third region of chemical space. Cyclosporin has been proven to behave as a molecular chameleon, and this property is generally assumed to be of major importance for its high but variable bioavailability (up to 60%).^26^ Voclosporin is a derivative of cyclosporin with a single modification in residue 1, which can also be expected to benefit from behaving as a molecular chameleon. Lastly, the fourth region of the oral space consists of desmopressin and octreotide, two cyclic peptides with very high polarity (TPSA and HBD), high MW, and NRotB count. Despite its low bioavailability (*F* < 0.16%), the high potency of desmopressin allows its use as an oral drug.^45^ Similarly, oral administration is an alternative to the subcutaneous route for octreotide in spite of its low bioavailability (*F* = 4%).^46^

As natural products dominate among the macrocyclic drugs, we also used the PCA to understand if optimization of the original natural products led to the natural product derivatives occupying a different chemical space and if this space was closer to that of the de novo-designed drugs (Figure S5). As all but one of the eight de novo-designed macrocycles are administered orally, it is unsurprising that they occupy part of the oral chemical space which is located closer to the Ro5 space. The chemical space of the original natural products overlaps completely with that of the derivatives, but due to a few outliers, the chemical space of the original natural products is larger. In line with this, the PCA does not indicate that optimization has driven the derivatives toward the Ro5 space, a conclusion which is independent of whether both parenteral and orally administered macrocycles or only the oral ones are considered.

#### Classifying Oral versus Parenteral Macrocycles

**Figure 8:**
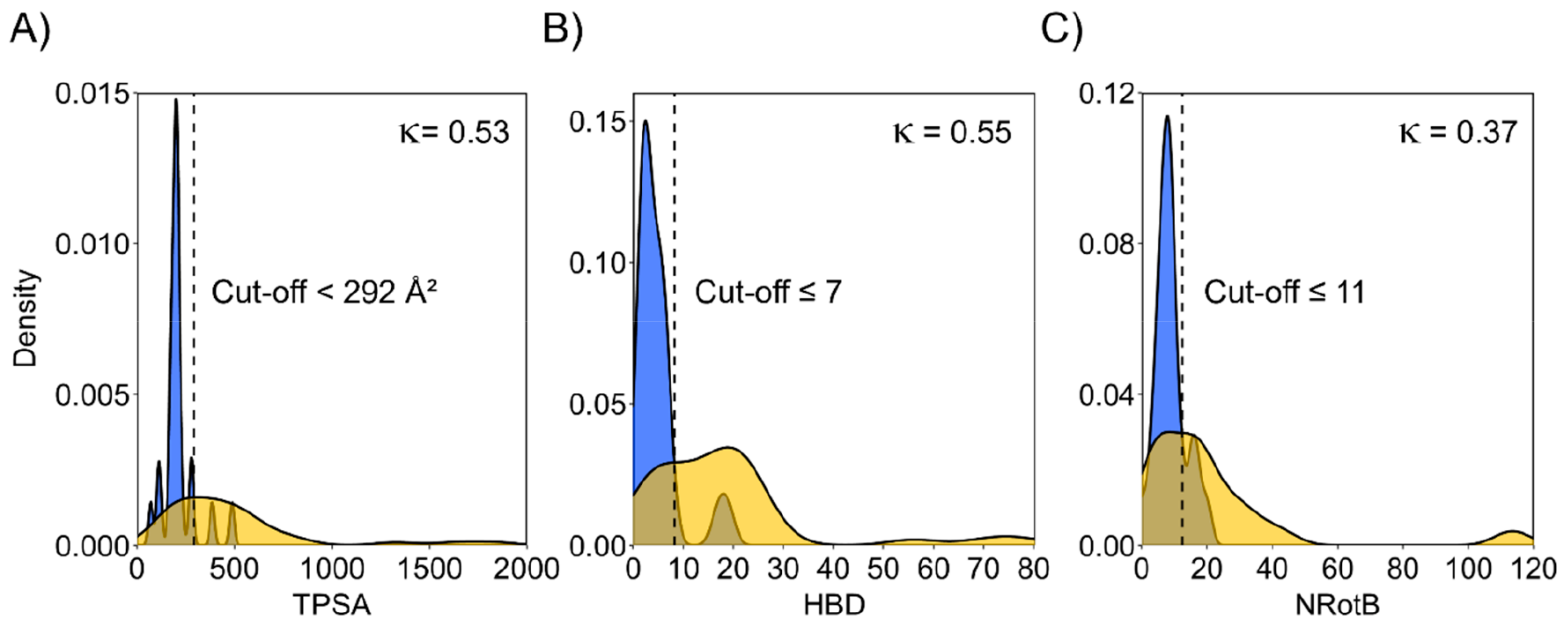
Single-property distributions for HBD (A), TPSA (B), and NRotB (C) for the oral (blue) and parenteral (gold) subsets of the macrocyclic drugs training set (*n* = 62). The black dashed line indicates the intersection point of the density plot, and the derived cutoff value is given adjacent to the dashed line. The reliability of single-property models based on each of the three descriptors for the differentiation of oral and parenteral drugs in the training set is given by the Cohen’s kappa (κ) value.

It is of great interest to have simple yet efficient quantitative structure–property relationship (QSPR) models as filters in the selection of compounds in the early phases of drug discovery. This is particularly important for macrocycles and other classes of compounds in the bRo5 space which often require lengthy synthetic routes with low overall yields. With the aim of deriving models that are easy to interpret, we defined the intersection point between orals and parenterals in the density plots for each of the 10 descriptors calculated for the approved macrocycles (Figure S6). For continuous descriptors, the intersection point was used as the cutoff between orals and parenterals, whereas a rounded off value was used for discrete descriptors (HBD, HBA, NRotB, Figure S6). The cutoffs for HBD and TPSA performed best in discriminating oral from parenteral macrocycles, whereas other descriptors such as the NRotB were less useful (Figure 8). However, even though cutoffs for HBD and TPSA were able to define the oral chemical space of the FDA-approved macrocycles with high sensitivity (88% and 92%, respectively), their specificity in the classification of parenteral macrocycles was lower (71% and 66%, respectively, Table S5). This conclusion was also valid when the two descriptors were applied to an external test set of macrocycles that were in clinical trials in 2014 (*n* = 60).^3,14^ Consequently, we proceeded to investigate if models based on two descriptors were able to perform better.

**Figure 9:**
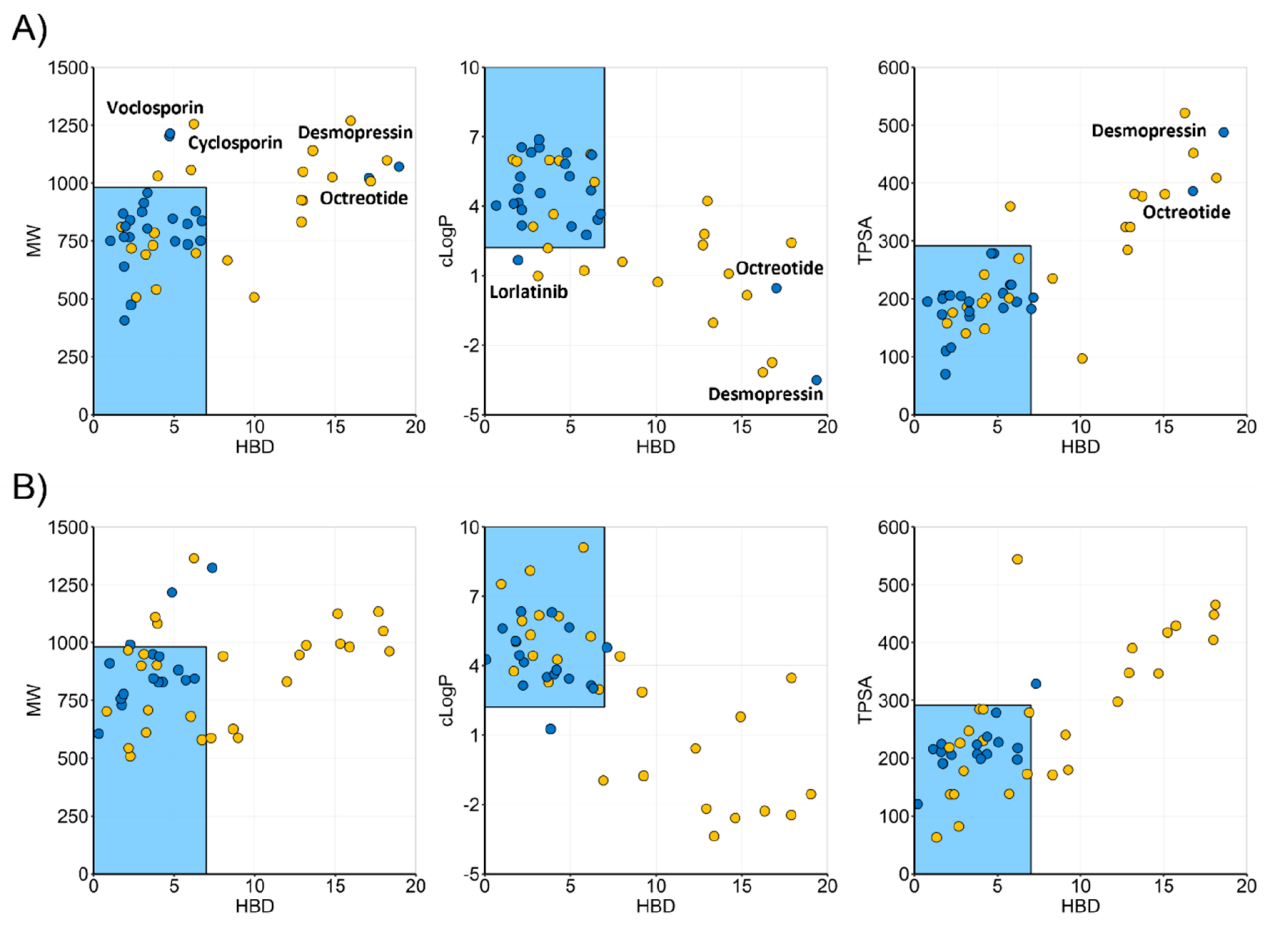
Discrimination of orally bioavailable and parenterally administered (A) macrocyclic drugs and (B) an external test set of macrocycles not yet approved as drugs in bi-descriptor chemical space. Oral drugs are indicated by blue circles, while parenterals are in yellow. The filled circles have been jittered slightly to avoid overlap. The blue shading marks chemical space defined by HBD ≤ 7 and one of MW < 982 Da, cLogP > 2.22, or TPSA < 292 Å^2^. Some parenteral macrocycles are not included in the figures which have been truncated at HBD < 20, MW < 1500 Da, −5 < cLogP < 10, and TPSA < 600 Å^2^. Figure S7 includes all parenterals.

Since cutoffs for MW and cLogP discriminated oral and parenteral macrocyclic drugs almost as well as HBD and TPSA (Table S5), we investigated bi-descriptor models based on all combinations of these four descriptors (Table S6). We found that models based on HBD in combination with cutoffs for any of the three other descriptors (MW, cLogP, TPSA) provided the best discrimination between oral and parenteral macrocycles in the set of FDA-approved drugs (Figure 9A, Table 1, Figure S7, Table S6). Oral macrocycles were predicted with 83–92% sensitivity, i.e., similar to when only HBD or TPSA was used, while parenterals were discriminated with 74–79% specificity, which is an improvement as compared to HBD or TPSA alone. At first glance it appears surprising that two descriptors of polarity, i.e., HBD and TPSA, give models comparable to HBD in combination with either MW or cLogP. However, this may be understood from previous observations that MW and TPSA are correlated for macrocycles and other drugs in the bRo5 space.^3,14^ The cyclic peptides desmopressin and octreotide, which have very low oral bioavailability, constitute the only oral drugs that are misclassified by all three models (Figure 9A). Use of the three bi-descriptor models for prediction of oral and parenteral macrocycles in the external test set gave satisfactory predictions (83–94% sensitivity, 67–71% specificity, Figure 9B, Table 1). After rounding off some of the cutoffs, this analysis found that HBD ≤ 7 in combination with either of MW < 1000 Da, cLogP > 2.5, or TPSA < 300 Å^2^ are guidelines that are easy to memorize and can be used in the design of orally available macrocycles. Beyond these limits, the probability of discovering an oral macrocyclic drug is very low, but parenteral drugs can be located within the oral space of the guidelines.

**Table 1:** Most Accurate Bi-descriptor Models for Prediction of Oral Bioavailability for Macrocycles^a^

			confusion matrix							
bi-property models	1st cutoff	2nd cutoff	TP	TN	FP	FN	sensitivity	specificity	accuracy	κ
training set (*n* = 62)	HBD (≤7)	MW (<982 Da)	20	30	8	4	0.83	0.79	0.81	0.6
		cLogP (>2.22)	21	30	8	3	0.88	0.79	0.82	0.64
		TPSA (<292 Å^2^)	22	28	10	2	0.92	0.74	0.81	0.62
test set (*n* = 60)	HBD (≤7)	MW (<982 Da)	15	30	12	3	0.83	0.71	0.75	0.48
		cLogP (>2.22)	17	28	14	1	0.94	0.67	0.75	0.51
		TPSA (<292 Å^2^)	17	28	14	1	0.94	0.67	0.75	0.51

a Cutoffs were selected based on the major intersection between orals and parenterals in the density plots for each descriptor as calculated for the approved macrocycles (Figure 8). Abbreviations: true positive (TP), true negative (TN), false positive (FP), false negative (FN), and κ coefficient. Positive values stand for “oral”.

#### Model Benchmarking against the AB-MPS Score

AbbVie’s multiparameter scoring function (AB-MPS) [AB MPS = Abs(cLogD-3) + NAR + NRotB] is useful for the prediction of oral bioavailability of compounds in the bRo5 space.^4^ An AB-MPS score of ≤14 was found to be a cutoff between acceptable (*F* > 27.3%) and low–moderate oral bioavailability in the bRo5 space, while an AB-MPS score of ≤15 could be used to differentiate between orally bioavailable and parenterally administered drugs. We found that the AB-MPS ≤ 15 cutoff classified most of the macrocycles in our training and test sets correctly as being orals or parenterals (Figure S8). The oral macrocycles in the two drug sets were correctly predicted with 79% and 61% sensitivity, respectively, while parenterals were discriminated with 71% and 79% specificity (Table S7). The three HBD biproperty models presented above performed somewhat better, i.e., orals were predicted with 83–94% sensitivity and parenterals discriminated with 67–79% specificity (Table S6).

#### Differentiating between HBDs

**Figure 10:**
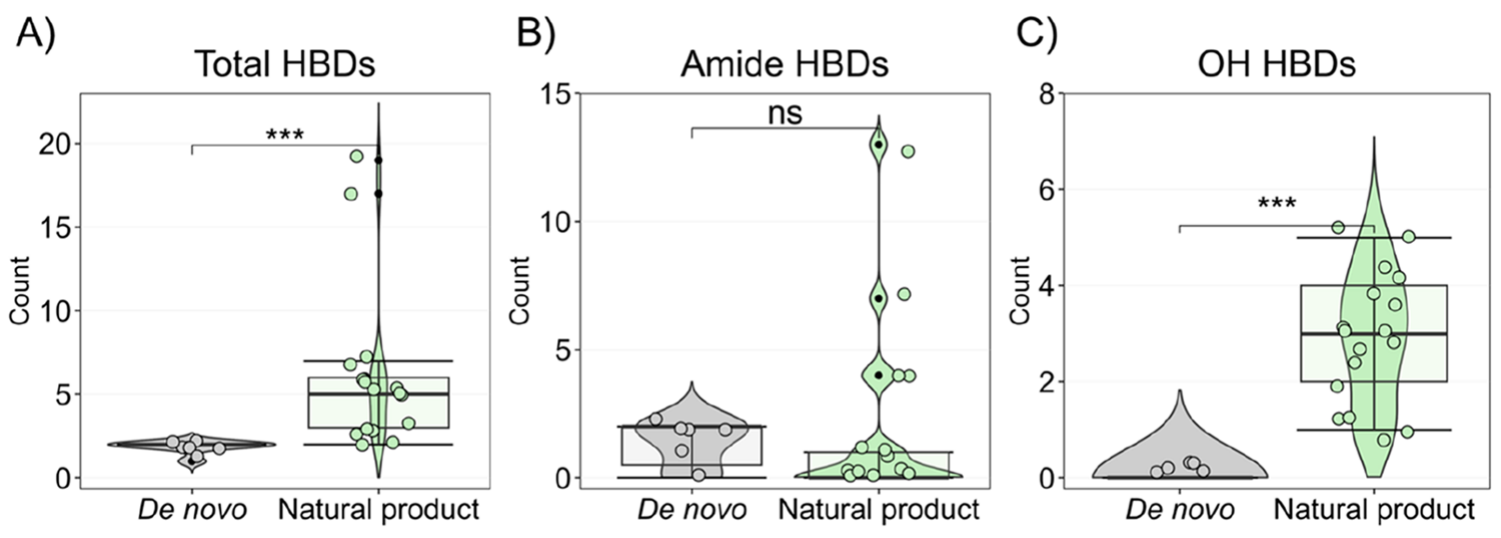
(A) Comparison of the number of HBDs in orally bioavailable macrocyclic drugs discovered by de novo design (*n* = 7) or from natural products (*n* = 17) for the charge state calculated at pH 7.0. Frequencies of HBDs originating from (B) amide moieties and (C) phenols and aliphatic alcohols (OH) in the two classes of drugs. The natural product class includes both original natural products and semisynthetic derivatives. Box plots show the 50th percentiles as horizontal bars, the 25th and 75th percentiles as boxes, and the 25th percentile minus 1.5× the interquartile range and the 75th percentile plus 1.5× the interquartile range as whiskers. Black dots represent values higher than 1.5× the interquartile range and less than 3× the interquartile range at either end of the box. Violin shapes represent the data density at each count value.

As restriction of the number of HBDs to ≤7 was found to be essential for oral bioavailability, we investigated what kinds of HBDs^47^ are found in the oral macrocycles to support future design and optimization of macrocyclic drugs. Splitting of the set of oral macrocyclic drugs based on their origin, i.e., if they had been designed de novo or were natural products or derivatives thereof, also provided useful insight (Figure 10). Natural product-based macrocycles contain a significantly higher number of HBDs than the de novo-designed drugs (Figure 10A). The de novo class only has one or two HBDs per macrocycle, which mainly originate from amide bonds (Figure 10B). In contrast, HBDs in natural products overwhelmingly originate from phenols and aliphatic alcohols, as highlighted earlier in studies of bioactive natural products (Figure 10C).^48,49^ Cyclosporin and voclosporin constitute exceptions since each has four HBDs originating from amide bonds. However, it is generally assumed that chameleonicity, i.e., the involvement of these HBDs in intramolecular hydrogen bonds, is essential for their oral bioavailability.^26^ Our analysis thus emphasizes that the number of amide-type HBDs of macrocycles preferably should be kept at ≤2 for satisfactory oral bioavailability. Caution should therefore be exercised when incorporating amides, sulfonamides, and related functional groups in the de novo design of macrocycles. No significant difference was found between natural products and de novo-designed macrocycles for the number of HBDs of nitrogen-containing heterocycles, whereas the natural products had a somewhat higher frequency of HBDs from protonated bases, i.e., aliphatic amines and guanidines (Figure S9). Analysis of the HBDs for the neutral forms of the oral macrocycles, compared to the pH 7.0 state, led to almost identical conclusions (Figure S10). The main difference was that the neutral form of the de novo-designed HCV NS3/4A protease inhibitors contains a HBD originating from the acyl sulfonamide moiety, a functionality which does not occur in the natural product-derived macrocyclic drugs.

### Macrocycles in Clinical Trials

The FDA-approved macrocyclic drugs provide a historical view of macrocyclic drug discovery. To investigate current approaches and macrocycles that could be approaching the market, clinical candidates were extracted from Drugbank’s “investigational” class and examined for their clinical trial status in the United States.^50^ Only macrocycles that had completed clinical trials since 2017 or that are currently in clinical trials were selected to reduce the likelihood of including compounds that had failed in the data set. In spite of that, analysis of compounds in clinical studies is always associated with some uncertainty, e.g., as therapeutic indications and routes of administration may change before a drug is approved. Out of the resulting 34 macrocyclic clinical candidates (Table S8), 11 were dosed orally for systemic distribution (32%), whereas 23 were administered parenterally. However, the proportion of orals may be underestimated as drugs are often administered parenterally in phase I studies even though they may be intended for oral dosing in phase II and III. In spite of the uncertainty about the number of orally dosed clinical candidates, it seems that medicinal chemists have not been able to increase their proportion as compared to the macrocyclic drugs already approved by the FDA (39% orals). Six macrocycles were classified as de novo designed (18%) and 28 as natural products. Interestingly, and perhaps surprisingly, the proportion of natural product derived macrocyclic clinical candidates (82%) is almost identical with that of the FDA-approved drug set (88%). It thus appears that approaches for de novo design of macrocycles have not yet had a major influence on the clinical pipeline. Subdivision of the natural products, as done for the macrocyclic drugs, showed that the majority were natural product derivatives (*n* = 24) while only four were originally natural products (*n* = 4, Table S9). For seven of the natural product derivatives, the original natural product or another derivative had already been approved as a drug. Interestingly, three of the natural product derivatives were cyclic peptides or mimetics thereof obtained from screening of cyclic peptide libraries or from phage display. The three are all parenterals in phase I or II clinical studies and may indicate that the recent high interest in cyclic peptides^51^ is beginning to deliver into the clinic. Information on the rationale for producing the natural product derivatives was only found for nine of them and included both pharmacokinetics and pharmacodynamics, with pharmacokinetics being somewhat more frequent.

#### Therapeutic Indications and Targets

**Figure 11:**
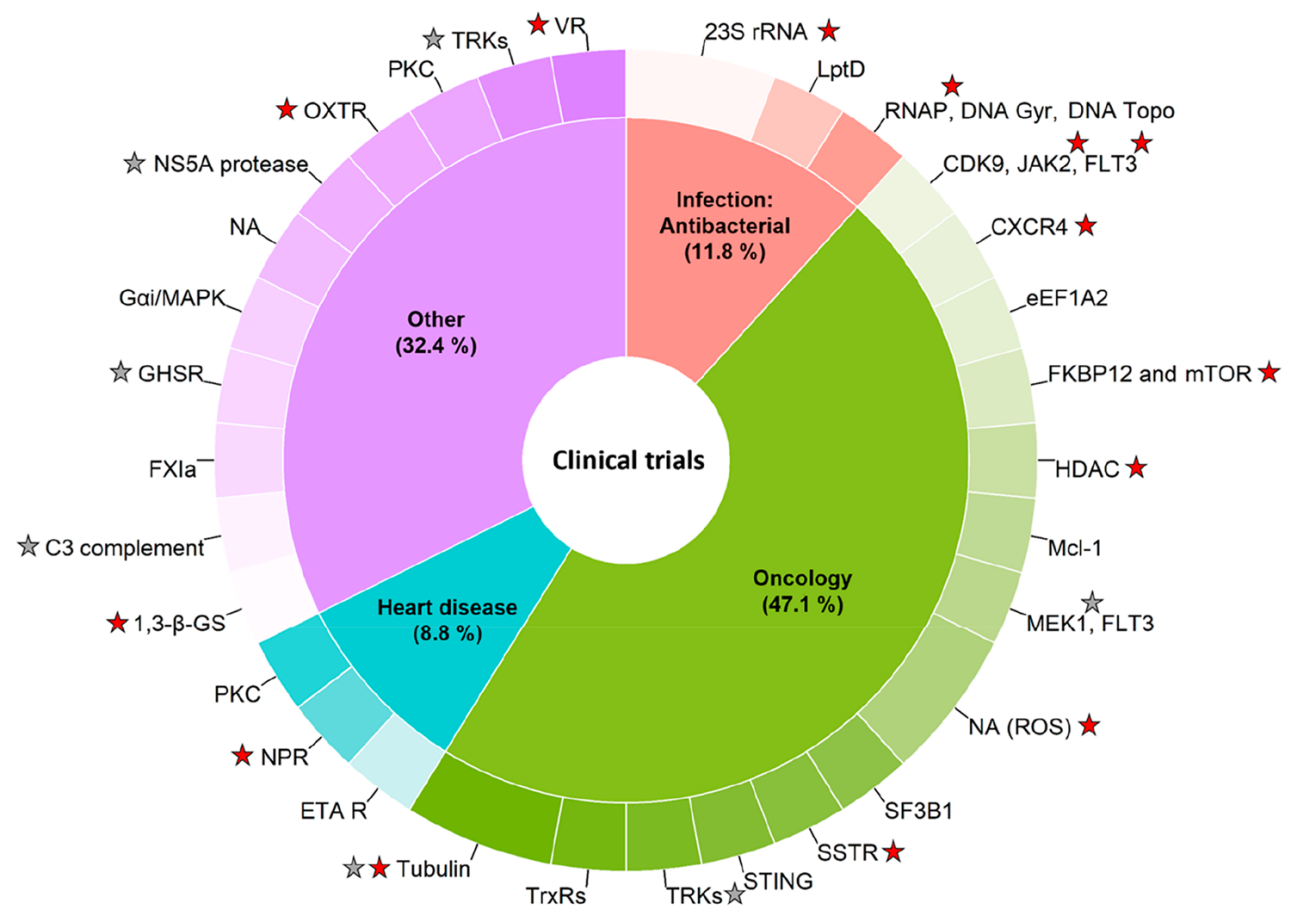
Therapeutic indications (inner circle) and targets (outer circle) for the macrocycles in clinical trials (*n* = 34). Therapeutic indications treated by only one clinical candidate (each 2.9%) have been grouped under “Other”. Targets with FDA-approved macrocyclic drugs have been marked with a red star, while a gray star indicates that the target is modulated by an approved nonmacrocyclic drug. Targets separated by “and” indicate that the corresponding drug is a molecular glue, while targets separated by a comma indicate that the corresponding drug displays polypharmacology. NA: Target not available. A complete list of therapeutic indications and targets for the macrocycles is clinical trials is available in Table S8.

The therapeutic indications of the clinical candidates revealed several differences compared to the approved drugs (Figure 11, Table S8). Antibacterial agents are a major indication among the drugs (30%) but has now fallen behind oncology, which has taken over as the major indication for the clinical candidates (47%). Heart disease has emerged as a significant indication, and macrocyclic clinical candidates are now also being investigated in several other new indications, ranging from antithrombotic to septic shock (cf. “Other” in Figure 11 and Table S8 for a complete list). It is interesting to note that antifungals and antivirals, indications which include several successful macrocyclic drugs, each only have one compound in clinical trials (Table S8). For antivirals, this most likely originates from the success of the marketed drugs for treatment of HIV and HCV infections which has reduced the medical need for additional drugs.

The 34 macrocyclic clinical candidates are directed toward an approximately equal number of targets, with oncology being the most target-rich indication (Figure 11). To gain insight into the extent to which the macrocyclic clinical candidates may become “first-in-class” small-molecule drugs for their respective targets, we retrieved information on what macrocyclic and nonmacrocyclic drugs are already approved for each target from the ChEMBL database.^58^ Biological drugs, i.e., proteins and antibodies, were excluded, and clinical candidates for the targets were not included due to the difficulty in judging their likelihood of being approved. Then, FDA-approved macrocyclic drugs in the data set compiled in this manuscript that were not present in ChEMBL were added (Table S10). We found that clinical candidates in oncology were often aimed at targets validated by approved macrocyclic drugs (i.e., CXCR4, FKBP12, FLT3, HDAC, JAK2, mTOR, ROS, SSTR, and tubulin) and that nonmacrocyclic drugs had been approved for some of the targets. In addition, the clinical candidates in oncology were directed toward several novel targets, for which neither macrocyclic nor nonmacrocyclic drugs have been approved. Three of the antibacterial clinical candidates target protein and RNA synthesis, just as approved macrocyclic drugs, while one candidate is directed to a novel target, lipopolysaccharide transport protein D (LptD), which is responsible for lipopolysaccharide biogenesis in the membrane. In heart disease, macrocycles are directed toward three different targets, one of which (natriuretic peptide receptor, NPR) is modulated by an existing macrocyclic drug. The targets of the remaining 11 “other indications” were split into three approximately equal groups, i.e., those not modulated by any existing drug and those modulated by either a macrocyclic or a nonmacrocyclic drug. Even though this analysis is limited by the exclusion of clinical candidates, it tentatively indicates that a major part of the targets of the clinical candidates could be more suited to modulation by macrocyclic than by nonmacrocyclic drugs. However, a minor but still significant number of the targets are modulated by nonmacrocyclic drugs.

#### Binding Site and Ligand Shape

Drug–target crystal structures have only been reported for five of the clinical candidates (Table S11), which presumably reflects the novelty of many of them (15 out of 34 are in phase I or I–II clinical studies). Three of the five bind to targets that have a groove-shaped binding site, one in a tunnel, and one in a pocket. Analysis of the shapes of the target-bound macrocycles reveals that the five adopt conformations that are more disc- and sphere-like than Ro5-compliant drugs (Figure S11), just as for the approved macrocyclic drugs.

#### Chemical Space

**Figure 12:**
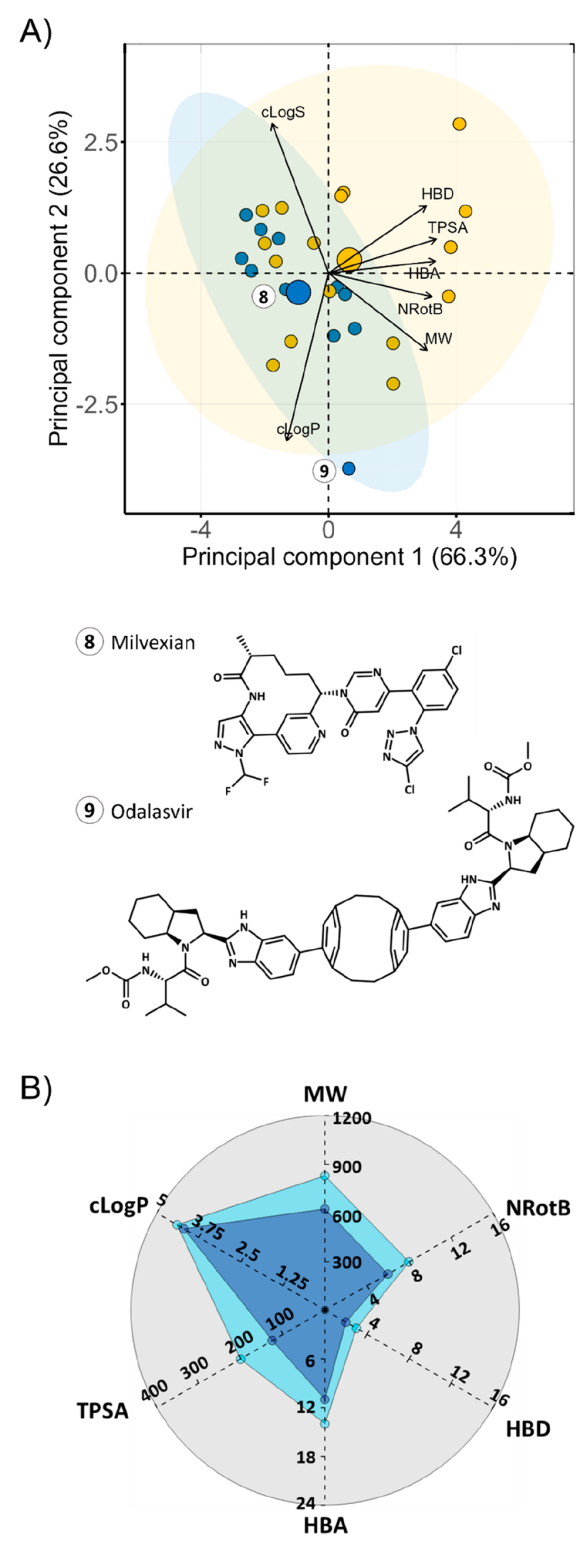
(A) Principal component analysis of the chemical space of the clinical trials data set (*n* = 27). The PCA was based on the descriptors of Lipinski’s^41^ and Veber’s^42^ rules as well as cLogS calculated at pH 7.0. Five parenterals with MW > 1500 Da were excluded from the PCA to provide a better dissection of the chemical space of the orally bioavailable macrocycles [cf. Figure S13 for the PCA for the complete set of macrocycles (*n* = 32)]. Ellipses in blue and yellow shading show the 95% confidence intervals for orally and parenterally administered macrocycles, respectively. The centroid of each class is indicated with a large circle in the color of the respective class. The contributions of individual descriptors to the PCA are indicated by the length of the arrows. The structure of the oral outlier odalasvir (**9**) is provided. The structure of milvexian (**8**), which is close to the centroid of the oral class, is given for comparison. Avasopasem manganese and motexafin gadolinium were removed due to calculation errors with metals. (B) Radar plot comparing the median values for the descriptors employed in Lipinski’s Ro5 and Veber’s rule for the oral FDA-approved (light blue, *n* = 24) and clinical trial macrocyclic subsets (dark blue, *n* = 11). Note that HBD, HBA, and TPSA were calculated differently than in the original rules (cf. Methods).

Just as for the macrocyclic drugs, orally and parenterally administered macrocyclic clinical candidates showed some separation in chemical space, with the orals showing the expected distribution toward higher lipophilicity in combination with lower MW, polarity, and flexibility (Figure 12A, Figures S12 and S13, Table S12). In contrast to the approved drugs, for which the orals were found in four distinct regions of chemical space, the clinical candidates were located in one region with odalasvir as an outlier. In addition, the oral clinical candidates are found closer to the Ro5 chemical space than the orally bioavailable macrocyclic drugs (Figure 12B, Figure S13). Median values for MW, polarity (TPSA, HBA, and HBD), as well as flexibility (NRotB) are all somewhat lower for the clinical candidates than for the approved drugs, while the lipophilicity is almost identical for the two sets of compounds. This observation is unlikely to be affected if some of the parenterally administered phase I candidates are intended for oral dosing, since they all have a MW at or below the median MW of the oral clinical candidates.

Even though the clinical trial data set is small with only 11 being administered orally, we used it as a second test set to evaluate the performance of the three bi-descriptor models (Table 1) for discrimination of oral and parenteral administration (Table S13). For this test set, the model based on HBD ≤ 7 and cLogP > 2.22 performed better than the two other models (Table S13). Oral macrocycles were predicted with 91% sensitivity, while parenterals were discriminated with 67% specificity (75% accuracy), which is essentially identical with the prediction for the first test set. This outcome provides additional support for simple, bi-descriptor models being be used as a first filter in the design of macrocycles intended for oral absorption.

Investigation of HBD types for the de novo-designed and natural product-derived, orally administered macrocycles in clinical trials revealed trends similar to the approved macrocycles (Figure S14, Figure 10). Despite the difference in the total number of HBDs between the two classes not being statistically significant, there is a trend toward natural products having more HBDs (Figure S14A). Moreover, de novo-designed macrocycles in clinical trials have a significantly higher number of amide-type HBDs but fewer hydroxyl-type HBDs (Figure S14B and S14C). No differences were found for aromatic amines and aliphatic amines protonated at pH 7.0 (Figure S14D and S14E). Just as for the macrocyclic drugs, this highlights that the synthesis of de novo-designed macrocycles relies on the formation of amide bonds, which occur less frequently in natural products. In addition, it is noticeable that the de novo-designed clinical candidates lack hydroxyl groups.

### Future Trends in Macrocyclic Drug Discovery

The therapeutic indications and targets of the macrocyclic drugs and clinical candidates provide a perspective of macrocyclic drug discovery that ranges from the fist approval of cyanocobalamin for vitamin B12 deficiency in 1942 to the ongoing clinical studies (Figures 3 and 11, Figure S1, and Tables S1 and S8). To get an overview which also includes the future, we mined the recent literature in medicinal chemistry, i.e., from 2005 to the mid-2022. All articles in 20 leading journals in medicinal chemistry that had “macrocycle” as a keyword were examined manually, resulting in the collection of 509 articles reporting macrocycles for a therapeutic indication and/or target.

#### Indications

**Figure 13:**
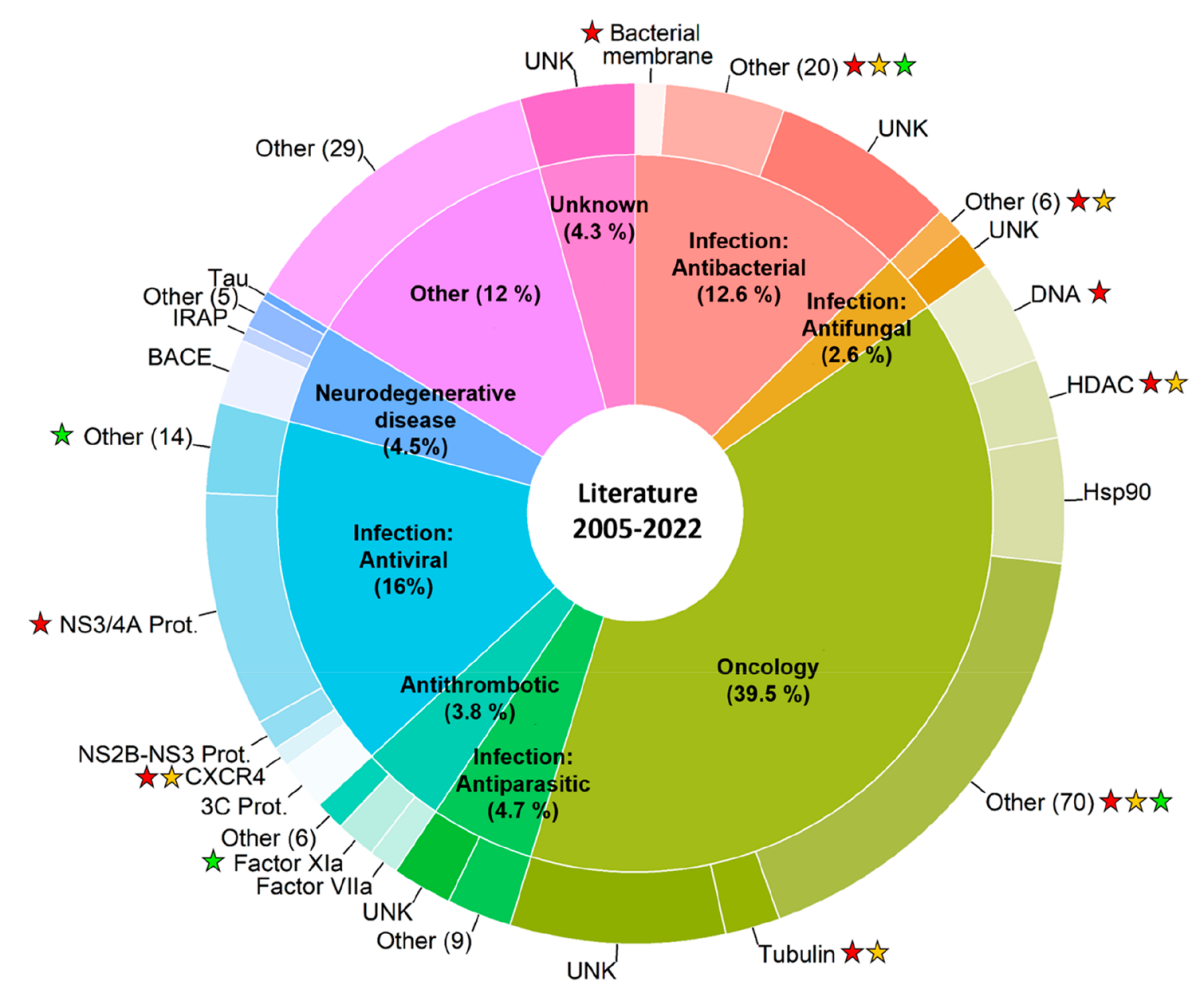
Therapeutic indications (inner circle) and targets (outer circle) of the macrocycles reported in the articles published in 20 leading medicinal chemistry journals during 2005–2022 (*n* = 532). Therapeutic indications amounting to <2% of the entries have been grouped under “Other”. Less explored targets for each indication have been clustered as “Other”, with the number of unique targets provided in brackets. UNK denotes that the target is unknown or not reported. Targets with FDA-approved macrocyclic drugs have been marked with a red star, while an orange star indicates one or several clinical candidates toward the same target. A green star indicates that a clinical candidate is directed toward a novel target, i.e., a target not modulated by an existing drug. A star adjacent to “Other” may indicate one or several drugs or clinical candidates directed toward one or several targets (cf. Tables S1 and S8 for full details). Complete lists of articles reporting therapeutic indications and/or targets retrieved from the literature are provided as .csv files in the Supporting Information.

A comparison of the therapeutic indications for the macrocycles reported in the recent literature to the approved macrocyclic drugs (Figure 3, Table S1, Figure S1) and clinical candidates (Figure 11 and Table S8) revealed several trends (Figures 13, Figures S15 and S16). Oncology is the most abundant indication in the recent literature (39.5%), with antivirals (16%) and antibacterials (12.6%) in second and third place, respectively. Similarly, oncology (47.1%) was the most frequently studied indication for the clinical candidates but with antibacterials (12%) in second place. In contrast, antibacterials (29.6%) dominated the set of approved macrocyclic drugs with oncology (21.1%) as the second indication and antifungals (8.5%) as the third most frequent indication. Most likely, these changes reflect the declining interest in the development of novel antibiotics and antifungals in combination with an increased emphasis on oncology in the pharmaceutical industry during the recent decades.^52−54^ Autoimmune diseases and immunosuppressants, which are minor but still significant indications for the approved drugs, have also received significantly less attention during recent years (Figures S15 and S16). The decline for antibacterials, antifungals, immunosuppressants, and treatments for autoimmune diseases may also originate from the shift away from natural products toward hits from HTS as sources for drugs that has taken place since the late 1990s.^55−57^ Interestingly, the recent literature reveals that macrocycles are now also being investigated in a wealth of indications previously unexplored by macrocycles. For instance, about 4% of the macrocycle literature is focused on neurogenerative disease and on thrombosis (Figure 13). New indications for macrocycles also include analgesia, angiogenesis, cardiovascular disease, and inflammation (grouped under “Other” in Figure 13).

#### Targets

A large proportion of the recent literature concerns the NS3/4A protease of the hepatitis C virus (Figure 13, Figure S17). As mentioned above, this reflects the intense efforts to find a cure for HCV that resulted in the approval of five macrocyclic drugs since 2013 (Table S1). However, other HCV targets such as the NS2B-NS3 protease have also been investigated, while the 3C protease has been studied for other viral infections. In oncology, drugs that act on DNA, HDAC, tubulin, and several other targets (ALK, CXCR4, mTOR, SSTR2, JAK2, and FLT3) have been approved, while novel macrocycles are currently being evaluated in the clinic against some of these targets (HDAC, tubulin, CXCR4, mTOR, and SSTR2). In addition, macrocycles have been reported in studies of a large number of other oncology targets (Figure 13). Some of these have progressed into clinical trials, for example, macrocycles targeting CDK9, mitogen-activated protein kinase kinase (MEK), induced myeloid leukemia cell differentiation protein (Mcl-1), stimulator of interferon genes (STING), and tropomyosin receptor kinase (TRKs) (Table S8, Figure S17). In the field of antibacterials, the bacterial rRNA complex and RNA polymerase are inhibited by several macrocyclic drugs while also being the target of novel macrocycles in the clinic. Moreover, macrocycles have been reported in studies of a rather large number of novel antibacterial targets, many of which have not been disclosed or are not yet known. Macrocycles are also being explored as inhibitors of coagulation factors FXIa and FVIIa as treatments for thrombosis, with the FXIa inhibitor milvexian having completed phase II trials. For neurodegenerative disease, BACE-1 has been investigated intensely as a treatment for Alzheimer’s disease, so far without success.

#### Chemical Space

**Figure 14:**
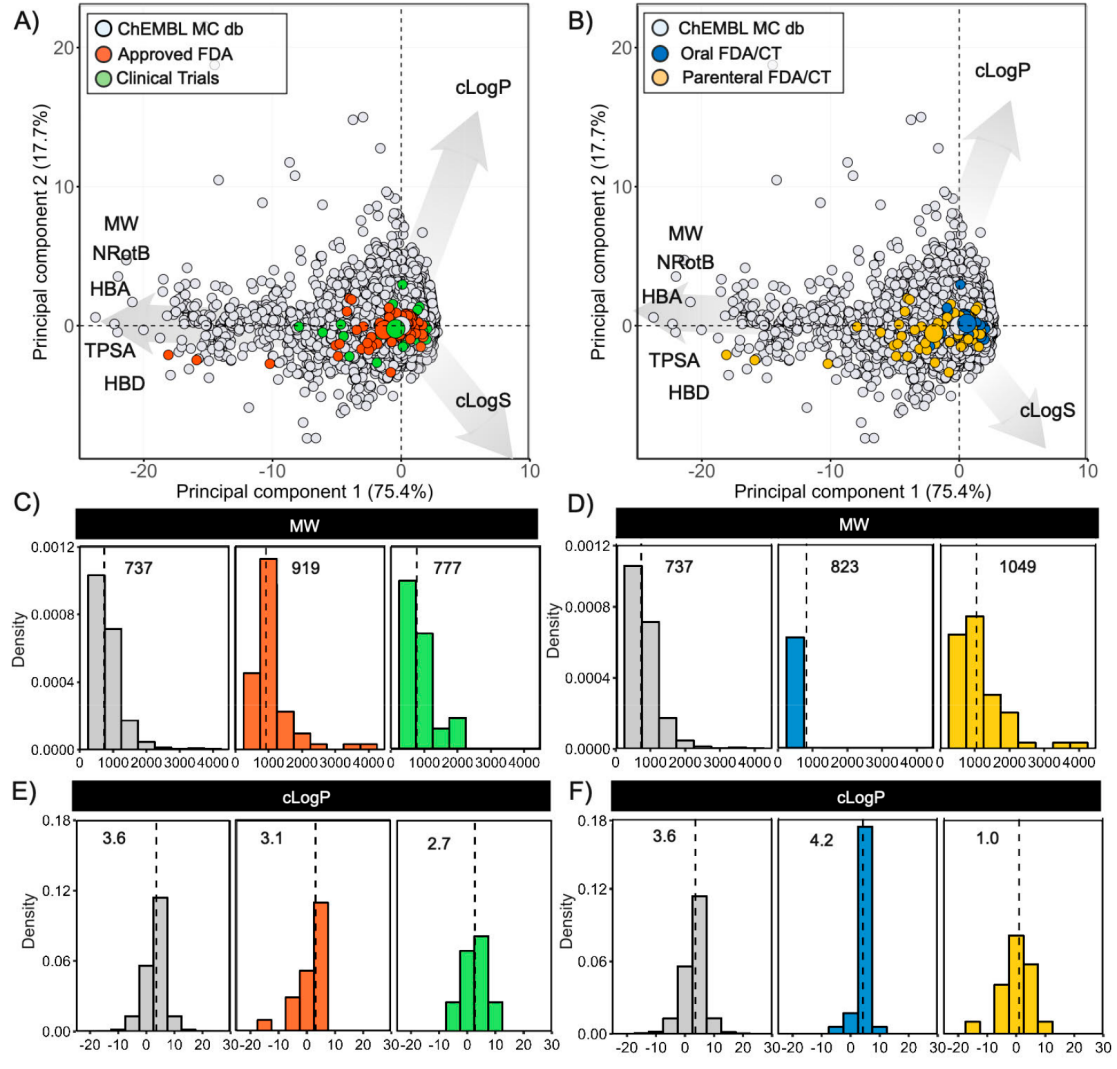
Principal component analysis comparing the chemical space of the macrocycles retrieved from ChEMBL (*n* = 28052, in gray) to (A) the macrocyclic drugs approved by the FDA (*n* = 62, in red) and the macrocycles (MC) undergoing clinical trials (CT) (*n* = 32, in green) and to (B) the combined oral (*n* = 35, in blue) and parenteral (*n* = 59, in yellow) parts of the drugs and clinical candidates data sets. The centroid of each class is indicated with a large circle in the color of the respective class. The PCA was based on the descriptors of Lipinski’s^41^ and Veber’s^42^ rules as well as cLogS, calculated at pH 7.0. The contributions of individual descriptors to the PCAs are indicated by the length of the arrows. PCAs for macrocycles with MW < 1500 Da are found in Figure S18. (C and E) Distribution of molecular weight (MW) and calculated lipophilicity (cLogP) for the macrocycles in the ChEMBL (*n* = 28 052, in gray), drug (*n* = 62, in red), and clinical candidates (*n* = 32, in green) data sets. (D and F) Distribution of molecular weight (MW) and calculated lipophilicity (cLogP) for the macrocycles in the ChEMBL data set (*n* = 28 052, in gray) and in the combined oral (*n* = 35, in blue) and parenteral (*n* = 59, in yellow) parts of the drugs and clinical candidate data sets. The median value for the descriptor is given in each panel and indicated by a dashed line.

The regions of the chemical space populated by macrocycles approved as drugs or in clinical trials represent just over 90 compounds which constitute the successful “tip of the iceberg” of macrocyclic drug discovery. To get a more comprehensive overview of the chemical space in which macrocycles are investigated for bioactivity, we extracted all macrocycles from the ChEMBL database (*n* = 28 052).^58^ As might be expected, principal component analyses revealed that macrocycles investigated for bioactivity populate a chemical space that is much larger than that of the drugs and clinical candidates, which occupy a more confined region of chemical space (Figures 14A). As already noted, orally bioavailable drugs and clinical candidates are somewhat smaller, more lipophilic, and less flexible than the parenteral ones (Figures 14B, 7, and 12). The future will tell to what extent macrocycles in molecular property regions beyond those already explored will be able to advance into the clinic and onto the market.

Comparison of the distribution of the seven property descriptors used in PCA and two descriptors of chemical complexity, i.e., the macrocycle ring size and the number of stereocenters, provided an alternative perspective of the chemical space of the different sets of macrocycles (Figure 14C–F, Figures S19 and S20). Even though the macrocycles in the ChEMBL set explore wider ranges for the property descriptors than the macrocyclic drugs and clinical candidate data sets, the median values do not show major differences between the three sets as illustrated by the size (MW, Figure 14C) and lipophilicity (cLogP, Figure 14E). However, the macrocycles in the ChEMBL set are somewhat smaller, more lipophilic, less polar (TPSA and HBA), and less flexible (NRotB) than the drugs and clinical candidates. The ring size distributions are similar for the three macrocycle sets, but the number of stereocenters is skewed toward the lower end for the ChEMBL set (Figure S19). In fact, the macrocycles in the set of approved drugs have twice as many stereocenters as those in the ChEMBL set. The difference in stereocenters most likely originates from the macrocyclic drug data set having a high proportion of natural products and derivatives thereof, while the ChEMBL set mainly consists of macrocycles designed de novo by medicinal chemists.

Combination of the FDA-approved drugs and clinical trial data sets followed by subdivision into an oral and a parenteral set revealed striking similarities between the oral set and the ChEMBL set for all seven property descriptors (Figure 14D and 14F, Figure S20). Parenteral macrocycles are larger, less lipophilic, more polar, and more flexible. The ChEMBL and oral sets have somewhat smaller macrocyclic rings than the parenteral macrocycles, while the ChEMBL set again stands out by its low number of stereocenters. In spite of this difference, the majority of the macrocycles in the ChEMBL set are located in chemical space that could well provide novel macrocyclic drugs in the future.

## Summary, Conclusions, and Perspectives

### Summary and Conclusions

We have created a database consisting of 67 macrocyclic drugs approved by the FDA to get an up-to-date view on what macrocyclic drugs are used for and in what chemical space they reside. Three additional databases, one listing 34 macrocycles in clinical studies in the United States, another consisting of 509 articles on studies of macrocycles in drug discovery published since 2005, and a third composed of the macrocycles reported in ChEMBL (*n* = 28 052), provide different views of what the future of macrocyclic drug discovery might look like. The most important findings from the analysis of the four databases are as follows.

- Macrocyclic drugs are mainly used as antibacterials with oncology as the second most important indication, while the order between the two indications is reversed for the clinical candidates. The literature survey indicates that oncology will remain the major indication in the future but that macrocycles also are positioned to be used in a multitude of novel indications. In addition, the analysis highlights that macrocycles are in clinical trials or studied preclinically against a large number of different targets not modulated by current macrocyclic or nonmacrocyclic drugs.
- Inspection of the structures of target-bound complexes of macrocyclic drugs (*n* = 34) reveal that 79% of them modulate targets that have flat, tunnel-shaped, or groove-shaped binding sites. It is important to note that ternary complexes, in which the macrocycle acts as a molecular glue, account for three of the groove-shaped binding sites. Comparison with a reference set of Ro5-compliant drugs concluded that macrocycles can modulate these difficult to drug targets because macrocycles are more likely to adopt disc- and sphere-like conformations. Target-bound crystal structures have only been reported for five of the clinical candidates. Three of these macrocycles bound in groove-shaped binding sites and one in a tunnel. Interestingly, an analysis of the competitive landscape for the targets of the clinical candidates tentatively indicated that a major part of these targets may be more suited to modulation by macrocyclic than by nonmacrocyclic drugs.
- Natural products and derivatives thereof dominate over de novo-designed compounds (ratio > 4:1), among both the macrocyclic drugs and the clinical candidates. Subdivision of the drugs into original natural products and natural product derivatives revealed that the derivatives had been made to improve the pharmacokinetics, pharmacodynamics, or both of them in a few cases. However, the improvements did not result in any significant difference in the chemical space populated by the two subclasses of natural products. Three cyclic peptides obtained from screening of peptide libraries or from phage display were found among the natural product derivatives of the clinical candidates data set. This may indicate that the recent interest in cyclic peptides is beginning to deliver into the clinical pipeline. On average, the macrocycles in the ChEMBL data set contain only one-half as many stereocenters as those in the drugs set and thus appear more de novo like than the drugs.
- Close to 40% of all macrocyclic drugs are orally bioavailable, while just over 30% of the clinical candidates appear to be orally bioavailable. Orally and parenterally administered macrocyclic drugs and clinical candidates populate partially overlapping regions of chemical space. Interestingly, simple bi-descriptor models, i.e., HBD ≤ 7 in combination with either MW < 1000 Da or cLogP > 2.5, distinguished orals from parenterals with approximately 90% sensitivity and 70% specificity. We propose that these simple guidelines can be used as a first filter to assess whether a macrocyclic lead is likely to be orally bioavailable or not and again highlight that the HBD count is based on the major charge state predicted at pH 7.0. We emphasize that if HBDs close to the upper guideline of 7 are to be incorporated in an orally bioavailable drug, the majority should be aliphatic alcohols or phenols. Alternatively, the compound should be designed to form intramolecular hydrogen bonds, thereby reducing its effective HBD count by behaving as a molecular chameleon. Secondary amide bonds are commonly used in medicinal chemistry, but more than two such amide bonds occur only rarely in oral macrocyclic drugs and clinical candidates.

### Perspectives

Guidelines that define the chemical space of orally bioavailable macrocycles, such as those we presented above, are useful as a starting point for design in macrocycle drug projects. Computational methods which predict the biologically relevant conformations of macrocycles, e.g., those adopted in aqueous solution, when crossing a cell membrane, and when bound to the drug target, would then be of enormous value to guide more precise design efforts. However, conformational analysis of macrocycles is challenging since macrocycles often display a significant flexibility which is influenced by ring strain, intramolecular hydrogen bonds, and other transannular interactions.^59^ Attached side chains further increase the complexity of defining macrocycle conformational ensembles.^24^ Last but not least, dynamic intramolecular hydrogen bonds, shielding of polar groups by aromatic groups (e.g., NH−π interactions), and other intramolecular interactions may allow macrocycles to behave as molecular chameleons that adapt their conformations to the surrounding environment.^21,22,24,25,60^

It has been found that the conformational space of macrocycles is sampled well by different algorithms, allowing the ensembles to be used for docking into potential drug targets.^11,61,62^ However, even though conformational ensembles of macrocycles contain biologically relevant conformations, their prospective identification by methods that use molecular mechanics force fields has been found to be very difficult.^23,63,64^ For instance, minimum energy conformations (MECs) predicted for macrocyclic drugs and lead-like, natural product-inspired macrocycles showed large differences from target-bound and solution conformations.^23,63^ Encouragingly, progress toward the prediction of solution ensembles in environments that differ in polarity and thereby toward the design of compounds that are soluble and cell permeable has recently been reported for some classes of macrocycles. Molecular dynamics simulations using an explicit solvation model followed by refinement of conformations at the ab initio level predicted the chameleonic behavior of a series of 12-membered macrocycles.^28^ For this series, predicted solvent-dependent intramolecular interactions between side chains and the macrocyclic ring and a solvent-induced conformational switch of the ring were both verified by NMR spectroscopy. The Rosetta generalized kinematic closure method was used to design 6–12-membered cyclic peptides that fold into predicted conformations stabilized by transannular intramolecular hydrogen bonds.^27^ Membrane permeability was achieved for cyclic peptides that adopted stable folds in which all amide NH groups were involved in intramolecular hydrogen bonds or were *N*-methylated. Impressively, cell-permeable cyclic peptides for which the folding varied depending on the polarity of the surrounding medium were also designed. Cis/trans isomerization at an *N*-alkylated amide in the macrocyclic ring was used as a key design element in these molecular chameleons, just as in a study of how the position of the *N*-alkylated amide within a decameric cyclic peptide influenced backbone rigidity and ADME properties.^65^ In addition, the finding that connection of hydrophobic surfaces in cyclic peptides by incorporation of a single N- or C-methyl group can increase membrane permeability significantly may provide a general approach for further property-based optimization of macrocycles.^66^

Artificial intelligence is being used to an increasing extent in drug discovery and provides an alternative to structure- and conformation-based property predictions when large data sets are available.^67,68^ Deep learning and neural networks require very large data sets which are usually not available in drug discovery projects. However, different machine learning methods have been found to be robust enough for building of ADMET models.^69,70^ For instance, models for the cell permeability of macrocycles have been constructed using traditional QSAR methods^22,71^ as well as by machine learning algorithms introduced more recently.^23,72^ Methods for prediction of the solubility of macrocycles have also been investigated, albeit for small data sets.^73^

As illustrated in this Perspective, macrocyclic drugs and clinical candidates predominantly originate from natural products and derivatives thereof. Orally bioavailable macrocyclic natural products are found in a larger and somewhat different chemical space than the oral de novo-designed macrocycles that have been approved as drugs. In general, natural products contain larger proportions of oxygen atoms and stereocenters but fewer nitrogen atoms and aromatic rings than de novo-designed compounds.^48,74^ Differences originate from the fact that natural products are synthesized from a limited number of building blocks but via complex biosynthetic pathways.^75^ Even though synthetic chemists do not have access to the same synthetic toolbox as Nature, taking structural inspiration from natural products appears a logical way to boost macrocyclic drug discovery. Such approaches have already been pioneered by several groups. For example, motifs from natural products rich in stereochemistry have been used to design libraries that were then prepared by diversity-oriented synthesis.^76^ Other approaches involve the combination and fusion of natural product-derived fragments to give pseudonatural products,^77,78^ while a comprehensive mining of natural products provided macrocyclic cores that may be used for in silico screening as ligands for difficult to drug targets.^11^ Approaches such as these three that use inspiration from natural products may also mitigate the major drawback of natural products, i.e., that they often have complex structures which result in low-yielding multistep synthetic routes that make lead optimization difficult.

## Methods

### Generation of the FDA-Approved Macrocyclic Drug Data Set

The approved macrocyclic drug data set was retrieved from the FDA database.^33^ In the data set retrieval process, only drugs having ≥12 heavy atoms in the ring were selected. A cyclodextrin (sugammadex), an antibody–macrocycle conjugate (ado-trastuzumab emtansine), a PEG-linked macrocycle (pegcetacoplan), and contrast agents (^64^Cu–DOTATATE, ^68^Ga–DOTATATE, ^68^Ga–DOTATOC, and gadoterate meglumine) were discarded. The macrocycles in the data set were classified based on their origin, i.e., as “natural products” or as “de novo designed”. Moreover, the “natural products” were subdivided into “original natural products” and “natural product derivatives”. The former category includes compounds directly obtained from a natural process (e.g., erythromycin) or synthetic compounds that are identical with a natural product (e.g., ziconotide). The latter category includes macrocyclic structures obtained in the optimization of an original natural product, porphyrin derivatives, analogues of peptide hormones, and cyclic peptides from synthetic libraries and phage display. The macrocyclic drugs were also classified based on their route of administration (oral or parenteral). Orals were defined as those using the oral administration route and exerting a systemic mechanism of action. Therefore, several antibacterials with local action in the gastrointestinal tract were classified as parenterals. Next, the target, disease indication, and year of first FDA approval of each drug in the data set were manually retrieved from Drugbank and/or specific FDA resources. Molecular property descriptors were calculated for the uncharged structures and at pH 7.0 as described below.

### Generation of the Macrocyclic Clinical Candidate Data Set

Macrocyclic compounds in clinical trials were retrieved and manually curated from DrugBank^34^ and the FDA’s clinical trials Web site (ClinicalTrials.gov).^50^ Briefly, a set of 4098 “investigational” and/or “investigational/experimental” compounds in DrugBank were retrieved and filtered to retain only the macrocycles (≥12 heavy atoms in the macrocyclic ring; *n* = 97). Then, the approval status of each compound in the United States was manually checked in ClinicalTrials.gov, and the most recent clinical trial and its date was recorded with its identity number, indication, and route of administration. To avoid including failed candidates, only macrocycles with finished clinical trials in or after 2017 and ongoing or recruiting clinical trials were selected (*n* = 34). As described in the previous section, clinical candidates were further classified based on their origin (original natural product, natural product derivative, or de novo designed) and route of administration (oral or parenteral; Table S7). However, since ClinicalTrials.gov may not be fully updated regarding the formulation and route of administration for each clinical candidate, oral bioavailability was checked for some candidates. Thus, bryostatin 1 and iso-fludelone/KOSN-1724 were found to be orally bioavailable and were classified as orals despite their clinical trial status. Moreover, nafithromycin was also administered orally in earlier clinical trials (NCT02903836) and was classified as an oral candidate despite the parenteral use in the most recent clinical trial. In addition, the drug target and indication were also curated as for the macrocyclic drugs data set. Molecular property descriptors were calculated for the uncharged structures and at pH 7.0 as described below.

### Generation of the ChEMBL Macrocycle Data Set

The ChEMBL database^58^ was searched for compounds having ≥12 heavy atoms in a ring (macrocycles intended to be bioactive molecules; *n* = 28 053). The simplified molecular input line-entry system (SMILES) codes were retrieved and adapted to pH 7.0; then, molecular property descriptors were calculated as described below.

### Analyses of the Recent Literature in Macrocycle Drug Discovery

PubMed^16^ was searched to get an overview of the extent to which macrocycles has been investigated in medicinal chemistry during the past 17 years (2005–2022, last download May 2022). To this end, all publications with the word “macrocycle” as a keyword in the leading 20 journals in medicinal chemistry were retrieved and manually examined (*n* = 853). The journals were selected based on Google Scholar’s h5-index (h-index for articles published in the last 5 complete years) for the category “Medicinal chemistry”. Thus, *Journal of Medicinal Chemistry*, *European Journal of Medicinal Chemistry*, *Drug Discovery Today*, *Current Medicinal Chemistry*, *Natural Products Reports*, *Medicinal Research Reviews*, *Bioorganic & Medicinal Chemistry*, *Expert Opinion on Therapeutic Patents*, *Current Topics in Medicinal Chemistry*, *Bioorganic & Medicinal Chemistry Letters*, *Expert Opinion on Drug Discovery*, *ACS Medicinal Chemistry Letters*, *Journal of Enzyme Inhibition and Medicinal Chemistry*, *ChemMedChem*, *Future Medicinal Chemistry*, *Medicinal Chemistry Communications*, *Mini Reviews in Medicinal Chemistry*, *Chemical Biology & Drug Design*, *Anti-Cancer Agents in Medicinal Chemistry*, and *Journal of Computer-Aided Molecular Design* were examined. Articles were also retrieved from *Nature* and *Nature Chemical Biology*. The set of 853 retrieved articles was manually curated to include articles in which macrocycles with reported structures were investigated for use in one or several therapeutic indications and/or against one or several targets. The resulting 509 articles included 532 entries with reported indications and 555 entries with reported targets.

### Analysis of Binding Site and Ligand Shape

The PDB was searched for target-bound complexes of each macrocycle in the drug and clinical candidate data sets. Then, the shape of the macrocycle binding site of each target was manually classified as described earlier,^6^ that is, each binding site was manually classified based on the interface with the macrocycle. An interaction between a single face of the target protein and the ligand was classified as a flat site, while an interaction which involved two or three faces defined a groove. Moreover, an interaction involving four faces with a well-defined entry and exit defined a tunnel. Finally, the interactions by four or five faces with a well-defined entry was described as a pocket. Protein–ligand complexes were available in the PDB for 34 of the 67 macrocyclic drugs and 5 of the 34 and clinical candidates.

All 39 protein–ligand complexes were imported into MOE (version 2020.09) to remove all counterions, solvent molecules, and salts from the structures. Then, the atomic coordinates of the ligand were extracted from the protein–ligand complex and further curated, including checking protonation, chirality, and missing atoms. Subsequently, PMI (principal moments of inertia) descriptors (NPR1 and NPR2) were computed for the target-bound conformation of each macrocycle using the MOE suite.^79^ The PMI descriptors for the Ro5-compliant reference data set (*n* = 37) were also calculated.^6^

### Analysis of Molecular Property Descriptors

The uncharged SMILES codes for compounds in the three data sets were obtained as described above. The protonation states of the compounds at pH 7.0 were calculated using MarvinSketch (version 22.13.0),^80^ and the SMILES for the major microspecies were generated. Subsequently, a set of 10 molecular descriptors which represent the size, shape, flexibility, and polarity were calculated for both charge states of the macrocycles in the drug and in the clinical candidate data sets. Descriptors were only calculated at pH 7.0 for the macrocycles in the ChEMBL data set. Molecular weight (MW), number of carbon atoms (nC), topological polar surface area (TPSA), number of hydrogen-bond acceptors (HBA) and donors (HBD), and Kier flexibility index (Φ) were calculated using the Dragon software (version 7.0.10);^81^ the number of single rotatable bonds (NRotB) and number of aromatic rings (NAR) were calculated using the DataWarrior (version 5.5.0) tool,^82^ and the logarithms of aqueous solubility (cLogS) and the octanol/water partition coefficient including explicit hydrogen atoms (cLogP) were calculated using MOE. The number of HBDs was calculated by adding up the hydrogen atoms bonded to any nitrogen and oxygen without negative charge in the molecule, and the number of HBAs is the sum of any nitrogen, oxygen, and fluorine atoms. However, nitrogen atoms with a positive formal charge, higher oxidation states, and the pyrrolyl form of nitrogen were excluded from the HBA count. This differs from the original procedure of the Rule of 5,^41^ where the HBA count is obtained as the sum of N and O atoms in the compound. For some compounds, molecular descriptor calculations fail due to the presence of metals in the structures; examples include cyanocobalamin, hydroxycobalamin, lutetium lu-177 dotatate, and lutetium lu-177 vipivotide tetraxetan. For those macrocycles that are administered as a mixture, only the major ingredient was considered. Therefore, only capreomycin IB (67% of the mixture) was taken as capreomycin and only ivermectin B_1A_ (80%) as ivermectin. For polymyxin B, only the B_1_ subtype was considered for the calculations, even though B1 and B_2_ are equally relevant. Finally, since porfirmer sodium is a mixture of oligomeric porphyrins, it was removed from the data set. The PCA module from the factoextra R package was used to investigate the relationship between the compounds using molecular descriptors and visualize the molecular descriptor space. All plots and analysis were made using RStudio (ggplot, caret, factoextra, moonbook, etc.).

### Classification of Oral and Parenteral Macrocycles

The distribution of oral and parenteral macrocycles in the data sets was studied based on the molecular descriptors, calculated as described above. From the intersection point (cutoff) of the oral and parenteral macrocycles distribution or density curve for each molecular descriptor a cutoff value was defined for each property. Based on these cutoffs, the true positive (TP), true negative (TN), false positive (FP), and false negative (FN) classes were determined, with compounds being orally absorbed being counted as positives and parenterally administered ones as negatives. The quality of the different models was assessed using the following statistical parameters, i.e., sensitivity (eq 1), specificity (eq 2), GMean (eq 3), overall accuracy (eq 4), and Cohen’s kappa (eq 5)
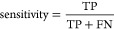
1
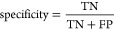
2
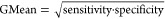
3
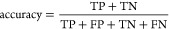
4
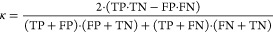
5All classification and statistical analyses were performed using the “Caret” package in R Studio (version 2022.02.3).

In addition to single-molecular descriptor analysis, the best single predictors for the training and test sets were combined and used for the bi-descriptor models (two-parameter combinations) and tridescriptor models (three-parameter combinations). Since tridescriptor models did not produce any better models (data not shown) as compared to bi-descriptor models, only the single descriptor and bi-descriptor models are discussed herein.

## Supporting Information Available

Figures and tables with additional data from the four data sets assembled for this perspective and analysis of that data (PDF)
SMILES codes for the uncharged and pH 7.0 states of the FDA-approved macrocyclic drugs and clinical candidates data sets, and the SMILES codes for the pH 7.0 states of the macrocycles in the ChEMBL data set (CSV)
Articles from the literature of 2005–2022 which report disease indications (CSV)
Articles from the literature of 2005–2022 which report targets for macrocycles (CSV)

## Abbreviations Used

AB-MPS: AbbVie’s multiparameter score
bRo5: beyond the Rule of 5
cLogS: calculated aqueous solubility
CT: clinical trial
CXCR4: C-X-C chemokine receptor type 4
FLT3: FMS-like receptor tyrosine kinase-3
FN: false negative
FP: false positive
GMean: geometric mean
JAK2: Janus kinase 2
κ: Cohen’s kappa coefficient
LptD: lipopolysaccharide transport protein D
MC: macrocycles
Mcl-1: induced myeloid leukemia cell differentiation protein
MECs: minimum energy conformations
MEK: mitogen-activated protein kinase kinase
mTOR: mammalian target of rapamycin
NAR: number of aromatic rings
nC: number of carbon atoms
NPR: natriuretic peptide receptor
NRotB: number of rotatable bonds
OH: alcohol
PHI or Φ: Kier’s flexibility index
PMI: principal moment of inertia
RNAP: RNA polymerase
SMILES: simplified molecular input line-entry system
SSTR2: somatostatin receptor 2
STING: stimulator of interferon genes
TN: true negative
TP: true positive
TPSA: topological polar surface area
TRKs: tropomyosin receptor kinases
PROTACs: proteolysis-targeting chimeras
