# Supplementary material for: Macrocycles in Drug Discovery—Learning from the Past for the Future
Source: J Med Chem. 2023 Apr 5;66(8):5377–96. doi: 10.1021/acs.jmedchem.3c00134 (PMC10150360; doi:10.1021/acs.jmedchem.3c00134)
Supplement: Supplementary file 1 — jm3c00134_si_001.pdf [file jm3c00134_si_001.pdf]

Supporting Information

Macrocycles in Drug Discovery – Learning from the Past for the Future

Diego Garcia Jimenez,[1,2] Vasanthanathan Poongavanam[1] and Jan Kihlberg[1,*]

[1] Department of Chemistry–BMC, Uppsala University, Box 576, SE-751 23 Uppsala, Sweden

[2] Department of Molecular Biotechnology and Health Sciences, University of Torino, Quarello 15, 10135 Torino, Italy

Corresponding author*:

Jan Kihlberg –Email: jan.kihlberg@kemi.uu.se

List of contents

- Supplementary Tables

Supplementary Table 1: classification of **FDA-approved macrocycles** by indication and target.

Supplementary Table 2: subclassification of **FDA-approved macrocycles** into original natural products, natural product derivatives and *de novo* designed.

Supplementary Table 3: binding site classification of **FDA-approved macrocycles**.

Supplementary Table 4: molecular property profile (descriptive statistics) of oral and parenteral **FDA-approved macrocycles**.

Supplementary Table 5: single property models for the differentiation of oral and parenteral **FDA-approved macrocycles**.

Supplementary Table 6: bi-property models for the differentiation of oral and parenteral **FDA-approved macrocycles.**

Supplementary Table 7: AB-MPS score evaluation.

Supplementary Table 8: classification of **macrocycles in clinical trials** by indication and target.

Supplementary Table 9: subclassification of **macrocycles in clinical trials** into original natural products, natural product derivatives and *de novo* designed.

Supplementary Table 10: **Macrocycles in clinical trials** with approved macrocyclic and non-macrocyclic drugs for the same targets.

Supplementary Table 11: binding site classification of **macrocycles in clinical trials**.

Supplementary Table 12: statistical significances for the differentiation of oral and parenteral **macrocycles in clinical trials.**

Supplementary Table 13: bi-property models for the differentiation of oral and parenteral **macrocycles in clinical trials**.

- Supplementary Figures

Supplementary Figure 1: **FDA-approved macrocycles** classified by indication.

Supplementary Figure 2: 2D and 3D representation of **FDA-approved** macrocyclic hepatitis C virus (HCV) inhibitors.

Supplementary Figure 3: molecular property comparison of oral and parenteral **FDA-approved macrocycles**.

Supplementary Figure 4: PCA for the differentiation of oral and parenteral **FDA-approved macrocycles.**

Supplementary Figure 5: PCA for the differentiation of original natural products, natural product derivatives and *de novo* designed **FDA-approved macrocycles.**

Supplementary Figure 6: single property distribution for the differentiation of oral and parenteral **FDA-approved macrocycles.**

Supplementary Figure 7: graphical evaluation of bi-property models.

Supplementary Figure 8: graphical AB-MPS score evaluation.

Supplementary Figure 9: HBD frequencies of **FDA-approved macrocycles** originated from *de novo* design or natural products at pH 7.

Supplementary Figure 10: HBD frequencies of **FDA-approved macrocycles** originated from *de novo* design or natural products at neutral pH.

Supplementary Figure 11: PMI plot illustrating the shapes of the target bound conformations of **macrocycles in clinical trials**.

Supplementary Figure 12: molecular property space of **macrocycles in clinical trials and approved as drugs by the FDA.**

Supplementary Figure 13: PCA for the differentiation of oral and parenteral **macrocycles in clinical trials**.

Supplementary Figure 14: HBD frequencies of **macrocycles in clinical trials** orginated from *de novo* or natural products at pH 7.

Supplementary Figure 15: comparison of the therapeutic indications between **medicinal chemistry articles** published between 2005 and 2022.

Supplementary Figure 16: chronological evolution of studied therapeutic indications in **medicinal chemistry articles** published between 2005 and 2022.

Supplementary Figure 17: target frequency in **medicinal chemistry articles** published between 2005 and 2022.

Supplementary Figure 18: PCA of the chemical space of the **CHEMBL** dataset against the **FDA-approved and clinical trial macrocyclic dataset.**

Supplementary Figure 19: density histogram plots of descriptors calculated for the **CHEMBL, FDA-approved and clinical trial macrocyclic dataset.**

Supplementary Figure 20: density histogram plots of descriptors calculated for the **CHEMBL** dataset and the combined oral and parenteral macrocycles in the **FDA-approved and clinical trial macrocyclic datasets.**

Table S1. Full therapeutic indications and target classification for the FDA-approved macrocyclic drugs dataset (n=72). Five macrocycles (macrocycle names in bold) are duplicated because each is used in two therapeutic indications. The table is ordered by therapeutic indication (alphabetically) and then by target (alphabetically). Complete target names are reported. NA: Target not available.

Therapeutic indication	Target	Drug	FDA approval year	Origin	Absorption
Achondroplasia	Atrial natriuretic peptide receptor (NPR)	Vosoritide	2021	Natural product derivative	Parenteral
Acromegaly	Somatostatin receptor (SSTR)	**Lanreotide**	2007	Natural product derivative	Parenteral
Acute coronary syndrome	Integrin beta-3 (CD61)	Eptifibatide	1998	Natural product derivative	Parenteral
Antidiuretic	Vasopressin receptors (VR)	Desmopressin	1978	Natural product derivative	Oral
	Vasopressin receptors (VR)	Vasopressin	2014	Natural product	Parenteral
Autoimmune diseases	Cyclophilin (CyP),<br>Calcium signal-modulating cyclophilin ligand (CAMLG),<br>Calcineurin subunit B (CNB)	Voclosporin	2021	Natural product derivative	Oral
	Cyclophilin (CyP),<br>Calcium signal-modulating cyclophilin ligand (CAMLG),<br>Calcineurin subunit B (CNB)	**Cyclosporin**	1983	Natural product	Oral
	FKBP12,<br>Calcineurin subunit B (CNB)	**Tacrolimus**	1994	Natural product	Oral
	FKBP12,<br>Calcineurin subunit B (CNB)	Pimecrolimus	2001	Natural product derivative	Parenteral
Chronic Idiopathic Constipation (CIC)	Guanylate cyclase soluble subunit alpha-2 (GUCY1A2)	Plecanatide	2017	Natural product derivative	Parenteral
Chronic pain	Voltage-dependent N-type calcium channel subunit alpha-1B (CACNA1B)	Ziconotide	2004	Natural product	Parenteral
Cushing's disease	Somatostatin receptor (SSTR)	Pasireotide	2012	Natural product derivative	Parenteral

Genetic obesity	Melanocortin receptor (MCR)	Setmelanotide	2020	Natural product derivative	Parenteral
Heart failure	Atrial natriuretic peptide receptor (NPR)	Nesiritide	2001	Natural product	Parenteral
Immunosuppressant	Cyclophilin (CyP), Calcium signal-modulating cyclophilin ligand (CAMLG), Calcineurin subunit B (CNB)	**Cyclosporin**	1983	Natural product	Oral
	FKBP12, Calcineurin subunit B (CNB)	**Tacrolimus**	1994	Natural product	Oral
	FKBP12, Serine/threonine-protein kinase mTOR	**Sirolimus**	1999	Natural product	Oral
	FKBP12, Serine/threonine-protein kinase mTOR	**Everolimus**	2009	Natural product derivative	Oral
Induction of labor	Oxytocin receptor (OXTR)	Oxytocin	1980	Natural product	Parenteral
Infection: Antibacterial	16S/23S rRNA (cytidine-2'-O)-methyltransferase TlyA	Capreomycin	1971	Natural product	Parenteral
	23S ribosomal RNA (50S)	Azithromycin	1991	Natural product derivative	Oral
	23S ribosomal RNA (50S)	Clarithromycin	1991	Natural product derivative	Oral
	23S ribosomal RNA (50S)	Dirithromycin	1995	Natural product derivative	Oral
	23S ribosomal RNA (50S)	Erythromycin	1964	Natural product	Oral
	23S ribosomal RNA (50S)	Telithromycin	2004	Natural product derivative	Oral
	Bacterial membrane	Colistimethate	1970	Natural product	Parenteral
	Bacterial membrane	Daptomycin	2003	Natural product	Parenteral
	Bacterial membrane	Polymyxin B	1951	Natural product	Parenteral
	C55-isoprenyl pyrophosphate	Bacitracin	1948	Natural product	Parenteral
	NAM/NAG peptide (D-Ala-D-Ala)	Dalbavancin	2014	Natural product derivative	Parenteral

	NAM/NAG peptide (D-Ala-D-Ala)	Oritavancin	2014	Natural product derivative	Parenteral
	NAM/NAG peptide (D-Ala-D-Ala)	Telavancin	2009	Natural product derivative	Parenteral
	NAM/NAG peptide (D-Ala-D-Ala)	Vancomycin	1958	Natural product	Parenteral
	RNA polymerase	Fidaxomicin	2011	Natural product	Parenteral
	RNA polymerase	Rifabutin	1992	Natural product derivative	Oral
	RNA polymerase	Rifampicin	1971	Natural product derivative	Oral
	RNA polymerase	Rifamycin	2018	Natural product	Parenteral
	RNA polymerase	Rifapentine	1998	Natural product derivative	Oral
	RNA polymerase	Rifaximin	2004	Natural product derivative	Parenteral
	Streptogramin A acetyltransferase	Dalfopristin	1999	Natural product derivative	Parenteral
Infection: Antifungal	1,3-beta-glucan synthase component (FKS1)	Anidulafungin	2006	Natural product derivative	Parenteral
	1,3-beta-glucan synthase component (FKS1)	Caspofungin	2001	Natural product derivative	Parenteral
	1,3-beta-glucan synthase component (FKS1)	Micafungin	2005	Natural product derivative	Parenteral
	Ergosterol	Amphotericin B	1966	Natural product	Parenteral
	Ergosterol	Natamycin	1978	Natural product	Parenteral
	Ergosterol	Nystatin	1964	Natural product	Parenteral
Infection: Antiparasitic	GABA-A gated chloride channel (GABAA)	Moxidectin	2018	Natural product	Oral
	Glutamate-gated chloride channel (GluCl), GABA-A gated chloride channel (GABAA)	Ivermectin	1996	Natural product	Oral

Infection: Antiviral (Hepatitis C)	HCV NS3/4A protease	Glecaprevir	2017	*De novo*	Oral
	HCV NS3/4A protease	Grazoprevir	2016	*De novo*	Oral
	HCV NS3/4A protease	Paritaprevir	2014	*De novo*	Oral
	HCV NS3/4A protease	Simeprevir	2013	*De novo*	Oral
	HCV NS3/4A protease	Voxilaprevir	2017	*De novo*	Oral
Macular degeneration	NA (Reactive oxygen species) (ROS)	Verteporfin	2000	Natural product derivative	Parenteral
Oncology	ALK receptor	Lorlatinib	2018	*De novo*	Oral
	CXCR4 chemokine receptor	Plerixafor	2008	*De novo*	Parenteral
	DNA	Dactinomycin	1964	Natural product	Parenteral
	Histone deacetylase 1,2 (HDAC)	Romidepsin	2009	Natural product	Parenteral
	NA (Reactive oxygen species) (ROS)	Porfimer sodium	1995	Natural product derivative	Parenteral
	Prostate-specific antigen (PSA)	Lutetium Lu-177 Vipivotide Tetraxetan	2022	Natural product derivative	Parenteral
	FKBP12, Serine/threonine-protein kinase mTOR	**Everolimus**	2009	Natural product derivative	Oral
	FKBP12, Serine/threonine-protein kinase mTOR	**Sirolimus**	1999	Natural product	Oral
	FKBP12, Serine/threonine-protein kinase mTOR	Temsirolimus	2007	Natural product derivative	Parenteral
	Somatostatin receptor (SSTR)	**Lanreotide**	2007	Natural product derivative	Parenteral
	Somatostatin receptor (SSTR)	Lutetium Lu 177 Dotatate	2018	Natural product derivative	Parenteral
	Somatostatin receptor (SSTR)	Octreotide	1988	Natural product derivative	Oral
	Tubulin	Eribulin	2010	Natural product derivative	Parenteral

	Tubulin	Ixabepilone	2007	Natural product derivative	Parenteral
	Tyrosine-protein kinase JAK2<br>Receptor-type tyrosine-protein kinase FLT3	Pacritinib	2022	*De novo*	Oral
Premenopausal women (with hypoactive sexual desire disorder)	Melanocortin receptor (MCR)	Bremelanotide	2019	Natural product derivative	Parenteral
Vitamin B12 deficiency	Methionine synthase (MS)<br>Methylmalonyl-CoA mutase (MCM)<br>Methionine synthase reductase (MTRR) (mitochondrial)	Cyanocobalamin	1942	Natural product	Oral
	Methionine synthase (MS)<br>Methylmalonyl-CoA mutase (MCM)<br>Methionine synthase reductase (MTRR) (mitochondrial)	Hydroxocobalamin	1975	Natural product	Oral

Table S2. Subclassification of FDA-approved macrocyclic drugs into original natural products, natural product derivatives and *de novo* designed. Original natural products have been placed first, followed by families of natural product derivatives and *de novo* designed macrocyclic drugs. The color coding indicates that drugs belonging to compound families have been grouped together.

Drug	Origin	Subclassification	Improvement for derivative	Description	Reference
**Amphotericin B**	Natural	Original natural product			Dutcher, J. D. *Dis. Chest* **1968**, *54*, 296–298.
**Bacitracin**	Natural	Original natural product			Howard, B. *xPharm: The Comprehensive Pharmacology Ref; Elsevier*, **2007**; *pp 1–4*.
**Capreomycin**	Natural	Original natural product			Thomas, M. G.; Chan, Y. A.; Ozanick, S. G. *Antimicrob. Agents Chemother*. **2003**, *47 (9)*, 2823–2830.
**Colistimethate**	Natural	Original natural product			Baron, S.; Hadjadj, L.; Rolain, J.M.; Olaitan, A.O. *Int. J. Antimicrob. Agents* **2016**, *48*, 583–591
**Cyanocobalamin**	Natural	Original natural product			Jägerstad, M.; Arkbåge, K. *In Encyclopedia of Food Sciences and Nutrition; Elsevier*, **2003**; *pp 1419–1427*.
**Dactinomycin**	Natural	Original natural product			Hollstein, U. *Chem. Rev.* **1974**, *74 (6)*, 625–652.
**Daptomycin**	Natural	Original natural product			Miao, V.; Coëffet-LeGal, M.-F.; Brian, P.; Brost, R.; Penn, J.; Whiting, A.; Martin, S.; Ford, R.; Parr, I.; Bouchard, M.; Silva, C. J.; Wrigley, S. K.; Baltz, R. H. *Microbiology* **2005**, *151 (5)*, 1507–1523.
**Fidaxomicin**	Natural	Original natural product			Fidaxomicin. *Drugs R. D.* **2010**, *10 (1)*, 37–45.
**Hydroxocobalamin**	Natural	Original natural product			Jägerstad, M.; Arkbåge, K. *In Encyclopedia of Food Sciences and Nutrition; Elsevier*, **2003**; *pp 1419–1427*.
**Ivermectin**	Natural	Original natural product			C. Campbell, W. *Curr. Pharm. Biotechnol.* **2012**, *13 (6),* 853–865.
**Moxidectin**	Natural	Original natural product			Milton, P.; Hamley, J. I. D.; Walker, M.; Basáñez, M.-G. *Expert Rev. Anti. Infect. Ther.* **2020**, *18 (11),* 1067–1081.
**Natamycin**	Natural	Original natural product			Delves-Broughton, J. *In Encyclopedia of Food Microbiology; Elsevier*, **2014**; *pp 87–91*.
**Nystatin**	Natural	Original natural product			Lyu, X.; Zhao, C.; Hua, H.; Yan, Z. *Drug Des. Devel. Ther.* **2016**, 1161.
**Oxytocin**	Natural	Original natural product			Clark, S. L.; Simpson, K. R.; Knox, G. E.; Garite, T. J. *Am. J. Obstet. Gynecol.* **2009**, *200 (1)*
**Polymyxin B**	Natural	Original natural product			Velkov, T.; Roberts, K. D.; Thompson, P. E.; Li, J. *Future Med. Chem.* **2016**, *8 (10)*, 1017–1025.
**Romidepsin**	Natural	Original natural product			Ueda, H.; Nakajima, H.; Hori, Y.; Fujita, T.; Nishimura, M.; Goto, T.; Okuhara, M. *I. Taxonomy, Fermentation, Isolation, Physico-Chemical and Biological Properties, and Antitumor Activity. J. Antibiot. (Tokyo)*. **1994**, *47 (3)*, 301–310.
**Tacrolimus**	Natural	Original natural product			Tyagi, M.; Begnini, F.; Poongavanam, V.; Doak, B. C.; Kihlberg, Chem. Eur. J. **2020**, *26 (1)*, 49–88.
**Ziconotide**	Natural	Original natural product			Lee, M. C.; Abrahams, M. *In Clinical Pharmacology; Elsevier*, **2012**; *pp 278–294*.

**Nesiritide**	Natural	Original natural product			Elkayam, U.; Akhter, M. W.; Tummala, P.; Khan, S.; Singh, J. *Cardiovasc. Pharmacol. Ther.* **2002**, *7 (3)*, 181–194.
**Vasopressin**	Natural	Original natural product			Demiselle, J.; Fage, N.; Radermacher, P.; Asfar, P. *Ann. Intensive Care* **2020**, *10 (1)*, 9.
**Desmopressin**	Natural	Natural product derivative (vasopressin)	PK	More resistant to proteases	- Glavaš, M.; Gitlin-Domagalska, A.; Dębowski, D.; Ptaszyńska, N.; Łęgowska, A.; Rolka, K. Int. J. Mol. Sci. 2022, 23 (6), 3068.<br>- M.; Neyer, G.; Hargrove, D. M.; Schteingart, C. D. *J. Med. Chem.* **2019**, *62* (*10*), 4991–5005.
**Pimecrolimus**	Natural	Natural product derivative (ascomycin)	PD/PK	Highly lipophilic derivative to treat topically atopic dermatitis (cream)	Paul, C.; Graeber, M.; Stuetz, A. *Expert Opin. Investig. Drugs* **2000**, *9 (1)*, 69–77.
**Eptifibatide**	Natural	Natural product derivative (barbourin)	PD	Smaller derivative of barbourin to minimize the immunogenic potential and maintain potency	Scarborough, R. M. *Am. Heart J.* **1999**, *138 (6),* 1093–1104.
**Vosoritide**	Natural	Natural product derivative (CNP)		No data	Duggan, S. *Drugs* **2021**, *81 (17)*, 2057–2062.
**Cyclosporin**	Natural	Original natural product			Tribe, H. T. *Mycologist* **1998**, *12 (1)*, 20–22.
**Voclosporin**	Natural	Natural product derivative (cyclosporine)	PD/PK	Attempt to identify a compound with improved efficacy, metabolic stability and safety	Van Gelder, T.; Huizinga, R. B.; Lisk, L.; Solomons, *Nephrol. Dial. Transplant.* **2022**, *37 (5)*, 917–922.
**Anidulafungin**	Natural	Natural product derivative (echinocandin B)	PD/PK	More effective and water soluble	Bartlett, M. S.; Current, W. L.; Goheen, M. P.; Boylan, C. J.; Lee, C. H.; Shaw, M. M.; Queener, S. F.; Smith, J. W. *Antimicrob. Agents Chemother*. **1996**, *40 (8)*, 1811–1816.
**Ixabepilone**	Natural	Natural product derivative (epothilone B)	PD/PK	Higher *in vivo* eficacy, water solubility, metabolic stability and lower protein binding	Lee, F. Y. F.; Borzilleri, R.; Fairchild, C. R.; Kamath, A.; Smykla, R.; Kramer, R.; Vite, G. *Cancer Chemother. Pharmacol.* **2008**, *63 (1)*, 157–166.
**Erythromycin**	Natural	Original natural product			- Doak, B. C.; Over, B.; Giordanetto, F.; Kihlberg, *J. Chem. Biol.* **2014**, *21 (9),* 1115–1142.<br>- L. Katz, G. W. Ashley, *Chem. Rev.* **2005**, *105*, 499– 528.
**Azithromycin**	Natural	Natural product derivative (erythromycin)	PK	Improved oral bioavailability and extended half-life in plasma	- Doak, B. C.; Over, B.; Giordanetto, F.; Kihlberg, *J. Chem. Biol.* **2014**, *21 (9),* 1115–1142.<br>- L. Katz, G. W. Ashley, *Chem. Rev.* **2005**, *105*, 499– 528.
**Clarithromycin**	Natural	Natural product derivative (erythromycin)	PK	Improved oral bioavailability and extended half-life in plasma	- Doak, B. C.; Over, B.; Giordanetto, F.; Kihlberg, *J. Chem. Biol.* **2014**, *21 (9),* 1115–1142<br>- L. Katz, G. W. Ashley, *Chem. Rev.* **2005**, *105*, 499– 528.

**Dirithromycin**	Natural	Natural product derivative (erythromycin)	PK	Higher elimination half-life allowing higher daily dosing and more prolonged tissue concentrations	- Doak, B. C.; Over, B.; Giordanetto, F.; Kihlberg, J. Chem. Biol. 2014, 21 (9), 1115–1142. - L. Katz, G. W. Ashley, *Chem. Rev*. **2005**, *105*, 499– 528.<br>- F. T. Counter, P. W. Ensminger, D. A. Preston, C. Y. Wu, J. M. Greene, A. M. Felty-Duckworth, J. W. Paschal, H. A. Kirst, *Antimicrob. Agents Chemother*. **1991**, *35*, 1116 –1126<br>- Wintermeyer, S. M.; Abdel-Rahman, S. M.; Nahata, M. C. *Ann. Pharmacother.* **1996**, 30 (10), 1141–1149.
**Telithromycin**	Natural	Natural product derivative (erythromycin)	PD	Improve antibacterial spectrum	- Doak, B. C.; Over, B.; Giordanetto, F.; Kihlberg, J. Chem. Biol. 2014, 21 (9), 1115–1142. L. Katz, G. W. Ashley, *Chem. Rev*. **2005**, *105*, 499–528.<br>- Denis, A.; Agouridas, C.; Auger, J.-M.; Benedetti, Y.; Bonnefoy, A.; Bretin, F.; Chantot, J.-F.; Dussarat, A.; Fromentin, C.; Gouin D'Ambrières, S.; Lachaud, S.; Laurin, P.; Le Martret, O.; Loyau, V.; Tessot, N.; Pejac, J.-M.; Perron, S. *Bioorg. Med. Chem. Lett*. **1999**, *9 (21)*, 3075–3080.
**Micafungin**	Natural	Natural product derivative (FR901379)	PD	Improved antifungal action and reduced hemolysis.	Fujie, A. *Pure Appl. Chem.* **2007**, *79 (4)*, 603–614.
**Eribulin**	Natural	Natural product derivative (halichondrin B)	PK	Improved stability	Swami, U.; Chaudhary, I.; Ghalib, M. H.; Goel, *Crit. Rev. Oncol. Hematol.* **2012**, *81 (2)*, 163–184.
**Lutetium lu 177 dotatate**	Natural	Natural product derivative (octreotate)	PD	Higher activity	Das, S.; Al-Toubah, T.; El-Haddad, G.; Strosberg, J. *Expert Rev. Gastroenterol. Hepatol.* **2019**, *13 (11)*, 1023–1031.
**Caspofungin**	Natural	Natural product derivative (pneumocandin B0)	PD/PK	Improved potency, water solubility, and half-life	Balkovec, J. M.; Hughes, D. L.; Masurekar, P. S.; Sable, C. A.; Schwartz, R. E.; Singh, S. B. *Nat. Prod. Rep.* **2014**, *31 (1)*, 15–34.
**Porfimer sodium**	Natural	Natural product derivative (porphyrin)		No data	Marcus, S. L.; McIntyre, W. R. Photodynamic Therapy Systems and Applications. *Expert Opin. Emerg. Drugs* **2002**, *7 (2)*, 321–334.
**Verteporfin**	Natural	Natural product derivative (porphyrin)		No data	Scott, L. J.; Goa, K. L. *Drugs Aging* **2000**, *16 (2)*, 139–146.
**Lutetium lu-177 vipivotide tetraxetan**	Natural	Natural product derivative (PSMA)		No data	Neels, O. C.; Kopka, K.; Liolios, C.; Afshar-Oromieh, A. *Cancers (Basel).* **2021**, *13 (24),* 6255.
**Rifamycin**	Natural	Original natural product			Floss, H. G.; Yu, T.-W. *Chem. Rev.* **2005**, *105 (2)*, 621–632.
**Rifabutin**	Natural	Natural product derivative (rifamycin)	PK	Improved oral bioavailability (higher)	Tyagi, M.; Begnini, F.; Poongavanam, V.; Doak, B. C.; Kihlberg, *Chem. Eur. J.* **2020**, *26 (1)*, 49–88.
**Rifampicin**	Natural	Natural product derivative (rifamycin)	PK	Improved oral bioavailability (higher)	Tyagi, M.; Begnini, F.; Poongavanam, V.; Doak, B. C.; Kihlberg, *Chem. Eur. J.* **2020**, *26 (1)*, 49–88.
**Rifapentine**	Natural	Natural product	PK	Improved oral bioavailability	Tyagi, M.; Begnini, F.; Poongavanam, V.; Doak, B. C.; Kihlberg, *Chem. Eur. J.* **2020**, *26 (1)*, 49–88.

		derivative (rifamycin)		(higher)	
**Rifaximin**	Natural	Natural product derivative (rifamycin)	PK	Improved oral bioavailability (lower)	- Brufani, M.; Cellai, L.; Marchi, E.; Segre, A. *J. Antibiot. (Tokyo)*. **1984**, *37 (12)*, 1611–1622.<br>- Adachi, J. A.; DuPont, H. L. Rifaximin: A *Clin. Infect. Dis.* **2006**, *42 (4)*, 541–547.
**Sirolimus**	Natural	Original natural product			Tyagi, M.; Begnini, F.; Poongavanam, V.; Doak, B. C.; Kihlberg, *Chem. Eur. J.* **2020**, *26 (1),* 49–88.
**Everolimus**	Natural	Natural product derivative (sirolimus)	PK	Improved PK (solubility and oral bioavailability)	Tyagi, M.; Begnini, F.; Poongavanam, V.; Doak, B. C.; Kihlberg, *Chem. Eur. J.* **2020**, *26 (1),* 49–88.
**Temsirolimus**	Natural	Natural product derivative (sirolimus)	PK	Sirolimus adaptation to intravenous administration (prodrug)	Hardcastle, I. R. I*n Comprehensive Medicinal Chemistry III; Elsevier,* **2017**; *pp 154–201.*
**Lanreotide**	Natural	Natural product derivative (somatostatin)		No data	Wolin, E. M.; Manon, A.; Chassaing, C.; Lewis, A.; Bertocchi, L.; Richard, J.; Phan, A. T. *J. Gastrointest. Cancer* **2016**, *47 (4)*, 366–374.
**Octreotide**	Natural	Natural product derivative (somatostatin)		No data	Anthony, L.; Freda, P. U. *Curr. Med. Res. Opin.* **2009**, *25 (12)*, 2989–2999.
**Pasireotide**	Natural	Natural product derivative (somatostatin)		No data	Bolanowski, M.; Kałużny, M.; Witek, P.; Jawiarczyk-Przybyłowska, A. *Rev. Endocr. Metab. Disord.* **2022**, *23 (3)*, 601–620.
**Dalfopristin**	Natural	Natural product derivative (streptogramin)	PK	Water soluble derivative	Alihodžić, S.; Bukvić, M.; Elenkov, I. J.; Hutinec, A.; Koštrun, S.; Pešić, D.; Saxty, G.; Tomašković, L.; Žiher, D. *T.-P. in M. C., Eds.; Elsevier,* **2018**; *Vol. 57*, pp 113–233.
**Plecanatide**	Natural	Natural product derivative (uroguanylin)		No data	Al-Salama, Z. T.; Syed, Y. Y. *Drugs* **2017**, *77 (5)*, 593–598
**Vancomycin**	Natural	Original natural product			Chen, A. Y.; Zervos, M. J.; Vazquez, J. A. *Int. J. Clin. Pract.* **2007**, *61 (5)*, 853–863.
**Dalbavancin**	Natural	Natural product derivative (vancomycin)	PD	Improved potency	Chen, A. Y.; Zervos, M. J.; Vazquez, J. A. *Int. J. Clin. Pract.* **2007**, *61 (5)*, 853–863.
**Oritavancin**	Natural	Natural product derivative (vancomycin)	PD	Improved potency	Domenech, O.; Francius, G.; Tulkens, P. M.; Van Bambeke, F.; Dufrêne, Y.; Mingeot-Leclercq, M.-P. *Biochim. Biophys. Acta - Biomembr.* **2009**, *1788 (9)*, 1832–1840.
**Telavancin**	Natural	Natural product derivative (vancomycin)	PD	Improved potency	Higgins, D. L.; Chang, R.; Debabov, D. V.; Leung, J.; Wu, T.; Krause, K. M.; Sandvik, E.; Hubbard, J. M.; Kaniga, K.; Schmidt, D. E.; Gao, Q.; Cass, R. T.; Karr, D. E.; Benton, B. M.; Humphrey, P. P. *Antimicrob. Agents Chemother.* **2005**, *49 (3)*,
**Bremelanotide**	Natural	Natural product derivative (α-MSH)		No data	Dhillon, S.; Keam, S. J. *Drugs* **2019**, *79 (14)*, 1599–1606.
**Setmelanotide**	Natural	Natural product derivative (α-MSH)		No data	Yeo, G. S. H.; Chao, D. H. M.; Siegert, A.-M.; Koerperich, Z. M.; Ericson, M. D.; Simonds, S. E.; Larson, C. M.; Luquet, S.; Clarke, I.; Sharma, S.; Clément, K.; Cowley, M. A.; Haskell-Luevano, C.; Van Der Ploeg, L.; Adan, R. A. H. *Mol. Metab.* **2021**, *48*, 101206.

**Glecaprevir**	*De novo*	Structure-based design			Hong, J.; Wright, R. C.; Partovi, N.; Yoshida, E. M.; Hussaini, T. *J. Clin. Transl. Hepatol.* **2020**, *8 (3),* 1–14.
**Grazoprevir**	*De novo*	Structure-based design			Hong, J.; Wright, R. C.; Partovi, N.; Yoshida, E. M.; Hussaini, T. *J. Clin. Transl. Hepatol.* **2020**, *8 (3),* 1–14.
**Paritaprevir**	*De novo*	Structure-based design			McDaniel, K. F.; Ku, Y.-Y.; Sun, Y.; Chen, H.-J.; Shanley, J.; Middleton, T.; Or, Y. S.; *Springer International Publishing: Cham,* **2019**; *pp 389–413.*
**Simeprevir**	*De novo*	Structure-based design			Rosenquist, Å.; Samuelsson, B.; Johansson, P.-O.; Cummings, M. D.; Lenz, O.; Raboisson, P.; Simmen, K.; Vendeville, S.; de Kock, H.; Nilsson, M.; Horvath, A.; Kalmeijer, R.; de la Rosa, G.; Beumont-Mauviel, M. *J. Med. Chem.* **2014**, *57 (5)*, 1673–1693.
**Voxilaprevir**	*De novo*	Structure-based design			Hong, J.; Wright, R. C.; Partovi, N.; Yoshida, E. M.; Hussaini, T. *J. Clin. Transl. Hepatol.* **2020**, *8 (3),* 1–14.
**Pacritinib**	*De novo*	Structure-based design			Hart, S.; Goh, K. C.; Novotny-Diermayr, V.; Hu, C. Y.; Hentze, H.; Tan, Y. C.; Madan, B.; Amalini, C.; Loh, Y. K.; Ong, L. C.; William, A. D.; Lee, A.; Poulsen, A.; Jayaraman, R.; Ong, K. H.; Ethirajulu, K.; Dymock, B. W.; Wood, J. W. *Leukemia* **2011**, *25 (11),* 1751–1759.
**Plerixafor**	*De novo*	Serendipity			- DiPersio, J. F.; Uy, G. L.; Yasothan, U.; Kirkpatrick, P. *Nat. Rev. Drug Discov.* **2009**, *8 (2)*, 105–107.<br>- De Clercq, E. *Nat. Rev. Drug Discov.* **2003**, *2 (7),* 581–587.
**Lorlatinib**	*De novo*	Structure-based design			- Johnson, T. W.; Richardson, Edwards, M. P. *J. Med. Chem.* **2014**, *57* (11),

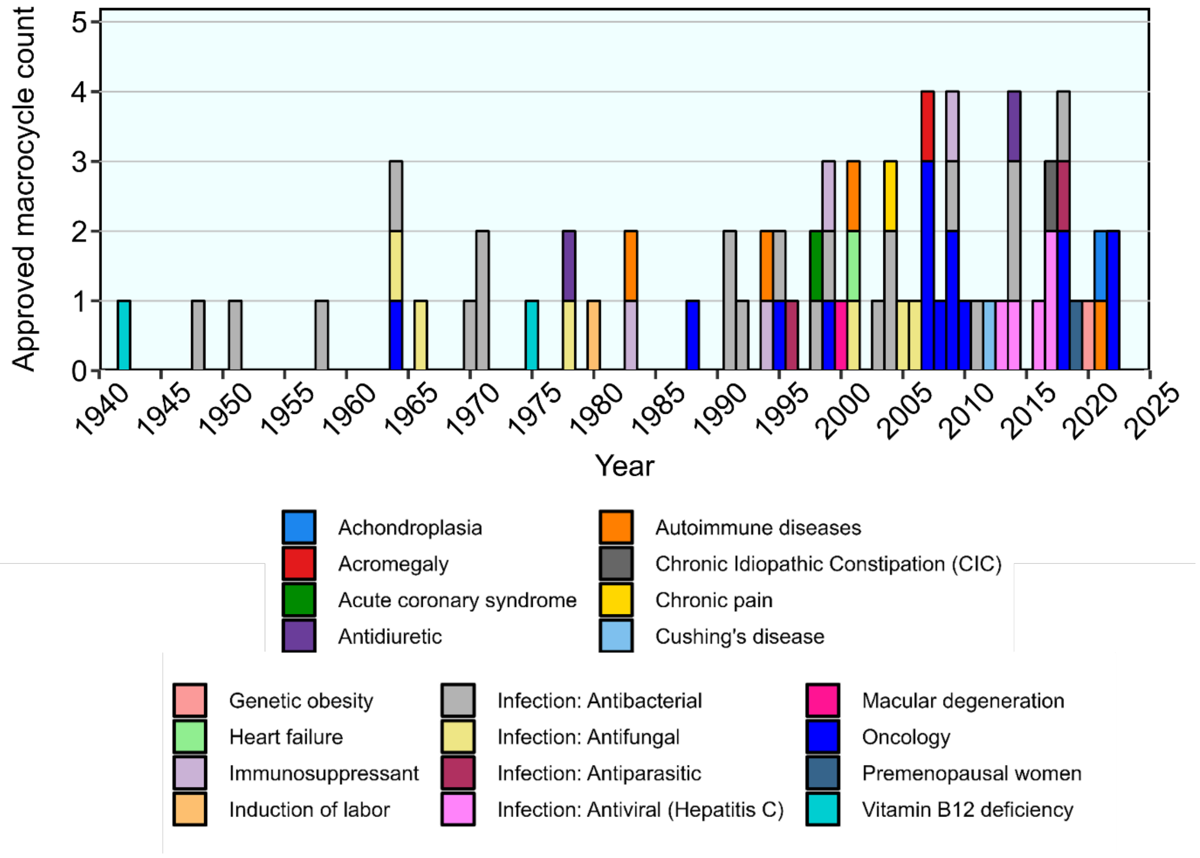

Figure S1. FDA-approved macrocyclic drugs classified by indication and approval year (Table S1).

Table S3. Classification of the binding site shape of the targets of the macrocyclic drugs (n=34).

Drug	PDB ID	Method	Resolution (Å)	Binding site
Azithromycin	4V7Y	X-Ray	3.0	Tunnel
Bacitracin	4K7T	X-Ray	1.1	Unknown
Bremelanotide	7F55	Cryo-EM	3.1	Pocket
Capreomycin	5V93	Cryo-EM	4.0	Groove
Clarithromycin	1J5A	X-Ray	3.5	Tunnel
Cyclosporin	1MF8	X-Ray	3.1	Groove
Dactinomycin	1I3W	X-Ray	1.7	Groove
Dalbavancin	3RUL	X-Ray	2.5	Unknown
Dalfopristin	4U24	X-Ray	2.9	Tunnel
Dirithromycin	6XZ7	Cryo-EM	2.1	Tunnel
Eptifibatide	2VDN	X-Ray	2.9	Groove
Eribulin	5JH7	X-Ray	2.3	Groove
Erythromycin	1JZY	X-Ray	3.5	Tunnel
Fidaxomicin	6FBV	Cryo-EM	3.5	Tunnel
Glecaprevir	6P6L	X-Ray	1.7	Flat
Grazoprevir	3SUD	X-Ray	2.0	Flat
Ivermectin	3RIF	X-Ray	3.4	Groove
Ixabepilone	7DAF	X-Ray	2.4	Groove
Lorlatinib	4CLI	X-Ray	2.1	Groove
Octreotide	7T11	Cryo-EM	2.7	Pocket
Oxytocin	7RYC	Cryo-EM	2.9	Pocket
Rifabutin	2A68	X-Ray	2.5	Tunnel
Rifampicin	6CCV	X-Ray	3.1	Tunnel
Rifamycin	4OIR	X-Ray	3.1	Tunnel
Rifapentine	2A69	X-Ray	2.5	Tunnel
Setmelanotide	7PIU	Cryo-EM	2.6	Pocket
Simeprevir	3KEE	X-Ray	2.4	Flat
Sirolimus	4DRI	X-Ray	1.5	Groove
Tacrolimus	6TZ7	X-Ray	2.5	Groove
Telithromycin	6XHY	X-Ray	2.6	Tunnel
Vancomycin	1FVM	X-Ray	1.8	Unknown
Voclosporin	3ODI	X-Ray	2.2	Groove
Voxilaprevir	6NZT	X-Ray	1.4	Flat
Ziconotide	7VFU	Cryo-EM	3.0	Groove

Table S4. Complete 2D property profile for the FDA-approved macrocyclic drugs dataset (n=62). Molecular property abbreviations are provided in the main text.

	Descriptive statistics	MW (Da)	cLogP	TPSA (Å²)	HBA	HBD	NRotB	nC	NAR	Φ	cLogS
Oral macrocycles (n=24)	Number of values	24.0	24.0	24.0	24.0	24.0	24.0	24.0	24.0	24.0	24.0
	Minimum	406.5	-3.5	69.9	6.0	1.0	0.0	21.0	0.0	5.0	-12.1
	25% Percentile	751.1	3.2	179.4	13.0	2.0	6.0	38.0	0.0	11.7	-8.1
	**Median**	**830.1**	**4.4**	**201.1**	**14.0**	**3.0**	**8.0**	**42.5**	**1.5**	**15.3**	**-7.0**
	75% Percentile	905.0	6.1	220.5	16.5	6.0	10.8	47.8	2.0	20.4	-5.6
	Maximum	1215.0	6.9	487.8	25.0	19.0	20.0	63.0	4.0	34.0	-3.5
	Range	808.4	10.4	417.8	19.0	18.0	20.0	42.0	4.0	29.0	8.6
	Mean	833.6	4.2	211.8	14.8	4.9	8.8	42.9	1.3	16.7	-6.9
	Std. Deviation	185.4	2.3	84.1	4.5	4.4	4.6	9.2	1.4	7.2	2.2
	Std. Error of Mean	37.8	0.5	17.2	0.9	0.9	0.9	1.9	0.3	1.5	0.4
Parenteral macrocycles (n=38)	Number of values	38.0	38.0	38.0	38.0	38.0	38.0	38.0	38.0	38.0	38.0
	Minimum	506.8	-16.9	97.0	4.0	2.0	2.0	24.0	0.0	8.9	-26.2
	25% Percentile	771.5	-3.4	226.9	13.0	6.0	5.5	40.8	0.0	16.4	-11.7
	**Median**	**1072.0**	**1.0**	**381.2**	**22.0**	**15.5**	**16.0**	**52.0**	**1.0**	**23.9**	**-8.4**
	75% Percentile	1492.0	3.8	567.5	32.3	22.0	24.3	65.3	4.0	32.3	-6.1
	Maximum	4109.0	6.2	1842.0	97.0	78.0	115.0	176.0	7.0	126.5	-0.7
	Range	3603.0	23.2	1745.0	93.0	76.0	113.0	152.0	7.0	117.6	25.5
	Mean	1259.0	-0.3	483.3	26.2	18.1	20.7	57.8	2.1	30.2	-9.5
	Std. Deviation	754.6	5.9	382.8	19.3	16.9	24.8	30.2	2.1	24.9	5.4
	Std. Error of Mean	122.4	1.0	62.1	3.1	2.7	4.0	4.9	0.3	4.0	0.9

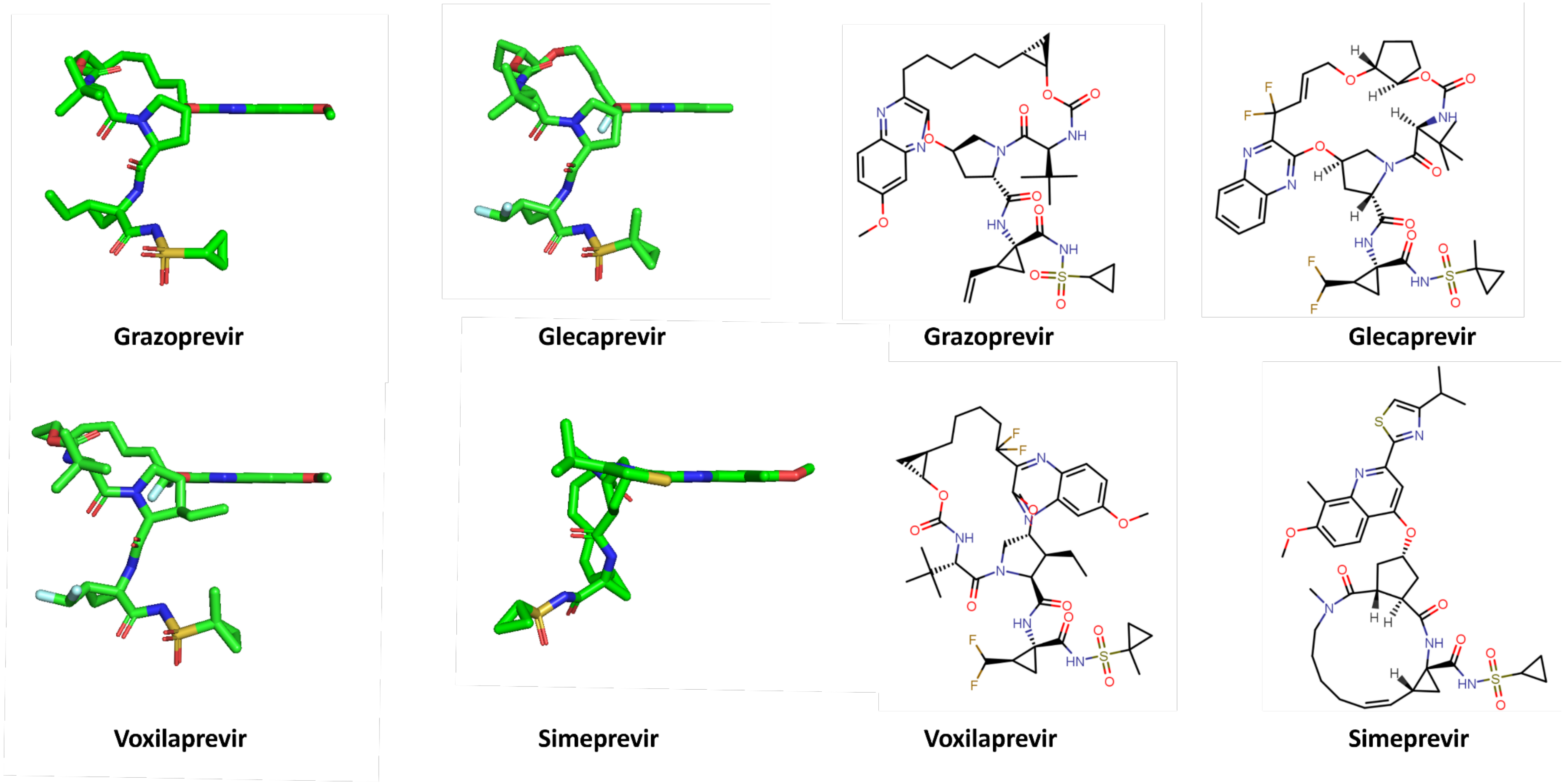

Figure S2. Target bound structures of the FDA-approved macrocyclic hepatitis C virus (HCV) NS3/4A protease inhibitors represented with their aromatic scaffolds aligned. Their 2D structures are also provided.

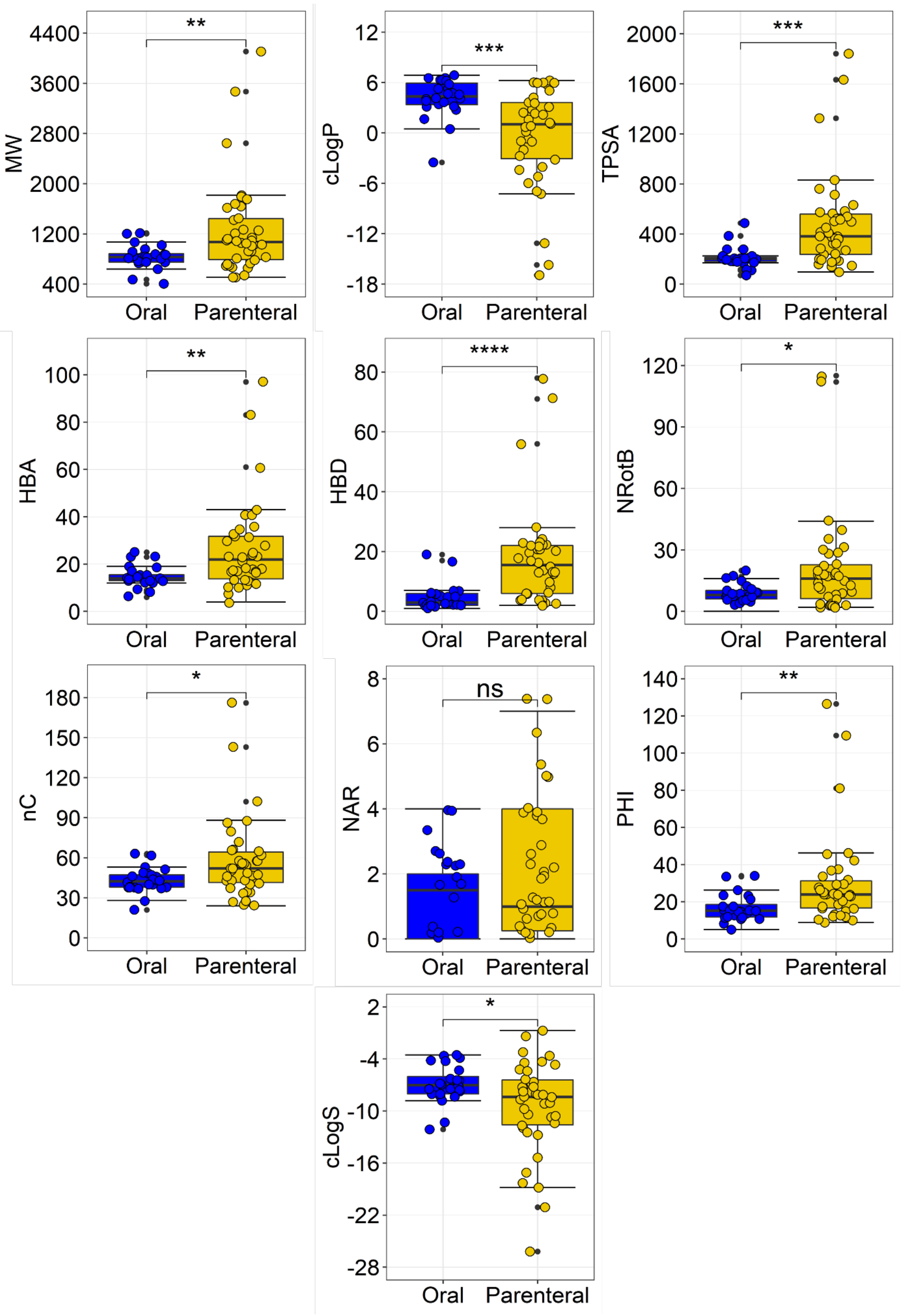
MW
**
4400
3600
2800
2000
1200
400
Oral
Parenteral
cLogP
***
12
6
0
-6
-12
-18
Oral
Parenteral
TPSA
***
2000
1600
1200
800
400
0
Oral
Parenteral
HBA
**
100
80
60
40
20
0
Oral
Parenteral
HBD
****
80
60
40
20
0
Oral
Parenteral
NRotB
*
120
90
60
30
0
Oral
Parenteral
nC
*
180
150
120
90
60
30
0
Oral
Parenteral
NAR
ns
8
6
4
2
0
Oral
Parenteral
PHI
**
140
120
100
80
60
40
20
0
Oral
Parenteral
cLogS
*
2
-4
-10
-16
-22
-28
Oral
Parenteral

Figure S3. Molecular property descriptors calculated at pH 7 for the macrocyclic drugs dataset (n=62), split into two subsets by the route of administration. Those administered orally (n=24) are in blue, while parenterally administered are in gold. Statistical analysis was performed using Wilcoxon´s non-parametric test: p-values: 0-0.0001 (****), 0001-0.001 (***), 0.001-0.01 (**), 0.01-0.05 (*), 0.05-1 (ns). Molecular property abbreviations are provided in the main text: Kier´s flexibility index is abbreviated as PHI or (Φ). Box plots show the 50th percentiles as horizontal bars, the 25th and 75th percentiles as boxes, the 25th percentile minus 1.5 x the interquartile range and the 75th percentile plus 1.5 x the interquartile range as whiskers. Black dots represent values higher than 1.5 x the interquartile range and less than 3 x the interquartile range at either end of the box.

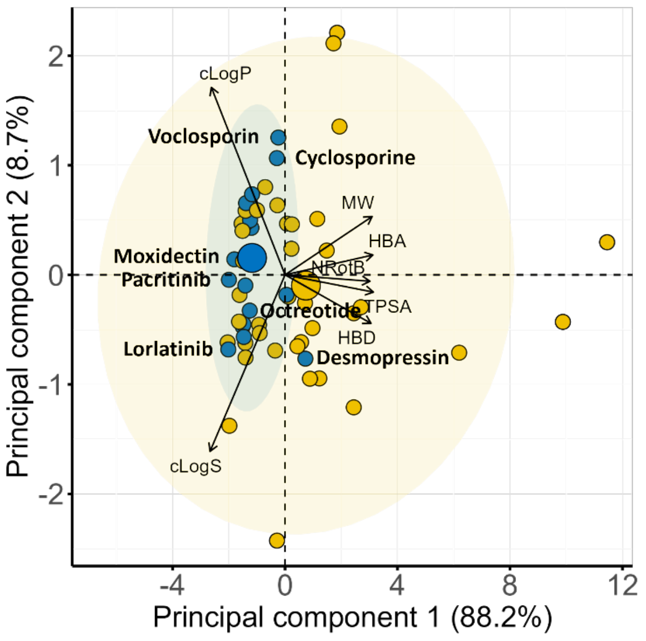

Figure S4. PCA of the complete dataset of macrocyclic drugs (n=62). The PCA was based on the descriptors of Lipinski´s and Veber´s rules, as well as cLogS, calculated at pH 7. Ellipses in blue and yellow shading show the 95% confidence intervals for orally and parenterally administered macrocycles, respectively. The centroid of each class is indicated with a large circle in the color of the respective class. The contributions of individual descriptors to the PCAs are indicated by the length of the arrows.

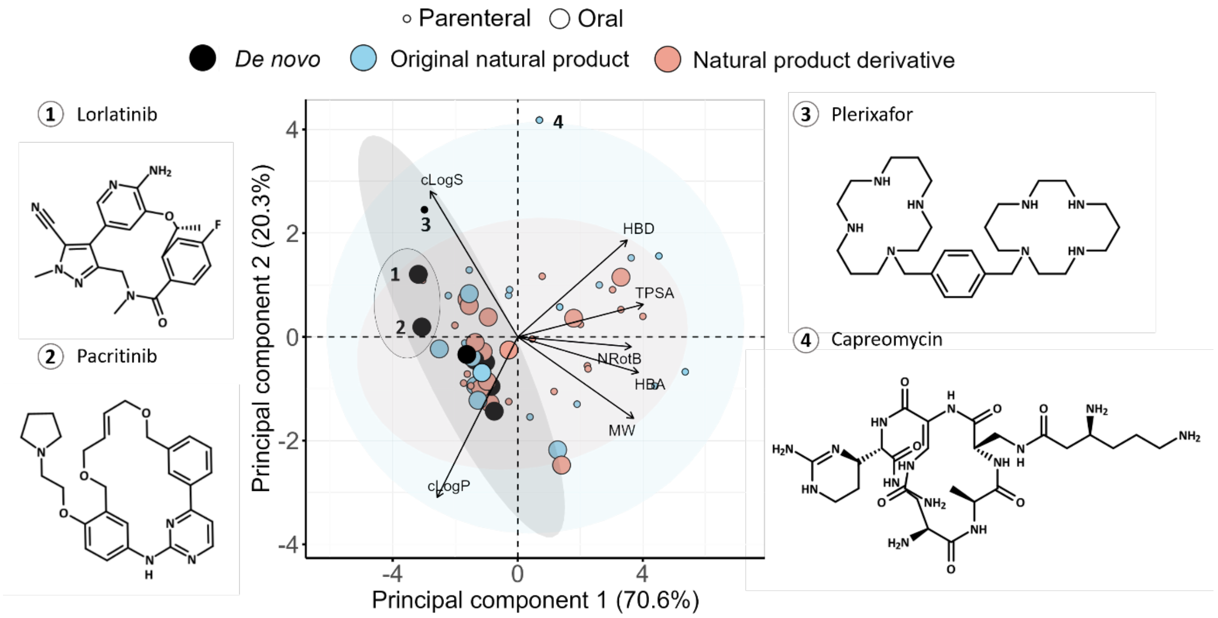

Figure S5. Principal component analysis of the chemical space of the macrocyclic drugs dataset (n=53). The PCA was based on the descriptors of Lipinski´s and Veber´s rules, as well as cLogS, calculated at pH 7. Ellipses in dark grey, blue and red shading show the 95% confidence intervals for the *de novo* designed, original natural product and natural product derivatives subclasses, respectively. The circular marker size is based on the route of administration, with orals and parenterals being large and small, respectively. The contributions of individual descriptors to the PCAs are indicated by the length of the arrows. Nine parenterals with MW >1500 Da were excluded in the PCA to provide a better dissection of the chemical space of the orally bioavailable macrocycles.

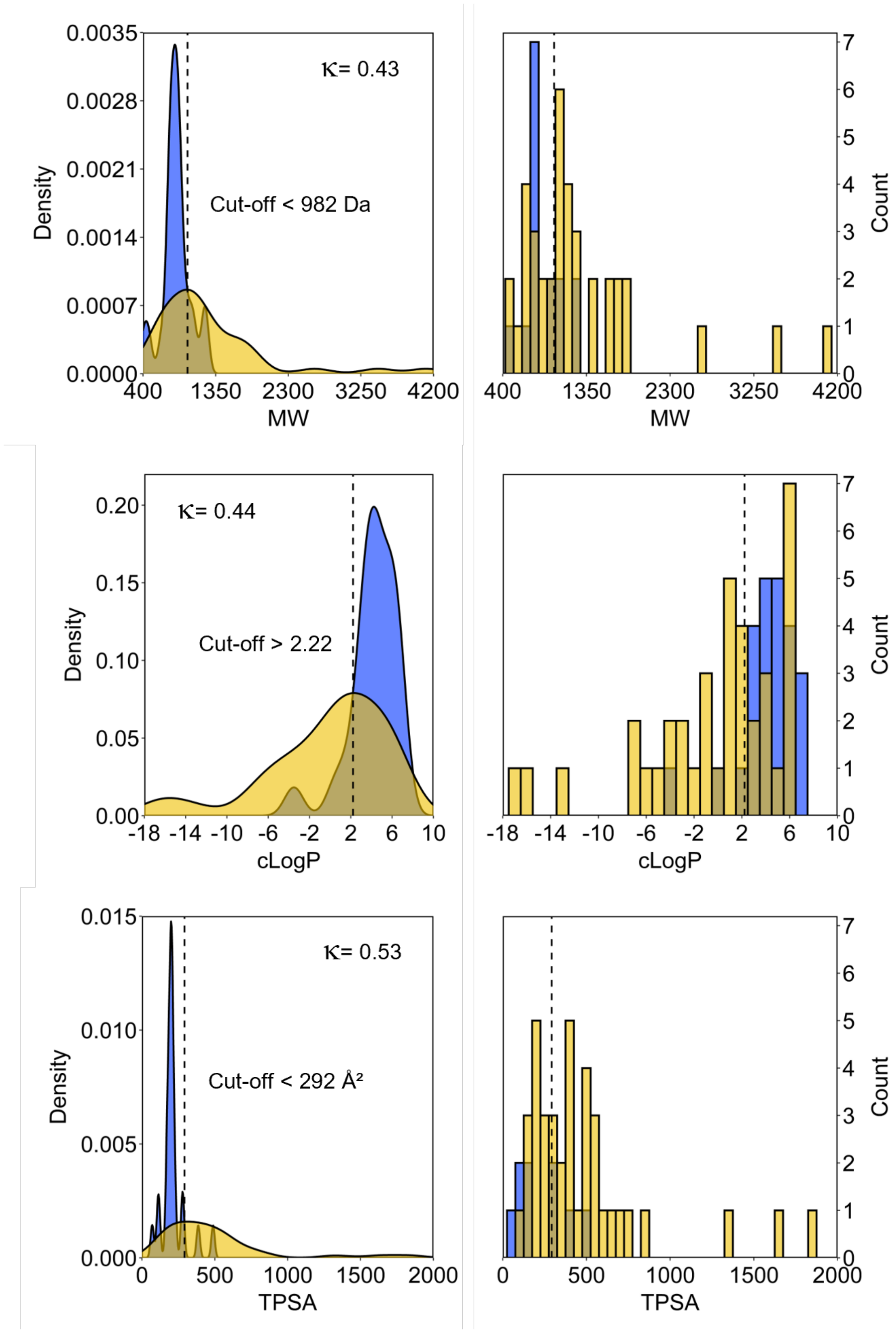
κ= 0.43
Cut-off < 982 Da
Density
MW
Count
κ= 0.44
Cut-off > 2.22
cLogP
κ= 0.53
Cut-off < 292 Å²
TPSA

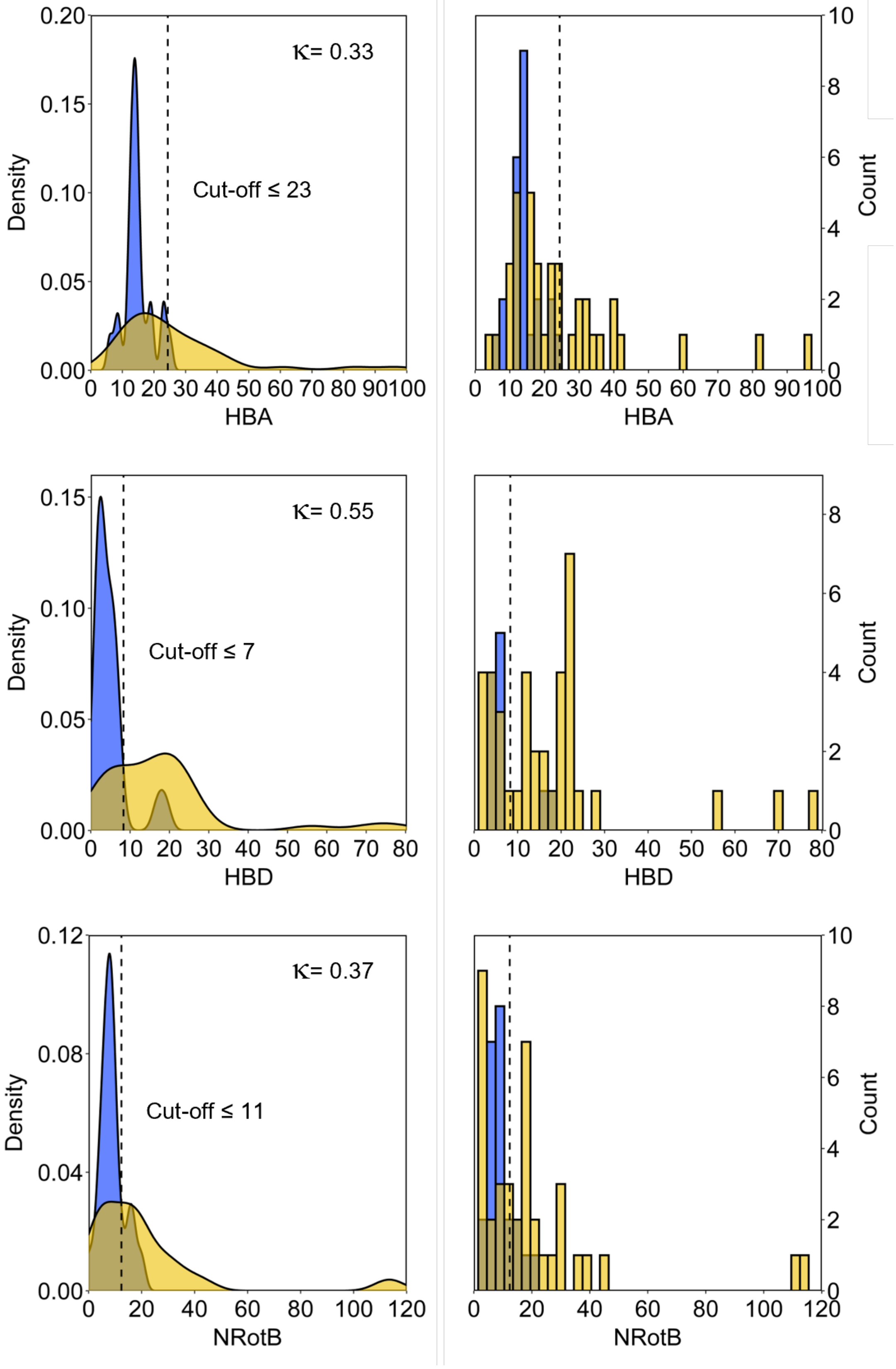

κ= 0.33
Cut-off ≤ 23
Density
HBA
Count
κ= 0.55
Cut-off ≤ 7
HBD
κ= 0.37
Cut-off ≤ 11
NRotB

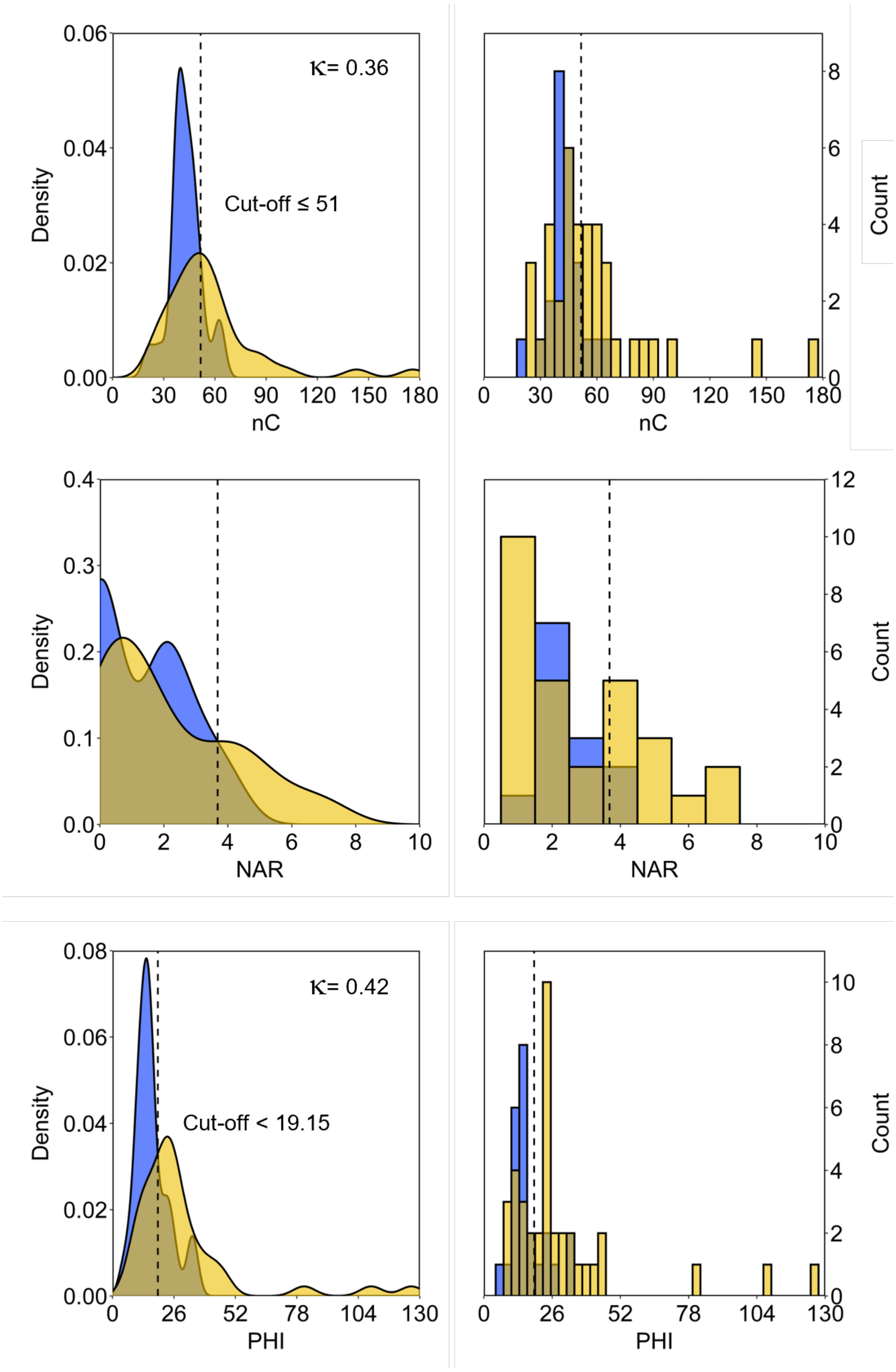
κ= 0.36
Cut-off ≤ 51
Density
nC
Count
NAR
κ= 0.42
Cut-off < 19.15
PHI

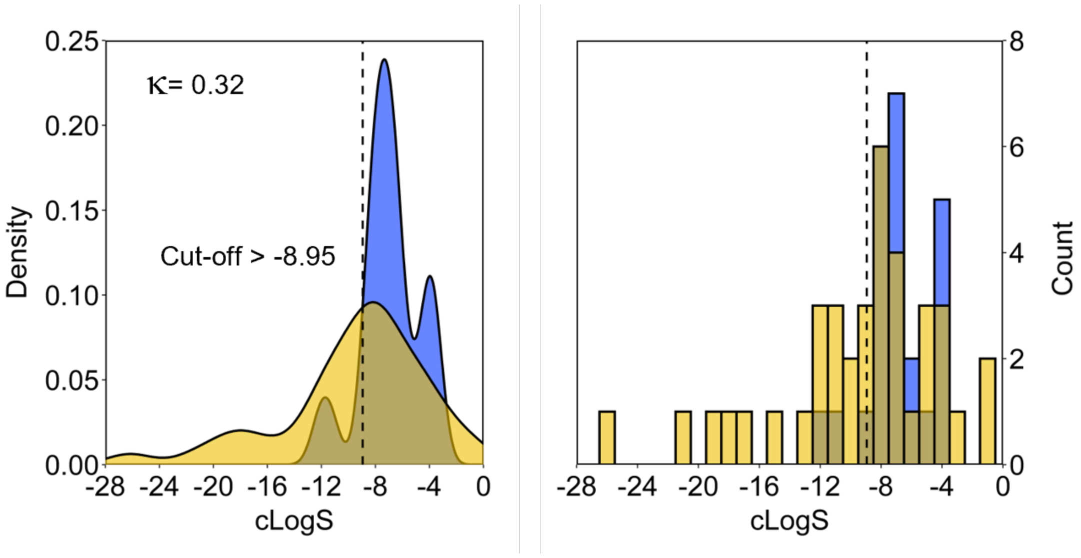

Figure S6. Single property distributions for the oral (blue) and parenteral (gold) subsets of the macrocyclic drugs training set (n=62). The black dashed line indicates the intersection point of the density plot and the derived cut-off value is given adjacent to the dashed line. The reliability of single property models based on each of the three descriptors for the differentiation of oral and parenteral drugs in the training set is given by the Cohen's kappa statistic (κ) value. The cut-off and single model evaluation were not performed for NAR, since the box plot in Figure S3 showed the differences between the oral and parenteral sets were non-significant (ns).

Table S5. Quality of single property models for the differentiation of oral and parenteral macrocyclic drugs in the training set and of macrocyclic clinical candidates in the test set.

Data sets	Molecular property	Cut-off	Confusion matrix				Sens.	Spec.	Acc.	Kappa	GMean
			TP	TN	FP	FN					
Training set (n=62)	MW	< 982	20	24	14	4	0.83	0.63	0.71	0.43	0.73
	cLogP	> 2.22	21	23	15	3	0.88	0.61	0.71	0.44	0.73
	TPSA	< 292	22	25	13	2	0.92	0.66	0.76	0.53	0.78
	HBA	≤ 23	23	16	22	1	0.96	0.42	0.63	0.33	0.64
	HBD	≤7	21	27	11	3	0.88	0.71	0.77	0.55	0.79
	NRotB	≤ 11	19	23	15	5	0.79	0.61	0.68	0.37	0.69
	nC	≤ 51	21	20	18	3	0.88	0.53	0.66	0.36	0.68
	PHI	< 19.15	18	26	12	6	0.75	0.68	0.71	0.42	0.72
	logS	> -8.95	22	17	21	2	0.92	0.45	0.63	0.32	0.64
Test set (n=60)[3,10]	MW	< 982	15	22	20	3	0.83	0.52	0.62	0.28	0.66
	cLogP	> 2.22	17	24	18	1	0.94	0.57	0.68	0.41	0.73
	TPSA	< 292	17	25	17	1	0.94	0.6	0.7	0.43	0.75
	HBA	≤ 23	17	16	26	1	0.94	0.38	0.55	0.23	0.6
	HBD	≤7	18	27	15	0	1	0.64	0.75	0.52	0.8
	NRotB	≤ 11	12	30	12	6	0.67	0.71	0.7	0.35	0.69
	nC	≤ 51	15	21	21	3	0.83	0.5	0.6	0.26	0.65
	PHI	< 19.15	13	29	13	5	0.72	0.69	0.7	0.37	0.71
	logS	> -8.95	14	18	24	4	0.78	0.43	0.53	0.16	0.58

Abbreviations: True Positive (TP), True negative (TN), False Positive (FP), False negative (FN), Sens. (Sensitivity), Spec. (Specificity), Acc. (Accuracy), Kappa (Cohen's kappa) and Geometric Mean (GMean). Positive (P) stands for “Oral” class and negative (N) stands for parenteral class. The test set was obtained from two publications in 2014.[3,10]

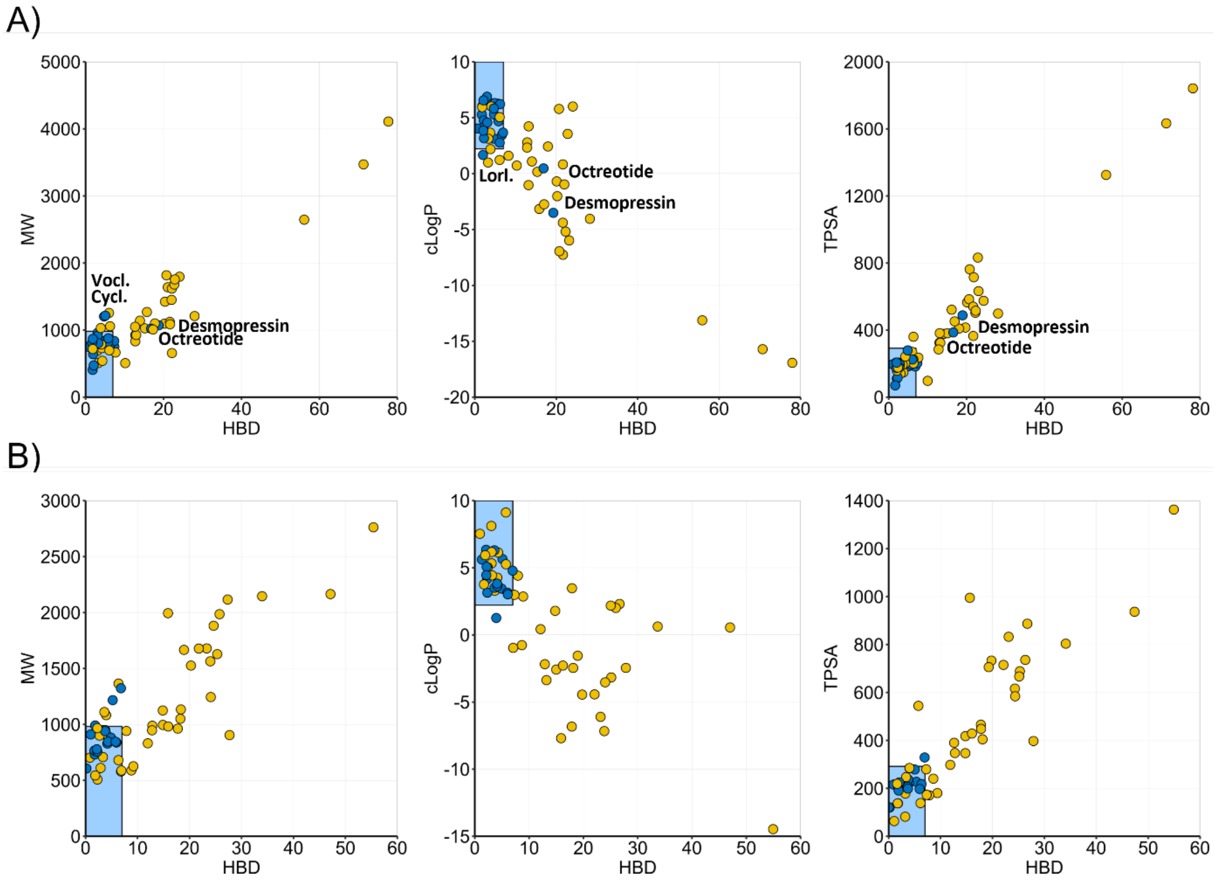

Figure S7. Evaluation of bi-property models for the differentiation of (**A**) macrocyclic drugs (n=62) and (**B**) an external test set of macrocycles not yet approved as drugs in bi-descriptor chemical space (n=60). Oral drugs are indicated by blue circles, while parenterals are in yellow. The filled circles have been jittered slightly to avoid overlap. The test set was obtained from two publications in 2014.[3,10]

Table S6. Quality of bi-property models for prediction of oral and parenteral administration of the macrocyclic drugs in the training set (n=62) and the clinical candidate in the test set (n=60). Cut-off values were selected based on the addition of two descriptors conditions (1st and 2nd cut-off).

Data sets	1st cut-off	2nd cut-off	Confusion matrix				Sens.	Spec.	Acc.	Kappa	GMean
			TP	TN	FP	FN					
Training set (n=62)	MW (< 982)	TPSA	20	28	10	4	0.83	0.74	0.77	0.55	0.78
		cLogP	19	30	8	5	0.79	0.79	0.79	0.57	0.79
		HBD	20	30	8	4	0.83	0.79	0.81	0.6	0.81
	cLogP (> 2.22)	MW	19	30	8	5	0.79	0.79	0.79	0.57	0.79
		TPSA	21	29	9	3	0.88	0.76	0.81	0.61	0.82
		HBD	21	30	8	3	0.88	0.79	0.82	0.64	0.83
	HBD (≤7)	MW	20	30	8	4	0.83	0.79	0.81	0.6	0.81
		cLogP	21	30	8	3	0.88	0.79	0.82	0.64	0.83
		TPSA	22	28	10	2	0.92	0.74	0.81	0.62	0.82
	TPSA (< 292)	MW	20	28	10	4	0.83	0.74	0.77	0.55	0.78
		cLogP	21	29	9	3	0.88	0.76	0.81	0.61	0.82
		HBD	22	28	10	2	0.92	0.74	0.81	0.62	0.82
Test set (n=60)[3,10]	MW (< 982)	TPSA	15	27	15	3	0.83	0.64	0.7	0.4	0.73
		cLogP	14	29	13	4	0.78	0.69	0.72	0.41	0.73
		HBD	15	30	12	3	0.83	0.71	0.75	0.48	0.77
	cLogP (> 2.22)	MW	14	29	13	4	0.78	0.69	0.72	0.41	0.73
		TPSA	16	27	15	2	0.89	0.64	0.72	0.44	0.76
		HBD	17	28	14	1	0.94	0.67	0.75	0.51	0.79
	HBD (≤7)	MW	15	30	12	3	0.83	0.71	0.75	0.48	0.77
		cLogP	17	28	14	1	0.94	0.67	0.75	0.51	0.79
		TPSA	17	28	14	1	0.94	0.67	0.75	0.51	0.79
	TPSA (< 292)	MW	15	27	15	3	0.83	0.64	0.7	0.4	0.73
		cLogP	16	27	15	2	0.89	0.64	0.72	0.44	0.76
		HBD	17	28	14	1	0.94	0.67	0.75	0.51	0.79

Abbreviations: True Positive (TP), True negative (TN), False Positive (FP), False negative (FN), Sens. (Sensitivity), Spec. (Specificity), Acc. (Accuracy), Kappa (Cohen's kappa) and Geometric Mean (GMean). Positive (P) stands for "Oral" class and negative (N) stands for parenteral class. The test set was obtained from two publications in 2014.[3,10]

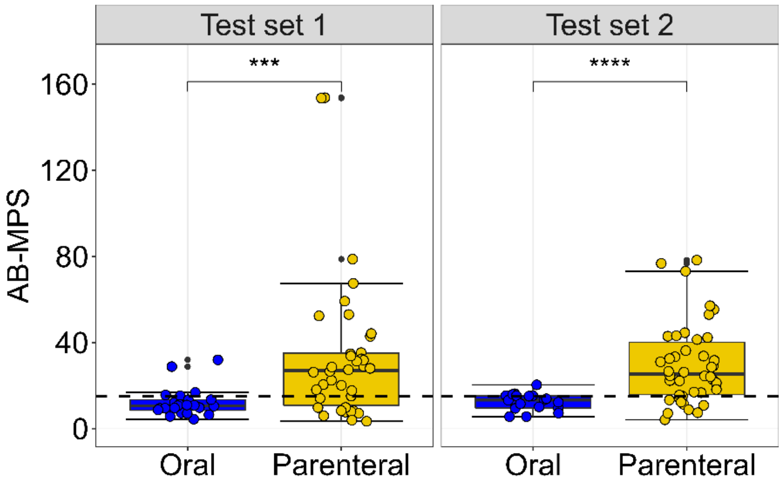

Figure S8. Evaluation of the AB-MPS model for the macrocyclic drugs dataset (Test set 1, original training set, n=62) and test set of macrocycles that were in clinical studies in 2014 (Test set 2, n=60) at pH 7. AB-MPS = Abs(cLogD-3) + NAR + NRotB was used as the equation to calculate AB-MPS value. AB-MPS ≤ 15 was considered as the cut-off for oral or parenteral classification. Oral (blue dots) and parenteral (gold) subsets are compared by Wilcoxon´s non-parametric test. Statistical significance is presented as p-value: 0-0.0001 (****), 0.0001-0.001 (***), 0.001-0.01 (**) and 0.01-0.05 (*),0.05-1 (ns). Box plots show the 50th percentiles as horizontal bars, the 25th and 75th percentiles as boxes, the 25th percentile minus 1.5 x the interquartile range and the 75th percentile plus 1.5 x the interquartile range as whiskers. Black dots represent values higher than 1.5 x the interquartile range and less than 3 x the interquartile range at either end of the box.

Table S7. AB-MPS score evaluation of Test sets 1 and 2 (cf. legend of Figure S8).

AB-MPS score	Cut-off	Confusion matrix				Accuracy	Kappa	Sensitivity	Specificity	GMean
		TP	TN	FP	FN					
Test set 1	AB-MPS ≤ 15	19	27	11	5	0.74	0.48	0.79	0.71	0.75
Test set 2		11	33	9	7	0.73	0.38	0.61	0.79	0.69

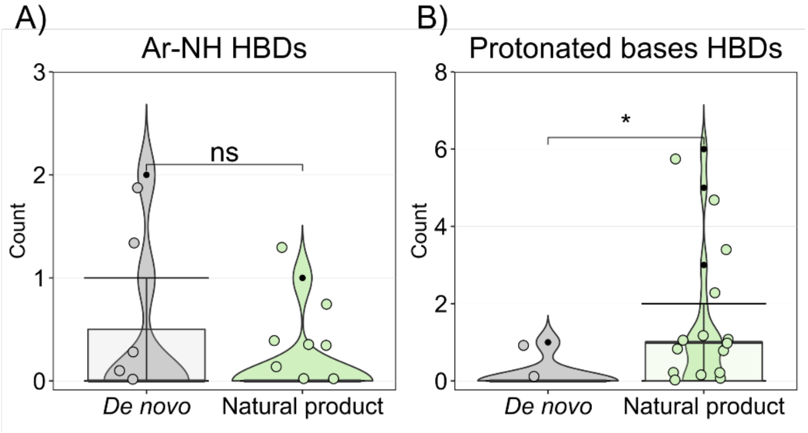

Figure S9. Frequencies of HBDs orginating from (**A**) nitrogen containing heterocycles and (**B**) protonated bases that are positively charged at pH 7 in orally bioavailable macrocyclic drugs discovered by de novo design (n=7) or from natural products (n=17) at pH 7. HBDs from protonated bases originate from aliphatic amines, including piperidines, pyrrolidines, piperazines and 1,3-oxazinanes as well as from guanidines. Box plots show the 50th percentiles as horizontal bars, the 25th and 75th percentiles as boxes, the 25th percentile minus 1.5 x the interquartile range and the 75th percentile plus 1.5 x the interquartile range as whiskers. Black dots represent values higher than 1.5 x the interquartile range and less than 3 x the interquartile range at either end of the box. Violin shapes represent the data density at each count value.

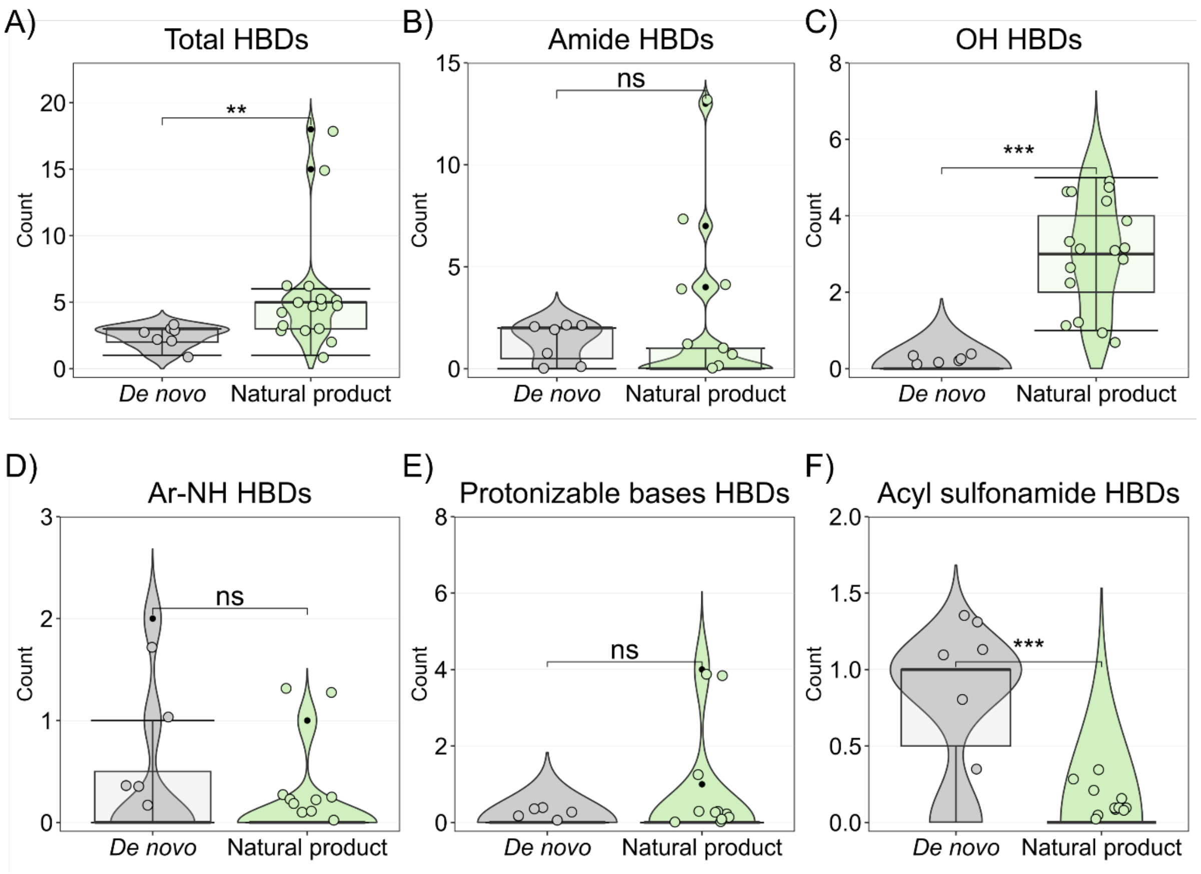

Figure S10. A) Comparison of the number of HBDs in orally bioavailable macrocyclic drugs discovered by *de novo* design (n=7) or from natural products (n=17) for the neutral state. Frequencies of HBDs orginating from (**B**) amides, and (**C**) phenols and aliphatic alcohols, in the two classes of drugs. Frequencies of HBDs orginating from (**D**) nitrogen containing heterocycles, (**E**) protonizable bases and (**F**) acyl sulfonamides. HBDs of bases originate from aliphatic amines, including piperidines, pyrrolidines, piperazines and 1,3-oxazinanes, and also from guanidines. Box plots show the 50th percentiles as horizontal bars, the 25th and 75th percentiles as boxes, the 25th percentile minus 1.5 x the interquartile range and the 75th percentile plus 1.5 x the interquartile range as whiskers. Black dots represent values higher than 1.5 x the interquartile range and less than 3 x the interquartile range at either end of the box. Violin shapes represent the data density at each count value.

Table S8. Macrocycles in clinical trials ordered by therapeutic indication and target.

Therapeutic indication	Drug target	Drug	Origin	Absorption	Most recent clinical trial (FDA)	Clinical trial ID	Clinical trial phase	Year
Antithrombotic	FXIa	Milvexian	*De novo*	Oral	Completed	NCT03891524	II	2021
Dry eye syndrome	Tropomyosin receptor kinase A (TrkA)	Tavilermide	Natural product derivative	Parenteral	Completed	NCT03925727	III	2020
Food-related behavior disease	Gαi/MAPK	Livoletide	Natural product derivative	Parenteral	Completed	NCT03790865	II-III	2020
GI motility	GHSR (Growth hormone secretagogue receptor)	Ulimorelin	Natural product derivative	Parenteral	Completed	NCT02784392	II	2018
Gingivitis	C3 complement	AMY-101	Natural product derivative	Parenteral	Completed	NCT03694444	I-II	2020
Heart disease	Endothelin A receptor (ETA)	BQ-123	Natural product derivative	Parenteral	Recruiting	NCT02966665	I	2023
	Natriuretic peptide receptors (NPRs)	PL-3994	Natural product derivative	Parenteral	Recruiting	NCT04318145	II	2022
	Protein kinase C (PKC)	Ruboxistaurin	*De novo*	Oral	Withdrawn (January 2022, no funding)	NCT02769611	I-II	2022
Infection: Antibacterial	23S rRNA	Solithromycin	Natural product derivative	Oral	Completed	NCT02605122	II-III	2018
	23S rRNA	Nafithromycin	Natural product derivative	Oral	Completed	NCT03981887	I	2019
	Binding to the lipopolysaccharide transport protein D (LptD)	Murepavadin	Natural product derivative	Parenteral	Completed	NCT03409679	III	2019
	RNA polymerase, DNA gyrase and topoisomerase IV	TNP-2092	Natural product derivative	Parenteral	Completed	NCT03964493	II	2020

Infection: Antifungal	β-(1,3)-d-glucan synthase	Rezafungin	Natural product derivative	Parenteral	Completed	NCT03667690	III	2021
Infection: Antiviral	HCV NS5A	Odalasvir	*De novo*	Oral	Completed	NCT02961660	I	2018
Mucositis	NA	Avasopasem manganese	Natural product derivative	Parenteral	Active	NCT03689712	III	2023
Neurodegenerative disease	Protein kinase C (PKC)	Bryostatin 1	Natural product	Oral	Active	NCT04538066	II	2022
Oncology	CDK9, JAK2, FLT3	Zotiraciclib	*De novo*	Oral	Not yet recruiting	NCT05588141	I-II	2027
	CXCR4	Motixafortide	Natural product derivative	Parenteral	Recruiting	NCT04543071	II	2025
	Elongation factor 1-alpha 2 (eEF1A2)	Plitidepsin	Natural product	Parenteral	Completed	NCT03070964	II	2018
	Histone deacetylase (HDAC)	OBP-801	Natural product	Parenteral	Unknown	NCT02414516	I	2019
	Mcl-1	AZD-5991	*De novo*	Parenteral	Completed	NCT03218683	I	2021
	MEK1, FLT3	E-6201	Natural product derivative	Parenteral	Recruiting	NCT05388877	I	2024
	NA (ROS)	Talaporfin	Natural product derivative	Parenteral	Completed	NCT02326454	II	2017
	NA (ROS)	HPPH	Natural product derivative	Parenteral	Completed	NCT03090412	II	2021
	FKBP12, Serine/threonine-protein kinase mTOR	Ridaforolimus	Natural product derivative	Oral	Completed	NCT01256268	I	2017
	Somatostatin receptor 2 (SSTR2)	Satoreotide tetraxetan	Natural product derivative	Parenteral	Active	NCT02592707	II	2025
	Splicing factor 3b subunit 1 (SF3B1)	H3B-8800	Natural product derivative	Oral	Recruiting	NCT02841540	I	2024
	Stimulator of Interferon Genes (STING)	ADU-S100	Natural product derivative	Parenteral	Completed	NCT03937141	II	2021

	Thioredoxin reductases (TrxRs)	Motexafin gadolinium	Natural product derivative	Parenteral	Active	NCT01562223	NA	2022
	Tropomyosin receptor kinases (TRKs)	Selitrectinib	*De novo*	Oral	Active	NCT03215511	I	2025
	Tubulin	Patupilone	Natural product	Parenteral	Unknown	NCT00159484	I-II	2017
	Tubulin	KOSN-1724	Natural product derivative	Oral	Active	NCT01379287	I	2023
Preterm Delivery	Oxytocin receptor gene (OXTR)	Merotocin	Natural product derivative	Parenteral	Recruiting	NCT02545127	II	2022
Septic Shock	Vasopressin receptor 1A (V1AR)	Selepressin	Natural product derivative	Parenteral	Completed	NCT02508649	II-III	2018

Table S9. Subclassification of macrocyclic clinical candidates into original natural products, natural product derivatives and *de novo* designed. Original natural products have been placed first, followed by families of natural product derivatives and *de novo* designed macrocyclic candidates. Clinical candidates belonging to compound families have been grouped together as indicated by the color coding.

Drug	Origin	Subclassification	Improvement for derivative	Description	Reference
Bryostatin 1	Natural product	Original natural product		No data	Halford, B. *Chem. Eng. News Arch.* **2011**, *89 (43),* 10–17.
Plitidepsin	Natural product	Original natural product		No data	Alonso-Álvarez, S.; Pardal, E.; Sánchez-Nieto, D.; Navarro, M.; Caballero, M. D.; Mateos, M. V.; Martin, A. Drug Des. *Devel. Ther.* **2017**, Volume11, 253–264.
OBP-801	Natural product	Original natural product		No data	Shindoh, N.; Mori, M.; Terada, Y.; Oda, K.; Amino, N.; Kita, A.; Taniguchi, M.; Sohda, K.-Y.; Nagai, K.; Sowa, Y.; Masuoka, Y.; Orita, M.; Sasamata, M.; Matsushime, H.; Furuichi, K.; Sakai, T. *Int. J Oncol* **2008**, *32 (3),* 545–555.
Patupilone	Natural product	Original natural product		No data	Rothermel, J.; Wartmann, M.; Chen, T.; Hohneker, *J. Semin. Oncol.* **2003**, *30* (3, Supplement 6), 51–55.
Rezafungin	Natural product	Natural product derivative (anidulafungin)	PK	Longer half life (higher safety)	Sofjan, A. K.; Mitchell, A.; Shah, D. N.; Nguyen, T.; Sim, M.; Trojcak, A.; Beyda, N. D.; Garey, K. W. *J. Glob. Antimicrob. Resist.* **2018**, *14,* 58–64.
ADU-S100	Natural product	Natural product derivative (cGAMP)		No data	Kong, X.; Zuo, H.; Huang, H.-D.; Zhang, Q.; Chen, J.; He, C.; Hu, Y. *J. Adv. Res.* **2023**, *44,* 119–133.
KOSN-1724	Natural product	Natural product derivative (epothilone B)	PD/PK	Third generation oral derivative	Chou, T.-C.; Zhang, X.-G.; Dong, H.; Zhong, Z.; Feng, L.; Li, Y.; Sherrill, M.; Timmermanns, P.; Johnson, R.; Danishefsky, *S. Cancer Res.* **2008**, *68*, 1402.
Ulimorelin	Natural product	Natural product derivative (lead from cyclic peptide library)		No data	Hoveyda, H. R.; Marsault, E.; Gagnon, R.; Mathieu, A. P.; Vézina, M.; Landry, A.; Wang, Z.; Benakli, K.; Beaubien, S.; Saint-Louis, C.; Brassard, M.; Pinault, J.-F.; Ouellet, L.; Bhat, S.; Ramaseshan, M.; Peng, X.; Foucher, L.; Beauchemin, S.; Bhérer, P.; Veber, D. F.; Peterson, M. L.; Fraser, G. L. *J. Med. Chem.* **2011**, *54* (24), 8305–8320.
BQ-123	Natural product	Natural product derivative (lead from cyclic peptide library)		No data	Spatola, A. F.; Crozet, Y.; deWit, D.; Yanagisawa, M. *J. Med. Chem.* **1996**, *39* (19), 3842–3846.
PL-3994	Natural product	Natural product derivative (lead)		No data	Edelson, J. D.; Makhlina, M.; Silvester, K. R.; Vengurlekar, S. S.; Chen, X.; Zhang, J.; Koziol-White, C. J.; Cooper, P. R.; Hallam, T. J.; Hay, D. W. P.; Panettieri, R. A. *I Ther.* **2013**, *26* (2), 229–238.
E-6201	Natural product	Natural product derivative (LL-Z1640-2)	PD/PK	Improved potency and higher bioavailability	Shen, Y.; Boivin, R.; Yoneda, N.; Du, H.; Schiller, S.; Matsushima, T.; Goto, M.; Shirota, H.; Gusovsky, F.; Lemelin, C.; Jiang, Y.; Zhang, Z.; Pelletier, R.; Ikemori-Kawada, M.; Kawakami, Y.; Inoue, A.; Schnaderbeck, M.; Wang, Y. Bioorg. *Med. Chem. Lett.* **2010**, *20* (10), 3155–3157.

Tavilermide	Natural product	Natural product derivative (neurothrophin)		No data	Jain, P.; Li, R.; Lama, T.; Saragovi, H. U.; Cumberlidge, G.; Meerovitch, K. *Exp. Eye Res.* **2011**, *93* (4), 503–512.
Merotocin	Natural product	Natural product derivative (oxytocin)		No data	Manning, M.; Misicka, A.; Olma, A.; Bankowski, K.; Stoev, S.; Chini, B.; Durroux, T.; Mouillac, B.; Corbani, M.; Guillon, G. *J. Neuroendocrinol.* **2012**, *24* (4), 609–628.
AMY-101	Natural product	Natural product derivative (cyclic peptide from phage display)	PK	Superior solubility, efficacy and/or pharmacokinetic profiles	Mastellos, D. C.; Ricklin, D.; Sfyroera, G.; Sahu, A. *Clin. Immunol.* **2022**, *235*, 108785.
H3B-8800	Natural product	Natural product derivative (pladienolide)		No data	Seiler, M.; Yoshimi, A.; P. G.; Buonamici, S. *Nat. Med.* **2018**, *24 (4),* 497–504.
Talaporfin	Natural product	Natural product derivative (porphyrin)		No data	Usuda, J.; Kato, H.; Okunaka, T.; Furukawa, K.; Tsutsui, H.; Yamada, K.; Suga, Y.; Honda, H.; Nagatsuka, Y.; Ohira, T.; Tsuboi, M.; Hirano, T. *J. Thorac. Oncol.* **2006**, *1 (5),* 489–493.
HPPH	Natural product	Natural product derivative (porphyrin)		No data	Wu, D.; Liu, Z.; Fu, Y.; Zhang, Y.; Tang, N.; Wang, Q.; Tao, *L. Oncol Lett* **2013**, *6 (4),* 1111–1119.
Motexafin gadolinium	Natural product	Natural product derivative (porphyrin)		No data	Hashemy, S. I.; Ungerstedt, J. S.; Avval, F. Z.; Holmgren, A. *J. Biol. Chem.* **2006**, *281* (16), 10691–10697.
Murepavadin	Natural product	Natural product derivative (protegrin)		No data	Martin-Loeches, I.; Dale, G. E.; Torres, A. Expert Rev. *Anti. Infect. Ther.* **2018**, *16 (4),* 259–268.
TNP-2092	Natural product	Natural product derivative (rifamycin)	PD	Reduced resistance	Ma, Z.; Lynch, A. S. *J. Med. Chem.* **2016**, *59 (14),* 6645–6657.
Ridaforolimus	Natural product	Natural product derivative (sirolimus)	PK	Improved solubility, stability and bioavailability	Mita, M. M.; Gong, J.; Chawla, S. P. *Expert Rev. Clin. Pharmacol.* **2013**, *6 (5),* 465–482.
Satoreotide tetraxetan	Natural product	Natural product derivative (somatostatin)		No data	Plas, P.; Limana, L.; Carré, D.; Thiongane, A.; Raguin, O.; Mansi, R.; Meyer-Losic, F.; Lezmi, S. *Pharmaceuticals* **2022**, *15 (9),* 1085.
Avasopasem manganese	Natural product	Natural product derivative (superoxide dismutase)		No data	Sishc, B. J.; Ding, L.; Nam, T.-K.; Heer, C. D.; Rodman, S. N.; Schoenfeld, J. D.; Fath, M. A.; Saha, D.; Pulliam, C. F.; Langen, B.; Beardsley, R. A.; Riley, D. P.; Keene, J. L.; Spitz, D. R.; Story, M. D. A *Sci. Transl. Med*. **2021**, *13 (593)*
Motixafortide	Natural product	Natural product derivative (T140)		No data	Tamamura, H.; Fujisawa, M.; Hiramatsu, K.; Mizumoto, M.; Nakashima, H.; Yamamoto, N.; Otaka, A.; Fujii, N. *FEBS Lett.* **2004**, *569 (1–3),* 99–104.
Solithromycin	Natural product	Natural product derivative (telithromycin)	PD/PK	Improved potency and stability	Fernandes, P.; Martens, E.; Bertrand, D.; Pereira, D. *Bioorg. Med. Chem.* **2016**, *24 (24),* 6420–6428.
Nafithromycin	Natural product	Natural product derivative (telithromycin)	PD	Improved potency	Iwanowski, P.; Bhatia, A.; Gupta, M.; Patel, A.; Chavan, R.; Yeole, R.; Friedland, D. *Antimicrob. Agents Chemother*. **2019**, *63 (12).*
Livoletide	Natural product	Natural product derivative	PK	Improved plasma	Julien, M.; Kay, R. G.; Delhanty, P. J. D.; Allas, S.;

		(unacylated ghrelin)		stability and bioavailability	Granata, R.; Barton, C.; Constable, S.; Ghigo, E.; van der Lely, A. J.; Abribat, T. *Eur. J. Pharm. Sci.* **2012**, *47 (4)*, 625–635.
Selepressin	Natural product	Natural product derivative (vasopressin)		No data	Laporte, R.; Kohan, A.; Heitzmann, J.; Wiśniewska, H.; Toy, J.; La, E.; Tariga, H.; Alagarsamy, S.; Ly, B.; Dykert, J.; Qi, S.; Wiśniewski, K.; Galyean, R.; Croston, G.; Schteingart, C. D.; Rivière, P. J.-M. *J. Pharmacol. Exp. Ther.* **2011**, *337 (3)*, 786–796.
Milvexian	*De novo*	Structure based drug design			Dilger, A. K.; Pabbisetty, K. B.; Corte, J. R.; De Lucca, I.; Fang, T.; Yang, W.; Pinto, D. J. P.; Wang, Y.; Zhu, Y.; Mathur, A.; Li, J.; Hou, X.; Smith, D.; Sun, D.; Zhang, H.; Krishnananthan, S.; Wu, D.-R.; Myers, J. E. J.; Sheriff, S.; Rossi, K. A.; Chacko, S.; Zheng, J. J.; Galella, M. A.; Ziemba, T.; Dierks, E. A.; Bozarth, J. M.; Wu, Y.; Crain, E.; Wong, P. C.; Luettgen, J. M.; Wexler, R. R.; Ewing, W. R. *J. Med. Chem.* **2022**, *65 (3)*, 1770–1785.
Ruboxistaurin	*De novo*	Structure based drug design			Jirousek, M. R.; Gillig, J. R.; Gonzalez, C. M.; Heath, W. F.; McDonald, J. H.; Neel, D. A.; Rito, C. J.; Singh, U.; Stramm, L. E.; Melikian-Badalian, A.; Baevsky, M.; Ballas, L. M.; Hall, S. E.; Winneroski, L. L.; Faul, M. M. *J. Med. Chem.* **1996**, *39 (14)*, 2664–2671.
Odalasvir	*De novo*	Structure based drug design			De Clercq, E. Biochem. *Pharmacol.* **2014**, *89 (4)*, 441–452.
Zotiraciclib	*De novo*	Structure based drug design			William, A. D.; Lee, A. C.-H.; Goh, K. C.; Blanchard, S.; Poulsen, A.; Teo, E. L.; Nagaraj, H.; Lee, C. P.; Wang, H.; Williams, M.; Sun, E. T.; Hu, C.; Jayaraman, R.; Pasha, M. K.; Ethirajulu, K.; Wood, J. M.; Dymock, B. W. *J. Med. Chem.* **2012**, *55 (1)*, 169–196.
AZD-5991	*De novo*	Structure based drug design			Tron, A. E.; Belmonte, M. A.; Adam, A.; Aquila, B. M.; Boise, L. H.; Chiarparin, E.; Cidado, J.; Embrey, K. J.; Gangl, E.; Gibbons, F. D.; Gregory, G. P.; Hargreaves, D.; Hendricks, J. A.; Johannes, J. W.; Johnstone, R. W.; Kazmirski, S. L.; Kettle, J. G.; Lamb, M. L.; Matulis, S. M.; Nooka, A. K.; Packer, M. J.; Peng, B.; Rawlins, P. B.; Robbins, D. W.; Schuller, A. G.; Su, N.; Yang, W.; Ye, Q.; Zheng, X.; Secrist, J. P.; Clark, E. A.; Wilson, D. M.; Fawell, S. E.; Hird, A. W. *Nat. Commun.* **2018**, *9 (1)*, 5341.
Selitrectinib	*De novo*	Structure based drug design			Liu, Z.; Yu, P.; Dong, L.; Wang, W.; Duan, S.; Wang, B.; Gong, X.; Ye, L.; Wang, H.; Tian, J. *J. Med. Chem.* **2021**, *64 (14)*, 10286–10296.

Table S10. Macrocycles in clinical trials with approved macrocyclic and non-macrocyclic drugs for the same targets. Macrocyclic clinical candidates directed towards a target for which no drug has been approved have not been included.

Macrocycles in CT	Target	Approved drug	ChEMBL ID	Class	Size category
Tavilermide	Tropomyosin receptor kinase A (TrkA)	Regorafenib	CHEMBL1946170	Non-macrocyclic	Small molecule
Ulimorelin	GHSR (Growth hormone secretagogue receptor)	Macimorelin	CHEMBL278623	Non-macrocyclic	Small molecule
AMY-101	C3 complement	Pegcetacoplan	CHEMBL4298211	Oligopeptide	Cyclic peptide
PL-3994	Natriuretic peptide receptors (NPRs)	Nesiritide	CHEMBL1201668	Oligopeptide	Cyclic peptide
		Vosoritide	CHEMBL3707276	Oligopeptide	Cyclic peptide
Solithromycin	23S rRNA	Many antibacterials		Macrocycle	Small molecule
Nafithromycin	23S rRNA	Many antibacterials		Macrocycle	Small molecule
Odalasvir	HCV NS5A	Ledipasvir	CHEMBL2374220	Non-macrocyclic	Small molecule
		Daclatasvir	CHEMBL2023898	Non-macrocyclic	Small molecule
		Pibrentasvir	CHEMBL3545123	Non-macrocyclic	Small molecule
		Velpatasvir	CHEMBL3545062	Non-macrocyclic	Small molecule
		Elbasvir	CHEMBL3039514	Non-macrocyclic	Small molecule
		Ombitasvir	CHEMBL3127326	Non-macrocyclic	Small molecule
Motixafortide	CXCR4	Plerixafor	CHEMBL18442	Macrocycle	Small molecule
E-6201	MEK1, FLT3 (dual)	Selumetinib	CHEMBL1614701	Non-macrocyclic	Small molecule
		Cobimetinib	CHEMBL2146883	Non-macrocyclic	Small molecule
		Binimetinib	CHEMBL3187723	Non-macrocyclic	Small molecule
		Trametinib dimethyl sulfoxide	CHEMBL2105741	Non-macrocyclic	Small molecule
Ridaforolimus	FKBP12, mTOR	Sirolimus, everolimus and temsirolimus	CHEMBL413, CHEMBL1908360 and CHEMBL1201182	Macrocycle	Small molecule
Satoreotide tetraxetan	Somatostatin receptor 2 (SSTR2)	Pasireotide	CHEMBL3039583	Hexapeptide	Cyclic peptide
		Lanreotide	CHEMBL1201185	Octapeptide	Cyclic peptide

Selitrectinib	Tropomyosin receptor kinases (TRKs)	Cenegermin	CHEMBL4297852	Non-macrocyclic	Protein
		Entrectinib	CHEMBL1983268	Non-macrocyclic	Small molecule
		Larotrectinib	CHEMBL3889654	Non-macrocyclic	Small molecule
Patupilone and KOSN-1724	Tubulin	Cabazitaxel	CHEMBL1201748	Non-macrocyclic	Small molecule
		Vincristine	CHEMBL90555	Non-macrocyclic	Small molecule
		Vinorelbine	CHEMBL553025	Non-macrocyclic	Small molecule
		Ixabepilone	CHEMBL1201752	Macrocycle	Small molecule
		Paclitaxel	CHEMBL428647	Non-macrocyclic	Small molecule
		Vinflunine	CHEMBL2110725	Non-macrocyclic	Small molecule
		Eribulin	CHEMBL1683590	Macrocycle	Small molecule
		Docetaxel	CHEMBL92	Non-macrocyclic	Small molecule
		Vinblastine	CHEMBL159	Non-macrocyclic	Small molecule
		Colchicine	CHEMBL107	Non-macrocyclic	Small molecule
Merotocin	Oxytocin receptor (OXTR)	Oxytocin	CHEMBL395429	Nonapeptide	Cyclic peptide
Selepressin	Vasopressin receptor 1A (V1AR)	Vasopressin	CHEMBL1201528	Nonapeptide	Cyclic peptide

Table S11. Classification of the binding site shape of the targets of the macrocycles in clinical trials (n=5).

Drug	PDB ID	Method	Resolution (Å)	Binding site
Milvexian	7MBO	X-Ray	0.9	Groove
Ruboxistaurin	1UU3	X-Ray	1.9	Groove
Solithromycin	4WWW	X-Ray	3.1	Tunnel
AZD-5991	6FS0	X-Ray	2.3	Pocket
Patupilone	7DAE	X-Ray	2.4	Groove

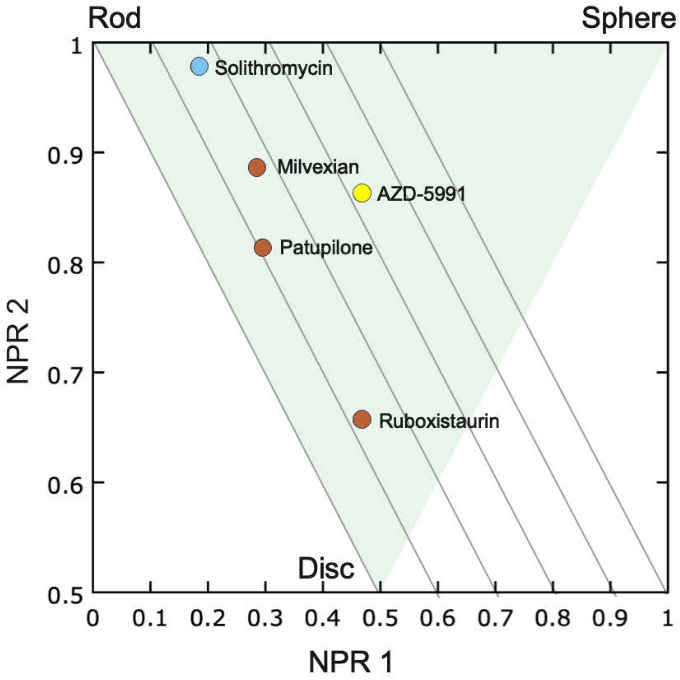

Figure S11. Normalized principal moments of inertia (PMI) plot illustrating the shapes of the target bound conformations of macrocycles in clinical trials (Table S8). Brown, yellow and light blue points stand for groove-, pocket- and tunnel-shaped binding sites, respectively.

A)

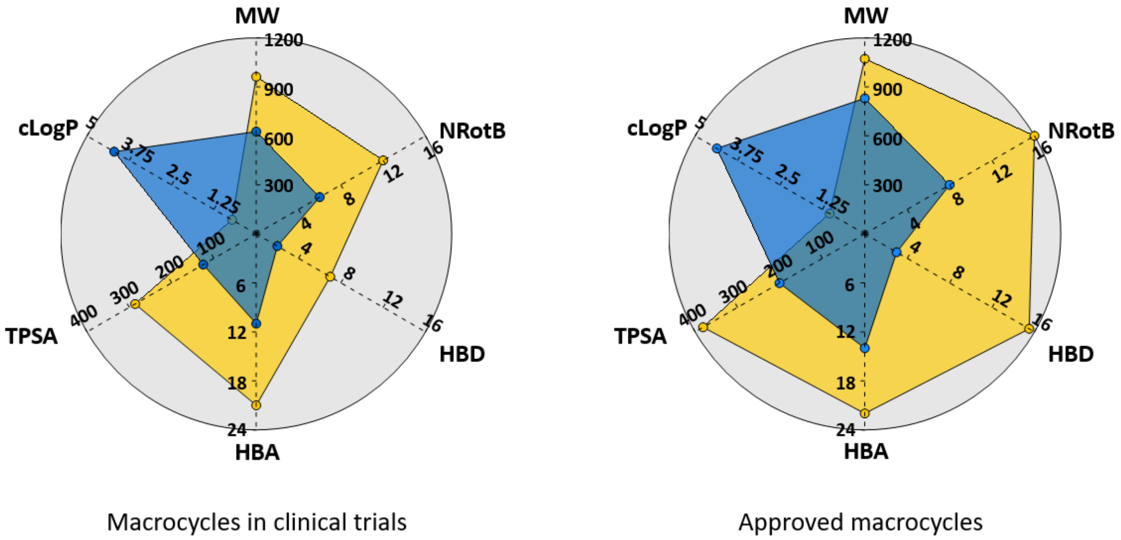

B)

		MW	cLogP	TPSA	HBA	HBD	NRotB
**Clinical trials**	Oral	626.5	4.2	125.4	11	2	6
	Parenteral	962.2	0.7	286.2	21	7	12
**Approved**	Oral	830.1	4.4	201.1	14	3	8
	Parenteral	1072.0	1.0	381.2	22	16	16

Figure S12. Molecular descriptor space of macrocycles in clinical trials and approved as drugs (calculated at pH 7). Median values are reported in the radar plots and in the table.

A)

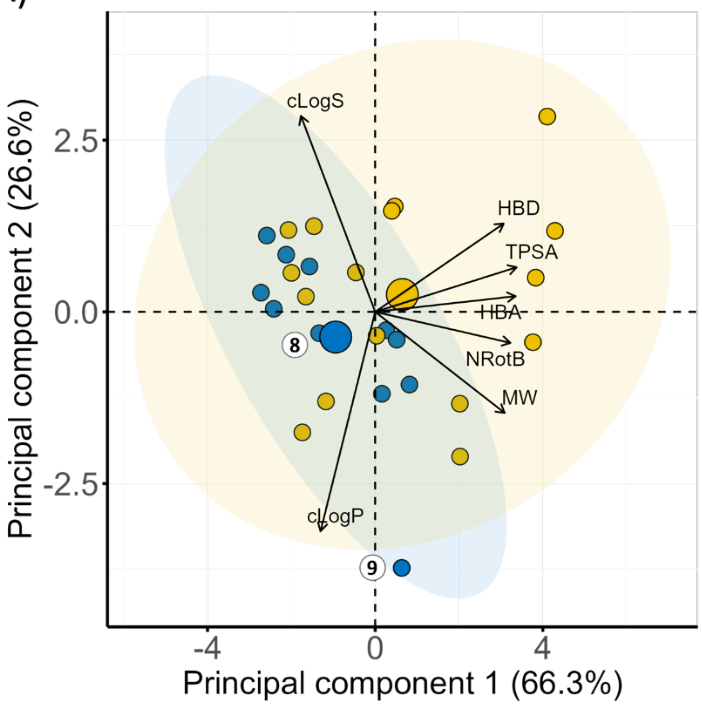

Figure S13. Principal component analysis (PCA) of the chemical space of the clinical trial dataset (n=32) at pH 7. Ellipses in blue and yellow shading show the 95% confidence intervals for orally and parenterally administered macrocycles, respectively. The centroid of each class is indicated with a large circle in the color of the respective class. The contributions of individual descriptors to the PCA are indicated by the length of the arrows. The position of the oral outlier odalasvir (**9**) is indicated, just as that of milvexian (**8**) which is close to the centroid of the oral class. Avasopasem manganese and motexafin gadolinium were removed due to calculation errors with metals.

Table S12. Statistical significances of differences in descriptors between orally and parenterally administered macrocycles in the clinical trials set. Orals and parenterals have been compared by Wilcoxon´s non-parametric test. Descriptors that show statistically significant differences are highlighted in bold.

Property	Group 1	Group2	p	p.signif
**MW**	Oral	Parenteral	0.0417	*
nC	Oral	Parenteral	0.2496	ns
PHI	Oral	Parenteral	0.0615	ns
**NRotB**	Oral	Parenteral	0.0385	*
**HBD**	Oral	Parenteral	0.0019	**
**HBA**	Oral	Parenteral	0.0234	*
**TPSA**	Oral	Parenteral	0.0014	**
**cLogP**	Oral	Parenteral	0.0108	*
NAR	Oral	Parenteral	0.8395	ns
cLogS	Oral	Parenteral	0.123067	0.12

Table S13. Bi-descriptor models for prediction of oral bioavailability applied to the macrocycles in the clinical trial set (n=32).[a]

Model descriptor	HBD and TPSA	**HBD and cLogP[b]**	HBD and MW
Accuracy	0.6	**0.75**	0.63
Sensitivity	1	**0.91**	0.82
Specificity	0.43	**0.67**	0.52
Kappa	0.34	**0.51**	0.29

[a] Predictions were made using HBD $\leq$ 7 as the first cut-off for all three models, in combination with one of TPSA < 292 Å^2, cLogP > 2.22 or MW < 982 Da.
[b] The best model is highlighted in bold.

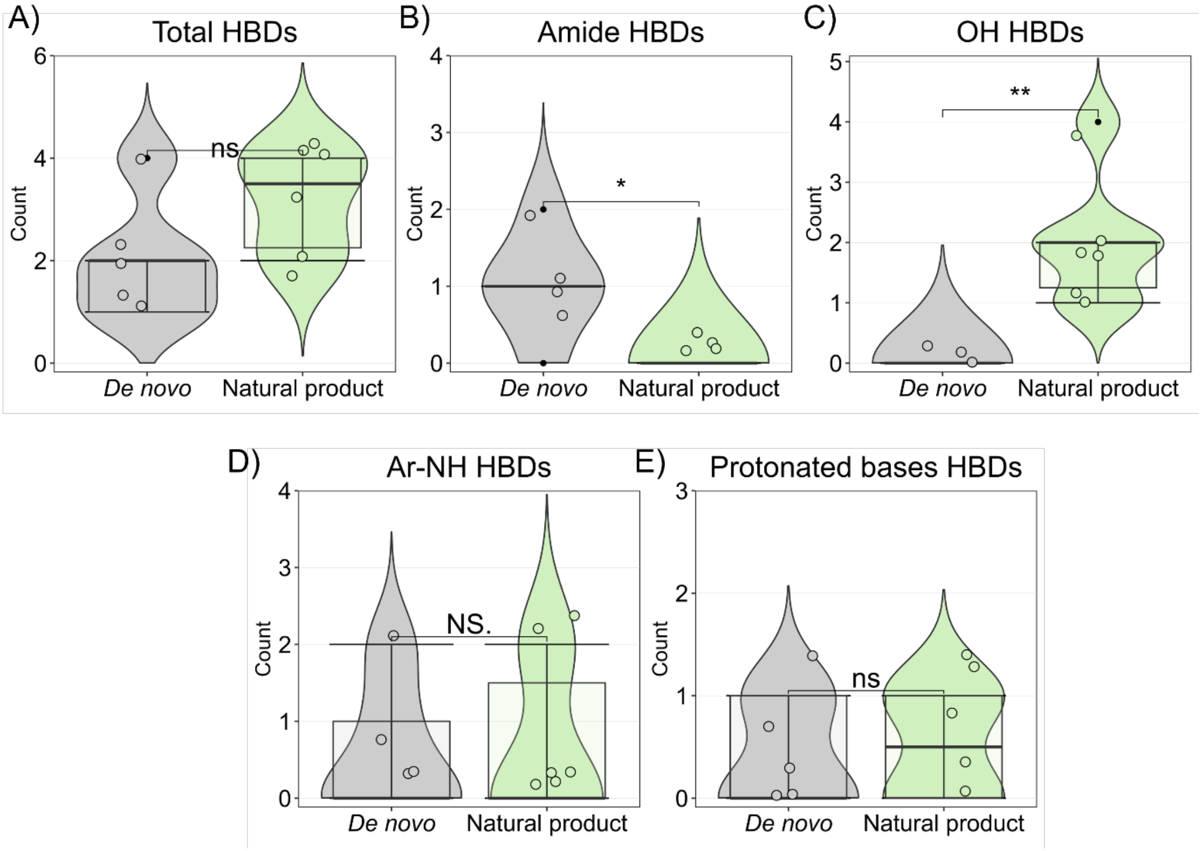

Figure S14. (**A**) Comparison of the total number of HBDs in orally bioavailable macrocycles in clinical trials discovered by *de novo* design (n=5) or from natural products (n=6) at pH 7. Frequencies of HBDs orginating from (**B**) amide groups, (**C**) phenols and aliphatic alcohols, (**D**) nitrogen containing heterocycles and (**E**) protonated bases in the two classes of drugs. HBDs from protonated bases originate from aliphatic amines, including piperidines, pyrrolidines, piperazines and 1,3-oxazinanes as well as from guanidines. NS equals ns when p=1 (Wilcoxon´s test). Box plots show the 50th percentiles as horizontal bars, the 25th and 75th percentiles as boxes, the 25th percentile minus 1.5 x the interquartile range and the 75th percentile plus 1.5 x the interquartile range as whiskers. Black dots represent values higher than 1.5 x the interquartile range and less than 3 x the interquartile range at either end of the box. Violin shapes represent the data density at each count value.

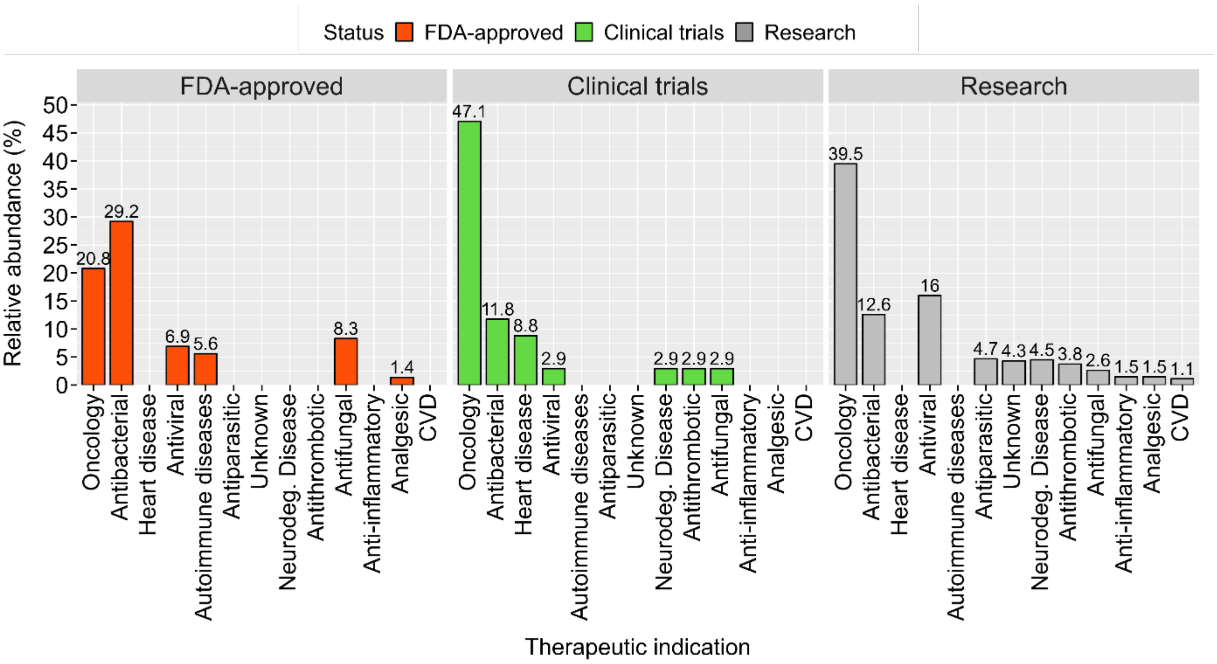

Figure S15. Comparison of the therapeutic indications between FDA-approved macrocyclic drugs (in red, n=72, only the top six indications have been included), macrocycles in clinical trials (in green, n=34, only the top seven indications have been included) and medicinal chemistry articles published between 2005-2022 (in grey, n=532). For the selection of the top seven indications of macrocycles in clinical trials, those with representation in the other two groups were prioritized at equal percentages (2.9%). Abbreviations: CVD: cardiovascular disease, Neurodeg. Disease: neurodegenerative disease.

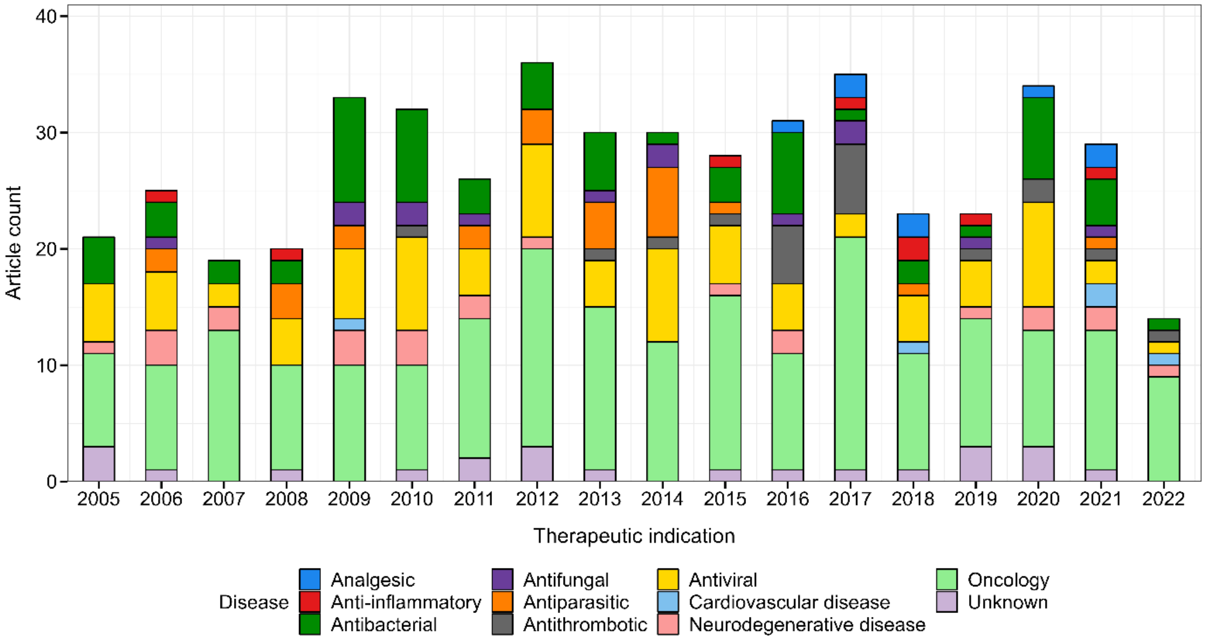

Figure S16. Chronological evolution of therapeutic indications studied in the articles retrieved from the leading 20 journals in medicinal chemistry throughout the years 2005-2022 (n=532). Therapeutic indications with less than 4 articles have been removed from the figure.

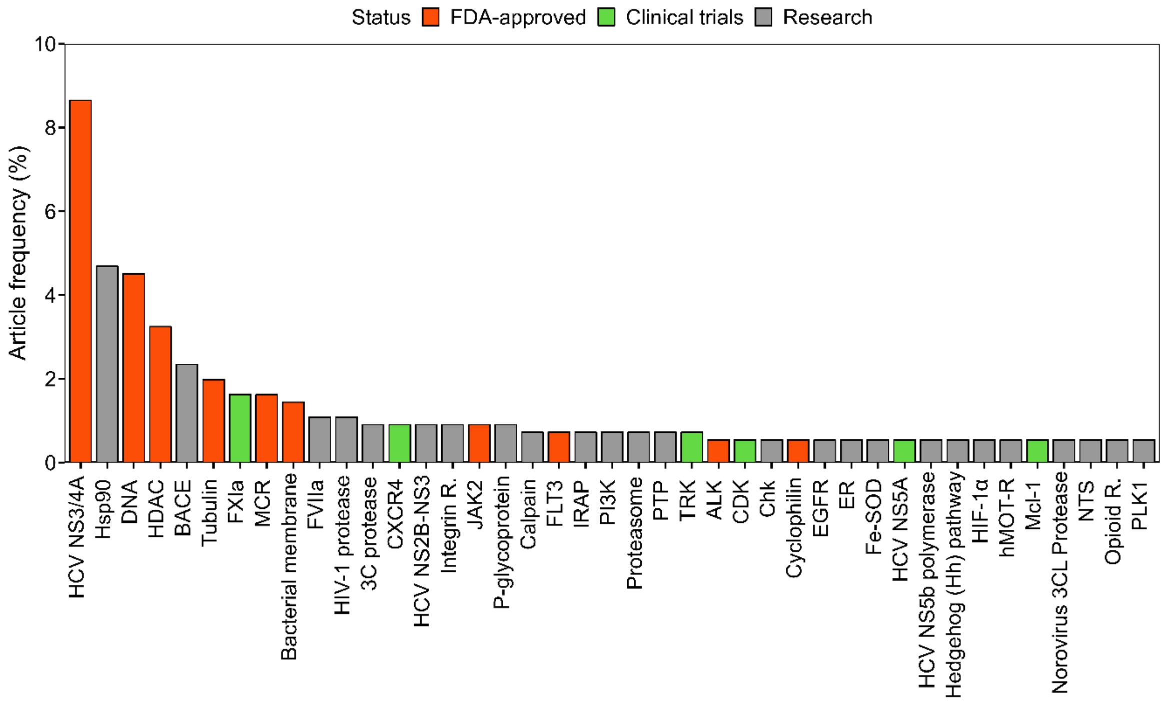

Figure S17. Target frequency in the articles retrieved from the leading 20 journals in medicinal chemistry throughout the years 2005-2022 (n=555). Entries were clustered by target and colored by the discovery phase status, where red indicates a target with at least one approved drug, green a target with a macrocycle in the clinic and grey a target reported in the literature.

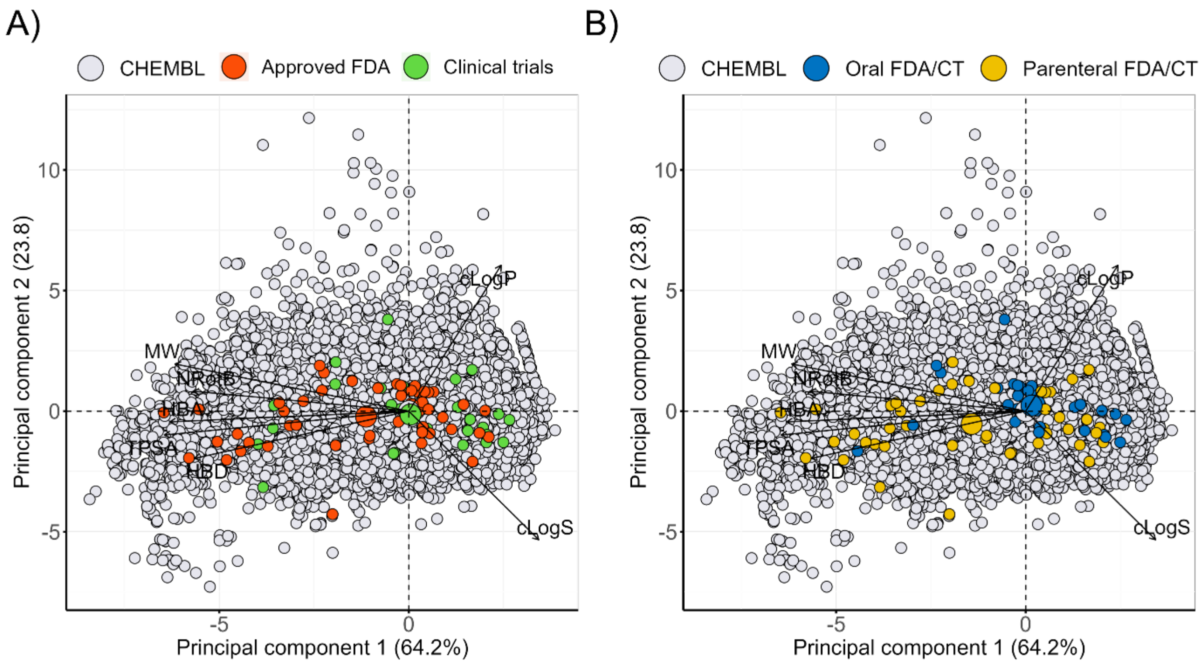

Figure S18. A) Principal component analysis (PCA) of the chemical space of the macrocycles in the ChEMBL dataset against the FDA-approved drugs and clinical candidates macrocycle datasets and **B**) the oral/parenteral parts of these two datasets. Descriptors were calculated from the SMILES structures of the macrocycles at pH 7. Macrocycles with MW >1500 Da were removed from both plots (n=26085). The contributions of individual descriptors to the PCAs are indicated by the length of the arrows.

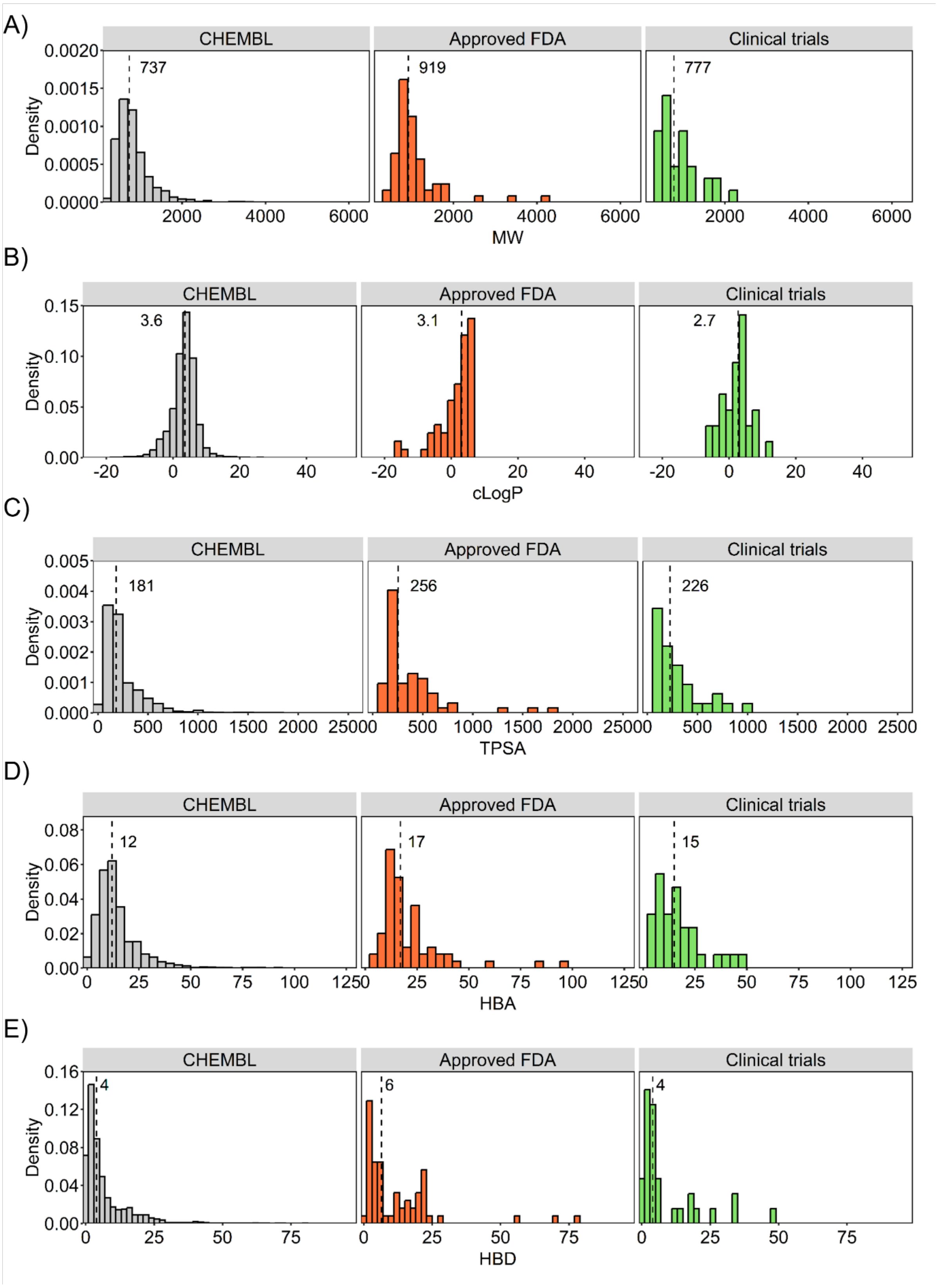

A)
CHEMBL
Approved FDA
Clinical trials
Density
0.0020
0.0015
0.0010
0.0005
0.0000
737
919
777
2000
4000
6000
MW
B)
3.6
3.1
2.7
0.15
0.10
0.05
0.00
-20
0
20
40
cLogP
C)
0.005
0.004
0.003
0.002
0.001
0.000
181
256
226
0
500
1000
1500
2000
2500
TPSA
D)
0.08
0.06
0.04
0.02
0.00
12
17
15
0
25
50
75
100
125
HBA
E)
0.16
0.12
0.08
0.04
0.00
4
6
4
HBD

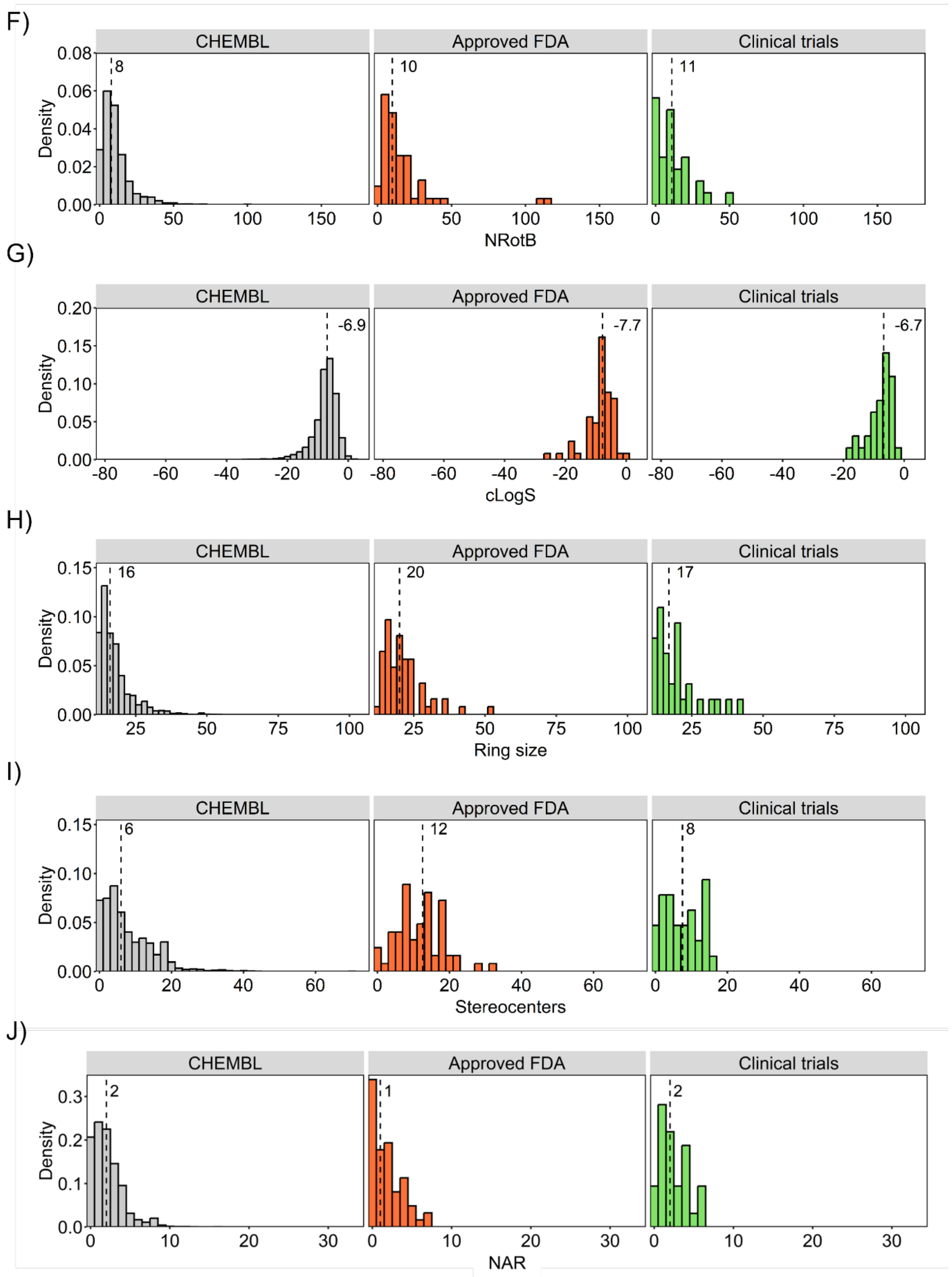

Figure S19. Density histogram plots of descriptors calculated for the ChEMBL dataset (n=28052, in grey), the FDA-approved macrocyclic drugs (n=62, in red) and the macrocycles in clinical trials dataset (n=32, in green). (**A**) MW, (**B**) cLogP, (**C**) TPSA, (**D**) HBA, (**E**) HBD, (**F**) NRotB, (**G**) cLogS, (**H**) macrocycle ring size, (**I**) number of stereocenters and (**J**) number

of aromatic rings (NAR) of the macrocycles in each of the three datasets. The median value for the descriptor is given in each panel and indicated by a dashed line. Descriptors were calculated from the SMILES structures of the macrocycles at pH 7.

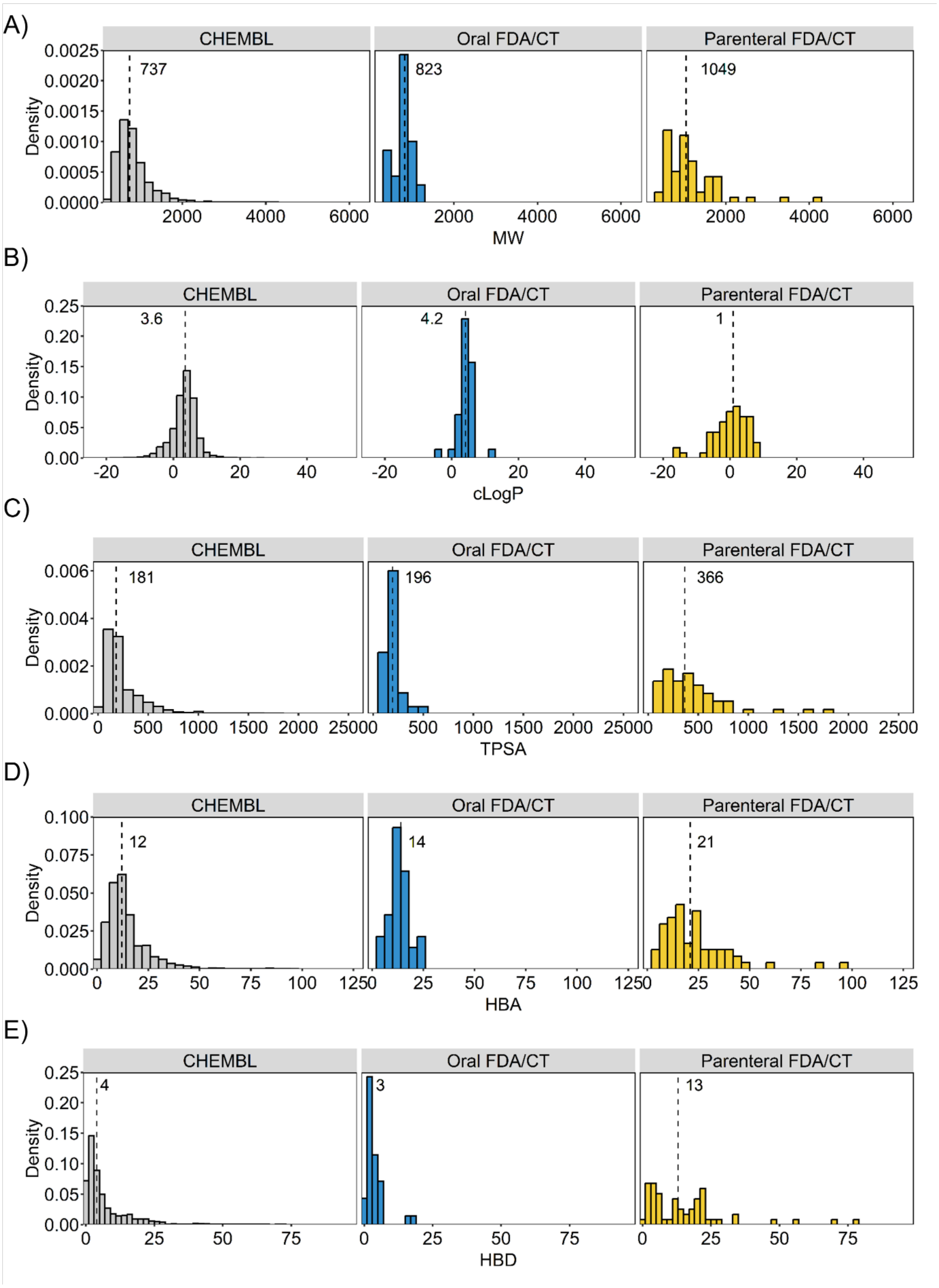

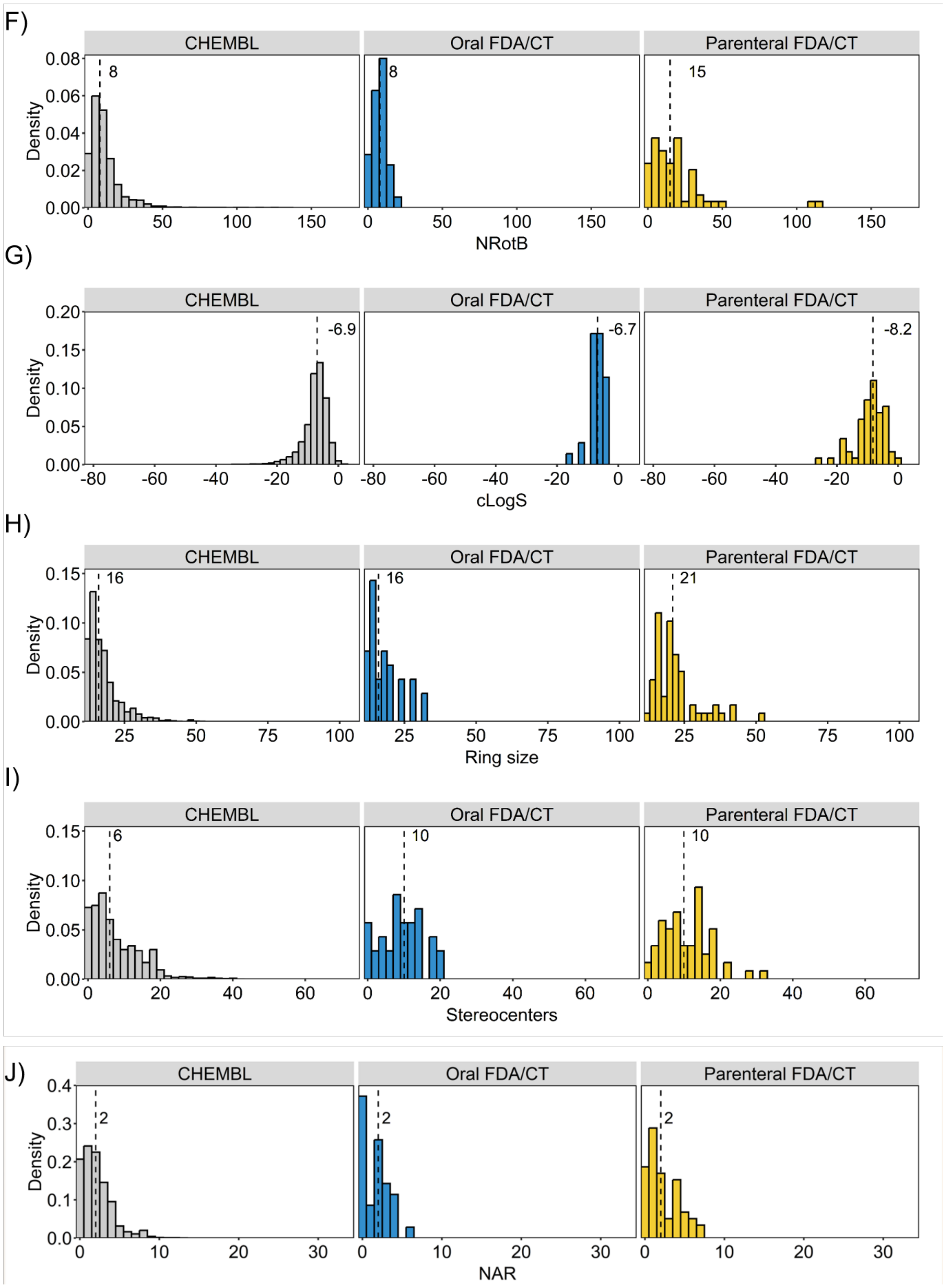

Figure S20. Density histogram plots of descriptors calculated for the CHEMBL dataset (n=28052, in grey) and the combined oral (n=35, in blue) and parenteral (n=59, in yellow) macrocycles in the clinical trial and FDA-approved datasets. (**A**) MW, (**B**) cLogP, (**C**) TPSA, (**D**) HBA, (**E**) HBD, (**F**) NRotB, (**G**) cLogS, (**H**) macrocycle ring size, (**I**) number of

stereocenters and (**J**) number of aromatic rings (NAR) of the macrocycles in each of the three datasets. The median value for the descriptor is given in each panel and indicated by a dashed line. Descriptors were calculated from the SMILES structures of the macrocycles at pH 7.
